# A Promising PET Tracer for Imaging of α_7_ Nicotinic Acetylcholine Receptors in the Brain: Design, Synthesis, and *in Vivo* Evaluation of a Dibenzothiophene-Based Radioligand

**DOI:** 10.3390/molecules201018387

**Published:** 2015-10-09

**Authors:** Rodrigo Teodoro, Matthias Scheunemann, Winnie Deuther-Conrad, Barbara Wenzel, Francesca Maria Fasoli, Cecilia Gotti, Mathias Kranz, Cornelius K. Donat, Marianne Patt, Ansel Hillmer, Ming-Qiang Zheng, Dan Peters, Jörg Steinbach, Osama Sabri, Yiyun Huang, Peter Brust

**Affiliations:** 1 Helmholtz-Zentrum Dresden-Rossendorf, Institute of Radiopharmaceutical Cancer Research, Permoserstraße 15, Leipzig 04318, Germany; E-Mails: r.teodoro@hzdr.de (R.T.); m.scheunemann@hzdr.de (M.S.); b.wenzel@hzdr.de (B.W.); m.kranz@hzdr.de (M.K.); cdonat@nru.dk (C.K.D.); j.steinbach@hzdr.de (J.S.); p.brust@hzdr.de (P.B.); 2 Consiglio Nazionale delle Ricerche, Institute of Neuroscience, Biometra-Institute University of Milan, Via Luigi Vanvitelli 32, Milano 20129, Italy; E-Mails: f.fasoli@in.cnr.it (F.M.F.); c.gotti@in.cnr.it (C.G.); 3 Department of Nuclear Medicine, University Hospital Leipzig, Liebigstraße 18, Leipzig 04103, Germany; E-Mails: marianne.patt@medizin.uni-leipzig.de (M.P.); osama.sabri@medizin.uni-leipzig.de (O.S.); 4 PET Center, Yale University, P.O. Box 208048, 801 Howard Avenue, New Haven, CT 06520-8048, USA; E-Mails: ansel.hillmer@yale.edu (A.H.); ming-qiang.zheng@yale.edu (M.-Q.Z.); henry.huang@yale.edu (Y.H.); 5 Dan PET AB, Rosenstigen 7, Malmö SE-21619, Sweden; E-Mail: info@danpet.eu

**Keywords:** α_7_ nAChR, pharmacophore, positron emission tomography, neuroimaging, fluorine-18

## Abstract

Changes in the expression of α_7_ nicotinic acetylcholine receptors (α_7_ nAChRs) in the human brain are widely assumed to be associated with neurological and neurooncological processes. Investigation of these receptors *in* *vivo* depends on the availability of imaging agents such as radioactively labelled ligands applicable in positron emission tomography (PET). We report on a series of new ligands for α_7_ nAChRs designed by the combination of dibenzothiophene dioxide as a novel hydrogen bond acceptor functionality with diazabicyclononane as an established cationic center. To assess the structure-activity relationship (SAR) of this new basic structure, we further modified the cationic center systematically by introduction of three different piperazine-based scaffolds. Based on *in* *vitro* binding affinity and selectivity, assessed by radioligand displacement studies at different rat and human nAChR subtypes and at the structurally related human 5-HT_3_ receptor, we selected the compound 7-(1,4-diazabicyclo[3.2.2]nonan-4-yl)-2-fluorodibenzo-[*b*,*d*]thiophene 5,5-dioxide (**10a**) for radiolabeling and further evaluation *in* *vivo*. Radiosynthesis of [^18^F]**10a** was optimized and transferred to an automated module. Dynamic PET imaging studies with [^18^F]**10a** in piglets and a monkey demonstrated high uptake of radioactivity in the brain, followed by washout and target-region specific accumulation under baseline conditions. Kinetic analysis of [^18^F]**10a** in pig was performed using a two-tissue compartment model with arterial-derived input function. Our initial evaluation revealed that the dibenzothiophene-based PET radioligand [^18^F]**10a** ([^18^F]DBT-10) has high potential to provide clinically relevant information about the expression and availability of α_7_ nAChR in the brain.

## 1. Introduction

The long-standing interest in molecular imaging of nicotinic acetylcholine receptors (nAChRs) is driven by findings from preclinical and clinical studies, which have demonstrated that dysfunction of neuronal nAChRs is involved in the pathophysiology of many disorders [1]. The nAChRs are a superfamily of ligand-gated ion channels that consist of a pentamer of protein subunits. In the mammalian brain, different subunits assemble with much diversity; however, the α_7_ and α_4_β_2_ nAChR subtypes predominate [2]. The α_7_ subunit is highly expressed in the hippocampus and hypothalamus, as well as in cell types where receptor-mediated ion currents have not been reported. Therefore, α_7_ nAChR-mediated effects such as cognitive enhancement [3] may depend on the ionotropic signaling while other effects may be independent of ion-channel currents [4]. Altered functional availability of α_7_ nAChR has been implicated in a number of diseases of the human central nervous system (CNS), including Alzheimer’s and Parkinson’s disease, schizophrenia and autism, as well as in lung cancer and heart disease [5]. With highly selective ligands, α_7_ nAChRs are approachable targets not only for therapeutic interventions, but also for non-invasive imaging such as Positron Emission Tomography (PET), which can be used to investigate disease pathophysiology *in* *vivo* and support drug development.

**Figure 1:**
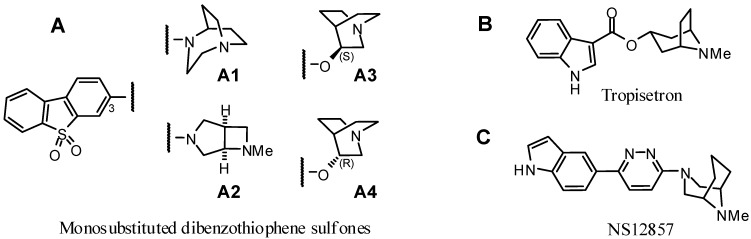
Structures of recent α_7_ nAChR ligands. Four examples of bicyclic amines coupled to dibenzothiophene sulfone (**A**) [15], a tropane derived compound (**B**) [24] and a propylene-bridged *N*-methylpiperazine (**C**) [25] are shown.

While the cationic pharmacophore sufficient for full activation of all neuronal nAChRs is the tetramethylammonium cation, several α_7_ nAChR selective motifs have been identified based on the observation that an additional hydroxyl group present in choline or quinuclidinol activates largely the homomeric α_7_ but not the heteromeric nAChRs [6]. So far, at least nine structurally related families of high-affinity small-molecule ligands of the α_7_ nAChR have been characterized [6,7], including anabaseine-, pyrrolidine-, and diazabicyclononane-based PET radiotracers such as [^11^C]GTS-21 [8], [^11^C]A-844606 and [^11^C]A-582941 [9], [^11^C]CHIBA-1001 [10], [^11^C]NS14492 [11], [^18^F]NS10743 [12] and [^18^F]NS14490 [13]. Recent progress in α_7_ nAChR PET imaging comes from the discovery of the α_7_ nAChR selective binding of the antiviral interferon inducer tilorone [14], containing a tricyclic fluorenone nucleus. Further structural modification by replacing this fluorenone moiety with dibenzothiophene sulfone as alternative hydrogen bond acceptor (HBA) functionality and introduction of 1,4-diazabicyclo[3.2.2]nonane as cationic center resulted in a novel compound with markedly increased binding affinity for α_7_ nAChR (*K*_i_ = 56 nM for tilorone and 0.023 nM for compound **48**, Figure 1, A1) [15]. This discovery by Schrimpf *et al.*, in 2012 prompted us to develop a series of novel derivatives as references for ^18^F-labeled ligands of α_7_ nAChRs and to further investigate the SAR of this new pharmacophore. At the time of working on the radiolabeling of the most promising ligand for PET imaging studies [16], researchers from the Johns Hopkins University had successfully completed the radiosynthetic work on respective ligands [17]. While the evaluation of the resulting [^18^F]ASEM in baboons and humans was published [18,19,20], we felt encouraged by the inherent potential of the Abbott lead structure to continue our research on ^18^F-labelled diazabicyclononane-containing α_7_ nAChR ligands [16,21] by modifying both the HBA functionality and the cationic center.

Besides the elaboration of a more effective approach towards compound [^18^F]**10a** ([^18^F]DBT-10), which we have selected for further development [16] and is identical to compound [^18^F]**7c** reported by Gao *et al.* [17], we have synthesized a series of derivatives of this lead structure to further analyze structural elements that determine the affinity of potential α_7_ nAChR ligands to other pentameric ligand-gated ion channels such as heteromeric nicotinic or 5-HT_3_ receptors. It has been shown previously that the affinity for α_4_β_2_ nAChR of dibenzothiophene-diazabicyclononanes (e.g., [^18^F]ASEM, *K*_i_ = 562 nM [17]) is notably higher than that of oxadiazolyl-substituted diazabicyclononanes (e.g., [^18^F]NS10743 *K*_i_ > 10 μM [12]). Therefore, we replaced the cationic center with alternative tertiary amine motifs. Insertion of three different diazabicycloalkanes resulted in *N*-methyl-substituted derivatives of the ethylene- and propylene-bridged piperazines diazabicyclo[3.2.1]octane and diazabicyclo[3.3.1]nonane, respectively [22,23].

Previous reports have also shown that structural changes on the amino side chain (cationic center) of a given aromatic unit may affect the binding profile with respect to α_7_/α_4_β_2_ nAChR affinity [26,27]. Furthermore, a small series of bicyclic amines coupled to dibenzothiophene sulfone, including chiral quinuclidines (Figure 1, A3 and A4) and a fused azetidine (3,6-diazabicyclo[3.2.0]heptane) (Figure 1, A2) have been investigated by Schrimpf *et al.* [15]. In addition to nitrogen bridgeheaded aza- or diazabicylic molecules derived from quinuclidine or diazabicyclo[3.2.2]nonane, several alkylene bridged piperidines (e.g., tropane derivatives such as tropisetron [24]) or piperazines (e.g., *N*-methyl-3-azagranatanine derivative such as NS12857 [25]) have been identified as potent α_7_ nAChR selective ligands (Figure 1B,C). Considering this, we were inspired to prepare a small series of fluorinated dibenzothiophene sulfones with a set of (three) differently arranged piperazine-based diazabicyclic scaffolds.

To test for the effect of the designed structural modifications and to evaluate the target selectivity of potential α_7_ nAChR imaging probes, the binding affinities of all fluoro-substituted derivatives for the human α_7_ nAChR, as well as the three most important subtypes of heteromeric nAChRs (human α_4_β_2_, human α_3_β_4_, and rat α_6_β_2_*) and the structurally similar 5-HT_3_ receptor, were determined. For the most suitable ligand **10a**, a radiolabeling procedure was developed and successfully implemented, then subsequently optimized and transferred to a fully automated setup. Finally, the pharmacokinetics of [^18^F]**10a** was evaluated in both piglets and monkey by dynamic PET scans under baseline and blocking conditions.

## 2. Results and Discussion

### 2.1. Chemistry

#### 2.1.1. Rationale

1,4-Diazabicyclo[3.2.2]nonane-substituted dibenzothiophene sulfone **10** was found to have exceptional high affinity for α_7_ nAChR and effective brain uptake [15,17]. This prompted us to develop novel derivatives as candidates for ^18^F-labeled ligands and to further investigate the SAR of this new pharmacophore (Table 1).

Initially, the three fluoro-substituted 1,4-diazabicyclo[3.2.2]nonane-containing derivatives **10a**–**c** were designed. While **10a** and **10b** are identical to compounds **7c** and **7a**, respectively, recently published by Gao *et al.* [17], the isomer **10c** has not been described so far. To gain further insight into the SAR of dibenzothiophene-based scaffolds we embarked on the synthesis of a second series of compounds. Previous results on the systematic side chain rigidification have highlighted the importance of conformational restriction of the aminocycle for high α_7_ selectivity [15]. Since the tropane core has been identified as a structural motif that could provide this selectivity [4,6] it was of interest to include a set of three tropane-like diamines into the synthetic plan. With the carbon atom replaced at position 3 both in tropane and in *N*-methylgranatanine [28] by an nitrogen atom, the resulting secondary amino group of each *N*-methyl piperazine building block could serve as position of attachment to the tricyclic arene system.

**Table 1:** Structures of fluoro-substituted dibenzothiophene-based ligands for α_7_ nAChR. 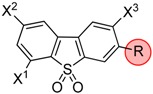

Compound	R	X^1^	X^2^	X^3^
**10**	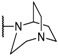	H	H	H
**10a**	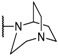	H	F	H
**10b**	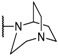	F	H	H
**10c**	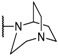	H	H	F
**12a**	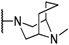	H	F	H
**12b**	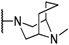	F	H	H
**13b**	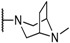	F	H	H
**14b**	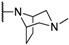	F	H	H

Four novel fluorine-substituted derivatives were envisioned, in which the homopiperazine-based 1,4-diazabicyclo[3.2.2]nonane was replaced by conformationally restricted piperazine-based substituents, bridged by either ethylene (8-methyl-3,8-diazabicyclo[3.2.1]octane = azatropane (**13b**), 3-methyl-3,8-diazabicyclo[3.2.1]octane (**14b**)) or propylene units (9-methyl-3,9-diazabicyclo[3.3.1]nonane = *N*-methyl-3-azagranatanine (**12a**,**b**)) [22].

#### 2.1.2. Synthesis of α_7_ nAChR Ligands

**Scheme 1:**
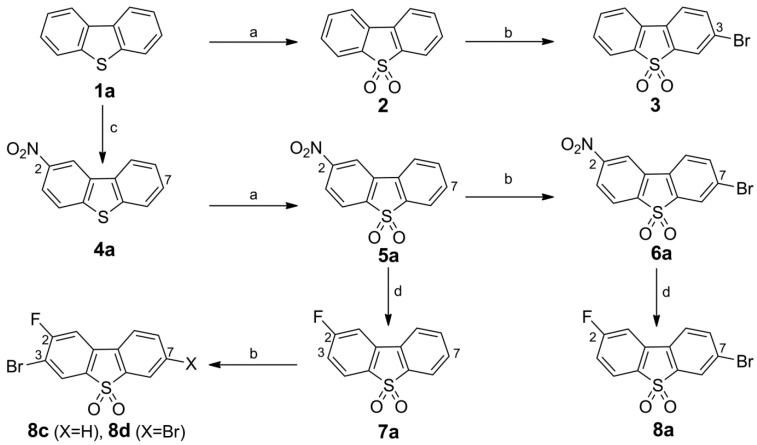
Synthesis of 3-bromo dibenzothiophene intermediates **3**, **6a**, **8a** and **8c**. Reagents and conditions: (a) H_2_O_2_ (50%), HOAc, 120 °C, 5 h, 97% for **2** and 96% for **5a** (b) NBS, conc. H_2_SO_4_, r.t., 24 h, 44% for **3**, 64% for **6a**, 46% for **8c**, and 18% for **8d**; (c) HNO_3_ (65%), HOAc, 20–40 °C, 24 h, 26% for **4a**; (d) TMAF·4 H_2_O, cyclohexane/DMSO, ↑↓, 6 h (azeotropic drying with separation of H_2_O), compound **5a** or compound **6a**, 95 °C, 5 h, 75% for **7a** and 74% for **8a**.

**Scheme 2:**
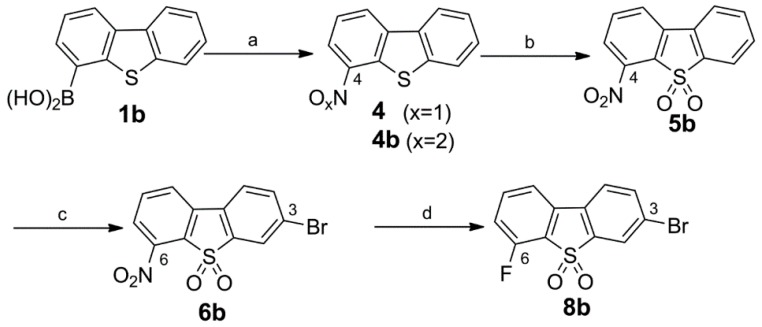
Synthesis of 3-bromo dibenzothiophene intermediates **6b** and **8b**. Reagents and conditions: (a) *tert*-butyl nitrite, MeCN, 55 °C, 24 h, mixture of **4**/**4b** (~88:12); (b) H_2_O_2_ (50%), HOAc, 120 °C, 5 h, 72% (two steps); (c) NBS, conc. H_2_SO_4_, r.t., 24 h, 78%; (d) TMAF·4H_2_O, cyclohexane/DMSO, 6 h (azeotropic drying with separation of H_2_O), compound **6b**, 95 °C, 5 h, 73%.

Dibenzo[*b*,*d*]thiophene (**1a**) and dibenzo[*b*,*d*]thiophene 4-boronic acid (**1b**) served as starting materials for the synthesis of dibenzo[*b*,*d*]thiophene-5,5-dioxides functionalized in the *para-* (Scheme 1) or *ortho*- position (Scheme 2) to the sulfone group. The final coupling to the *N*-atom of a diazabicyclic moiety was performed at position 7 or 3 of the respective tricyclic scaffold to afford the potential α_7_ nAChR ligands **10**, **10a**–**c**, **12a**,**b**, **13b**, and **14b**, as shown in Table 1.

##### Synthesis of Bromo Dibenzothiophene Intermediates **3**, **6a** and **8a**–**8c**

The brominated intermediates were obtained from dibenzo[*b*,*d*]thiophene (**1a**) by three different routes. The 3-bromo-derivative **3** was prepared via sulfide to sulfone oxidation of **1a** with H_2_O_2_ in acetic acid followed by bromination with an excess of **2** in the NBS/H_2_SO_4_ system in 44% yield [29]. Isolation of compound **3** from the 3,7-dibrominated by-product (not shown) along with unreacted starting material was obtained by fractional crystallization of the dibromo compound, followed by a chromatographic separation of pure **3** from the filtrate. The 2-nitrodibenzothiophene sulfone **5a** was prepared by nitration of **1a** using HNO_3_ (65%) in acetic acid [30,31] affording the 2-nitro compound **4a** in moderate yield (26%), followed by oxidation with H_2_O_2_ in acetic acid to give **5a** [32]. The 2-nitro-7-bromo-dibenzothiophene sulfone **6a** was obtained by bromination of **5a** according to the corresponding conversion of **2** to **3**. Because the one benzo ring in **5a** is strongly deactivated by the NO_2_ and SO_2_R groups, no over-brominated by-products were detected and the 2-nitro-7-bromo-dibenzothiophene sulfone **6a** was afforded in 64% yield. To obtain different mono-fluorinated building blocks in a short synthesis, the fluorine substitution either at positions *para* or *ortho* to the sulfone were intended to be carried out with a simple uniform synthetic pathway. In addition to the known three-step conversion of **6a** into the 2-fluoro-7-bromo derivative **8a** based on the Balz-Schiemann reaction with 45% overall yield [17], we deemed a one-step conversion from **6a** to **8a** via fluorodenitration as preferable [33,34]. Tetramethylammonium fluoride (TMAF) proved to be a good source for active fluoride after azeotropic drying of commercial TMAF tetrahydrate in refluxing cyclohexane/DMSO for 6 h. A smooth conversion of **6a** occurred at 95 °C within 5 h to give **8a** in 75%yield.Likewise, the nitro group in the 2-nitrodibenzothiophene 5,5-dioxide **5a** was displaced by fluorine to give **7a** in 74% yield. The vicinal 3-bromination of **7a** was accomplished by applying NBS in H_2_SO_4_ (98%) at room temperature for 24 h to give compound **8b**. In addition to **8c**, the 3,7-dibromo derivative **8d** was detected. Chromatographic separation gave **8d** in 18% yield along with 2-fluoro-3-bromo compound **8c** in 46% yield.

##### Synthesis of the 3-Bromo Dibenzothiophene Intermediates **6b** and **8b**

As result of the two-step synthesis of **5a** from **1a** (Scheme 1), the *ortho*-derivative **4b** was concomitantly formed as a minor nitration product. However, because it was difficult to separate from the excess of **4a**, this approach was considered inappropriate to gain an adequate amount of the 4-nitro dibenzothiophene sulfone **5b**. The intermediate **5b** was recently reported [17]. The described procedure for **4b** is a nitration of commercially available arylboronic acid **1b** with two equivalents of Bi(NO_3_)_3_ as described by Maiti *et al.* [35]. In contrast, we successfully applied a metal-free approach by performing an *ipso* nitrosation of **1b** with *tert*-butyl nitrite in acetonitrile [36,37]. As shown in Scheme 2, the nitroso derivative **4** was formed as the main product along with a minor amount of **4b**. Without further purification, the **4**/**4b** mixture was readily oxidized in a one-pot reaction with H_2_O_2_/acetic acid to give **5b** in 72% yield over two steps from **1b**. After bromination, the resulting **6b** was converted directly into **8b** in 73% yield via our fluorodenitration protocol, in contrast to the earlier described independent four-step synthesis applying a Pschorr reaction [17].

##### Synthesis of **10**, Fluorinated Reference Compounds **10a**–**c**, and Nitro Precursor **11**

**Scheme 3:**
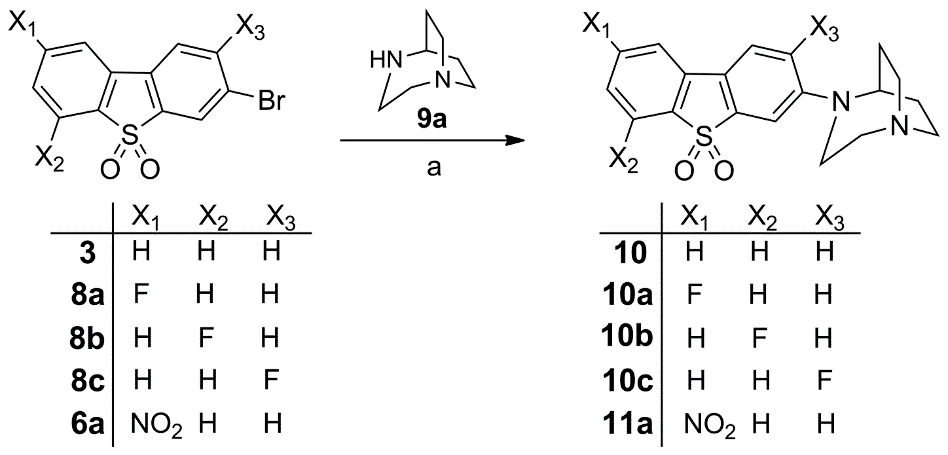
Synthesis of **10**, fluorinated reference compounds **10a**–**c**, and nitro precursor **11**. Reagents and conditions: (a) Pd_2_(dba)_3_, BINAP, Cs_2_CO_3_, toluene, 90 °C, 24–36 h, 52% for **10a**, 48% for **10b**, 44% for **10c**, 73% for **10**, and 53% for **11a**.

The 3-bromodibenzo*[b,d]*thiophene sulfones **3**, **6a** and **8a**–**c** were reacted with 1,4-diazabicyclo[3.2.2]nonane (**9a**) under Pd-catalyzed Buchwald-Hartwig conditions to provide the previously reported compounds **10a** and **10b** [17] and the novel fluorinated isomer **10c** in 52%, 48% and 44% yields, respectively. The non-fluorinated compound **10** [15] and the nitro derivative **11a** [17] were also obtained under Buchwald-Hartwig conditions in 73% and 53% yields, respectively, as depicted in Scheme 3.

##### Synthesis of the Fluorinated Reference Compounds **12a**,**b**, **13b**, and **14b**

**Scheme 4:**
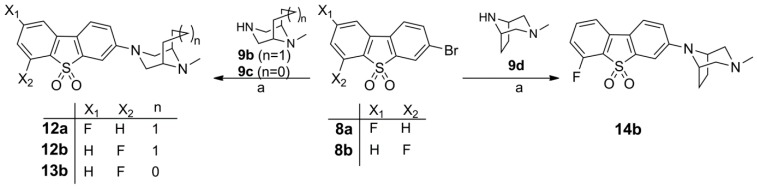
Synthesis of the fluorinated reference compounds **12a**,**b**, **13b**, and **14b**. Reagents and conditions: (a) Pd_2_(dba)_3_, BINAP, Cs_2_CO_3_, toluene, 90 °C, 24–36 h, 38% for **12a**, 42% for **12b**, 47% for **13b**, and 67% for **14b**.

In order to expand the structural diversity of compounds derived from the novel pharmacophore dibenzothiophene for SAR characterization, in a second series of compounds the homopiperazine-based 1,4-diazabicyclo[3.2.2]nonane moiety was replaced by piperazine-based substituents, bridged by either ethylene or propylene units. The bromine substituent in the *meta* position to the sulfone group was used to incorporate these more rigid diamines. The Buchwald-Hartwig coupling of 3-bromodibenzo[*b*,*d*]thiophene sulfones **8a** and **8b** with 9-methyl-3,9-diazabicyclo[3.3.1]nonane (**9b**), 8-methyl-3,8-diazabicyclo[3.2.1]octane (**9c**) and 3-methyl-3,8-diazabicyclo[3.2.1]octane (**9d**) gave compounds **12a**,**b**, **13b**, and **14b** in moderate yields (Scheme 4).

All final compounds (**10**–**14**) are crystalline solids and were fully characterized by NMR spectroscopy (^1^H, ^13^C, ^19^F, COSY, HSQC) and high resolution mass spectrometry.

### 2.2. In Vitro Affinity Assays

All compounds were evaluated *in* *vitro* to measure their affinity and selectivity for the target receptor α_7_ nAChR in relation to the heteromeric receptor subtypes α_4_β_2_, α_6_β_2_, and α_3_β_4_. The results of the respective binding assays are summarized in Table 2.

**Table 2:** *in* *vitro* binding affinities towards human homomeric α_7_, heteromeric α_4_β_2_ and α_3_β_4_ nAChR, rat α_6_β_2_* nAChR subtypes, and human 5-HT_3_ receptor.

Compound	Affinity (*K*_i_ in nM)					Selectivity (*K*_i_ ratio)	
	nAChR Subtype				5-HT_3_^d,e^		
	hα_7_^a^	hα_4_β_2_^b^	hα_3_β_4_^b^	rα_6_β_2_* ^c^		α_7_/α_4_β_2_	α_7_/α_3_β_4_
**10**	0.51 ± 0.32	318 ± 43.3	49.6 ± 14.7	517 ± 186	(35%)	623	97
**10a**	0.60 ± 0.44	517 ± 375	119 ± 29.0	589 ± 217	440 (2%)	862	198
**10b**	0.84 ± 0.16	211 ± 108	42.3 ± 4.73	435 ± 152	(42%)	251	50
**10c**	8.53 ± 1.74	507 ± 212	279 ± 24.4	1390 ± 340	(26%)	59	33
**12a**	30.9 ± 8.72	141 ± 11.7	96.0 ± 1.48	1180 ± 360	(10%)	5	3
**12b**	105 ± 23.9	301 ± 148	94.8 ± 6.48	1190 ± 230	(11%)	3	1
**13b**	40.9 ± 7.77	426 ± 197	224 ± 51.7	2260 ± 540	(10%)	10	5
**14b**	9.26 ± 2.23	>4000	>5000	1450 ± 370	(4%)	>400	>500

^a^ Human α_7_ nAChR in stably transfected SH-SY5Y cells, with radiotracer [^3^H]methyllycaconitine (0.5–1 nM), *K*_D_ = 2.0 nM. ^b^ Human α_4_β_2_ and α_3_β_4_ nAChR in stably transfected HEK-293 cells, with radiotracer [^3^H]epibatidine (0.5–1 nM), *K*_D_ = 0.025 nM for hα_4_β2 nAChR, *K*_D_ = 0.117 nM for hα_3_β_4_ nAChR. ^c^ Rat α_6_β_2_* obtained from rat striatum by immunoimmobilization using anti-rα_6_ nAChR antibody, with radiotracer [^3^H]epibatidine (0.1 nM), *K*_D_ = 0.025 nM. ^d^ *K*_i_ value in nM; human 5-HT_3_ receptor recombinant-HEK293 cells, with radiotracer [^3^H]GR65630 (working concentration *n* = 0.69 nM; *K*_D_ = 0.2 nM). ^e^ percentage of inhibition at 0.1 μM concentration of test compound.

#### 2.2.1. Affinity for α_7_ nAChR

The affinity for α_7_ nAChR was assessed for the human receptor protein expressed in a stably transfected cell line and labeled with the selective α_7_ nAChR antagonist [^3^H]methyllycaconitine. The affinity of the lead of the series (compound **10)**, *K*_i_ = 0.51 nM, is almost identical to the value reported by Gao *et al.*, obtained on [^125^I]α-bungarotoxin labelled rat cortical membranes. The binding affinities of the fluoro-substituted compounds **10a** (*K*_i_ = 0.6 nM *vs.* 1.4 nM of compound **7c** [17]) and **10b** (*K*_i_ = 0.84 nM *vs.* 0.4 nM of compound **7a** [17]) are also comparable with previously published results. All novel fluoro-containing compounds **10c**, **12a**,**b**, **13b**, and **14b** bind with remarkably lower affinity to α_7_ nAChR. Interestingly, the vicinal substitution of the dibenzothiophene with both the fluorine and the diazabicycle is inappropriate in terms of binding as reflected by the 2-fluoro-3-amino derivative **10c**, which possesses an about 10-fold lower affinity at the α_7_ subtype relative to the 2-fluoro-7-amino derivative **10a** and the 6-fluoro-3-amino derivative **10b**.

In particular, we assume that steric effects impair the interactions between the cationic center of compound **10c** and the binding site of α_7_ nAChR [38]. Furthermore, as noticed for the new series of dibenzothiophene derivatives (**12a**,**b**, **13b** and **14b**) an increase in the flexibility of the tertiary amine in the cationic center is also not tolerated. Replacement of the NC-bridged homopiperazine moiety, containing a bicyclic tertiary amine as a basic structural element of the reference compound **10** by three different CC-bridged piperazine ring systems and carrying a methyl-substituted tertiary amino group, has negative effects. The 9-methyl-3,9-diazabicyclo[3.3.1]nonane substituted compounds **12a** and **12b** have significantly diminished affinity for α_7_ nAChR relative to the matched pairs of 1,4-diazabicyclo[3.2.2]nonane-substituted **10a** and **10b**. A similar effect is observed by substitution with an azatropane in compound **13b** (8-methyl-3,8-diazabicyclo[3.2.1]octane) and its isomer **14b** (3-methyl-3,8-diazabicyclo[3.2.1]octane).

Thus, in terms of affinity for the α_7_ nAChR we considered compound **10a** as the most suitable compound reported herein. The structural modifications within the cationic center of the molecules, which we performed to increase selectivity by reducing off-target binding, unexpectedly impaired the binding to α_7_ nAChR to such an extent that the resulting compounds were not included in the development of radioligands within this study.

#### 2.2.2. Affinities for α_4_β_2_, α_3_β_4_, and α_6_β_2_ nAChR

To evaluate off-target binding of the compounds towards heteromeric nAChR subtypes, respective binding assays were performed with [^3^H]epibatidine-labeled human α_4_β_2_ or human α_3_β_4_ nAChR, both stably expressed on human HEK cells, as well as native rat α_6_β_2_* nAChR immobilized by a subunit-specific antibody.

Besides the α_4_β_2_* nAChR, the α_6_β_2_* nAChR belongs to the high-affinity family of β_2_-containing nAChRs [39]. While functionally similar to α_4_β_2_* nAChR, the expression of the α_6_β_2_* subtype is rather selective, primarily in dopamine neurons in the brain [40,41,42,43]. Overlapping expression of α_7_ nAChR with α_4_β_2_* nAChR and also α_6_β_2_* nAChR within the mesolimbic axis [44,45] requires careful analysis of subtype affinity of radioligands for nAChR. In heteromeric nAChR subtypes, the orthosteric ligand binding site is formed at the interface of an α subunit (principal component) and an adjacent non-α subunit (complementary component), equivalent to the (+) surface and the (−) surface of an α subunit in homomeric nAChRs [46,47].

Consistent with the observation that in heteromeric neuronal nAChRs the non-α subunit is of particular importance [38], as it makes the affinity for nicotine dependent on the presence of a β_2_ subunit regardless of the α subunit [47], the herein investigated compounds bind with generally moderate (*K*_i_ values > 100 nM) and almost equal affinities towards both human α_4_β_2_ and rat α_6_β_2_* nAChRs. It is worth noting that the affinities for the α_4_β_2_ subtype of the α_7_ nAChR ligands investigated herein, with the exception of **14b**, are significantly higher than those of the compounds in the NeuroSearch (NS) series [12]. Because these compounds contain identical cationic centers, differences in the receptor subtype binding affinities are probably related to differences in the HBA and hydrophobic functionalities. In the NeuroSearch compound series, an oxadiazole moiety appears as HBA which is spatially more separated from and not forced in plane with the hydrophobic fluorophenyl moiety, while compounds **10a**–**10c** possess fused functionalities with both the HBA and the hydrophobic moiety represented by the fluorine-substituted dibenzothiophene dioxide ring system. We hypothesize that in particular the sulfonyl moiety of these novel compounds promotes binding to the α_4_β_2_ subtype, comparable to the carbonyl moiety acting as a hydrogen acceptor along with a cationic pharmacophore element in the off-target binding of α_7_ nAChR ligand CHIBA-1001 [48,49]. However, for the most selective fluorine-containing ligand of the current study, compound **10a** (selectivity > 800), interfering effects on α_7_ nAChR PET imaging due to its binding to β_2_-containing receptor subtypes are highly unlikely. The conformational changes due to the shift of the bridgehead carbons in the diazabicyclo[3.2.1]octane that we assume to contribute to the significantly improved selectivity of compound **14b** in comparison to **13b** will be analyzed in future studies. It appears that the basic *N*-methyl group is sterically more deshielded in contrast to the azatropane **13b**, resulting in a detrimental effect both on α_4_β_2_ and α_3_β_4_ nAChR binding with only weakly attenuated affinity to the α_7_ subtype (Table 2).

An overlap in the receptor expression between α_7_ and α_3_β_4_* nAChRs, in particular within autonomic neurons [50,51], necessitates the investigation of the selectivity of potential ligands. By comparing data obtained for compounds **10a** and **10b** (*K*_i_: 119 nM and 43 nM) with the affinity values of the corresponding compounds **7c** and **7a** reported by Gao *et al.*, (*K*_i_: 5000 nM and 709 nM) [17], species-specific differences become obvious. Although the most selective compound **10a** (selectivity ~200) binds with sufficiently low affinity to human α_3_β_4_ nAChRs to afford imaging of the α_7_ subtype in human brain, more than 10-fold higher affinities at the human than the rat receptor subtype illustrate the significance of species-specific targeting at the early stage of the development of PET ligands.

The *in* *vitro* binding affinity of all compounds from this series at the 5-HT_3_ receptor, as determined by percentage of inhibition of the binding of [^3^H]GR65630 at 10 μM, indicates high α_7_ nAChR over 5-HT_3_ selectivity (Table 2). From this direct comparison a much higher affinity of ASEM (**10b**) than that of **10a** for 5-HT_3_ may be expected considering the inhibition data at 100 nM (2% for **10a**, 42% for ASEM). Based on this value, compound **10a** is assumed to possess an at least fivefold higher selectivity than all other compounds, except **14b**, reported herein. The *K*_i_ of **10a** was estimated to be 440 nM for 5-HT_3_.

In addition, investigation of interaction of **10a** at 1 μM with further off-targets (α_1_ nAChR, SERT, DAT, NET, VMAT, and choline transporter) by radioligand binding assays revealed no significant binding. Altogether, the target affinity and nAChR subtype specificity of **10a**, the most suitable fluorine-substituted α_7_ nAChR ligand reported herein, is certainly sufficient to ensure that assessment of α_7_ nAChR in humans will not be confounded by binding of the respective PET radiotracer to relevant off-target sites. Therefore, compound **10a** was selected for radiolabeling and further evaluation *in* *vivo*.

### 2.3. Optimization of Manual Radiosynthesis of *[^18^F]**10a*** for Translation to an Automated Module

**Scheme 5:**
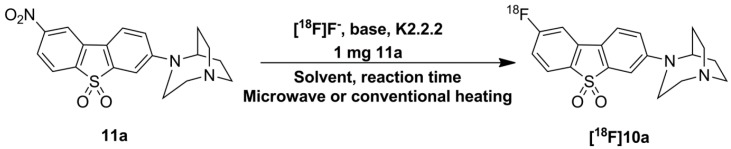
Radiosynthesis of [^18^F]**10a**. Solvents tested: MeCN, DMSO, and DMF at different temperatures with microwave irradiation or conventional heating.

Manual radiosynthesis of [^18^F]**10a** was performed starting from the nitro precursor **11a** and systematically optimized by varying the base, solvent, reaction time and heating system (Scheme 5). The amount of nitro precursor **11a** was kept constant at 1 mg according to our previous practice [52]. A nitro-to-fluoro substitution of activated aryl moieties in general requires moderately high temperatures (e.g., 130–160 °C) [53], reflected by low labeling efficiencies of <1% at 90 °C using MeCN. With increasing temperature (150 °C) and the use of DMSO, labeling efficiency of up to 30% were achieved.

In contrast to decomposition of the corresponding [^18^F]**7c** reported by Gao *et al.* [17], we did not observe decomposition of [^18^F]**10a** in the presence of the strong base potassium carbonate in DMSO at this temperature. In an attempt to improve labelling efficiencies prior to high-scale production of [^18^F]**10a** in a commercially available automated device, we investigated the aromatic radiofluorination of **11a** using DMF as solvent based on our recent experience [52] under different conditions (thermal *vs.* microwave heating). Influence of the basicity of the metal salt on the labelling yield (potassium carbonate *vs.* potassium oxalate) was also investigated.

Under thermal heating and the use of K_2_CO_3_, we determined a time- and temperature-dependent increase in the nitro-to-fluoro conversion with maximum labelling efficiencies of about 90% after 10 min of reaction time at 120 °C. Under these conditions, K_2_CO_3_ is preferred, since we observed a significantly lower labelling efficiency of ~30% using the weaker base K_2_C_2_O_4_. Our attempt to combine microwave dielectric heating with DMF as solvent with a mean-to-high dielectric constant rendered comparably high labeling efficiencies (≈94%) under microwave pulse mode (power cycling mode, 150 °C, 75 W, 5 min).

A semi-automated radiosynthesis coupled with microwave was recently reported for [^18^F]**10b** ([^18^F]ASEM) using the DMSO/K_2_CO_3_ system at 160 °C, resulting in a labeling efficiency of 45%–50% [54]. In the attempt to gain insight into the influence of the solvent on labeling efficiencies, we radiolabeled the nitro precursor **11b** to obtain [^18^F]**10b** using the DMF/K_2_CO_3_ system under conventional and microwave-assisted heating at 120 °C. With only 1 mg of the nitro precursor **11b**, labeling efficiencies of 60%–70% were achieved with DMF as solvent in both heating modes. These findings reinforce the superiority of DMF as a solvent of choice for this compound class, and allows the fully automated radiosynthesis of [^18^F]**10a** and [^18^F]**10b** for transfer to clinical radiopharmacies.

**Figure 2:**
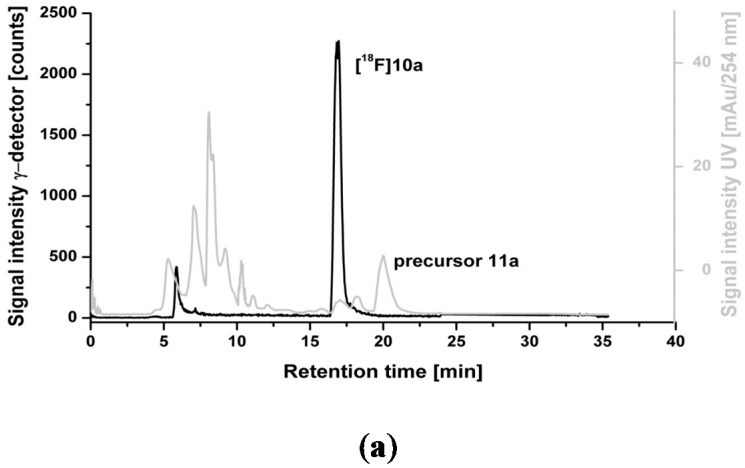

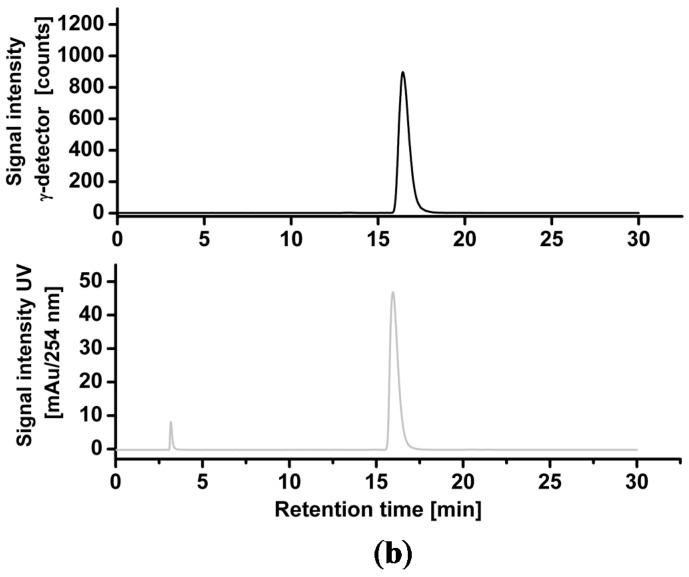
(**a**) Representative radio- and UV-chromatograms obtained for isolation of [^18^F]**10a** from crude reaction mixture by semi-preparative HPLC (Reprosil-Pur C18-AQ column, 35% MeCN/H_2_O/0.05% TFA, Flow rate: 10 mL∙min^−1^); **(b**) Analytical radio-chromatogram (top) and UV-chromatogram (bottom) of purified [^18^F]**10a** spiked with the reference compound **10a**.

Overall, the radiolabelling reactions proceeded cleanly. No significant amount of ^18^F-labeled by-products was detected according to radio-TLC analyses (data not shown). The crude reaction mixture was applied directly onto a semi-preparative HPLC column (Figure 2a) and [^18^F]**10a** was successfully isolated in high radiochemical purities (≥98%) with a retention time of about 17 min. Analytical radio-HPLC analysis of the final product spiked with the reference compound confirmed the identity of [^18^F]**10a** (Figure 2b). [^18^F]**10a** proved to be stable in physiological solutions and organic solvents at 40 °C for up to 90 min.

We determined the distribution coefficient of [^18^F]**10a** in the *n*-octanol-PBS system experimentally by the shake-flask method with a log *D*_7.2_ value of 1.3 ± 0.1 (*n* = 3), making sufficient blood-brain barrier permeability of [^18^F]**10a** likely.

The translation to remotely controlled radiosynthesis of [^18^F]**10a** was performed using the TRACERLAB™ FX_FN_ module. The reaction was performed using the Kryptofix^®^222/K_2_CO_3_ system at 120 °C in 10 min with 1 mg of precursor **11a** in DMF. Also in the automated process [^18^F]**10a** was obtained with extremely high labeling efficiencies of 86% ± 3%. After isolation via semi-preparative HPLC, [^18^F]**10a** was trapped on a pre-conditioned Sep-Pak^®^ (Waters, Milford, MA, USA) C18 light cartridge, and formulated in isotonic saline containing 10% of EtOH (*v*/*v*). The average decay-corrected radiochemical yield was 35% ± 9% (*n* = 6) calculated at the end of the synthesis (EOS). Radiochemical purity of >99% and high specific activities of 855 ± 302 GBq·μmol^−1^ (*n* = 6) were obtained in a total synthesis time of 70 min. This rapid and versatile automated radiosynthesis will enhance the accessibility of [^18^F]**10a** for widespread production in future clinical studies.

### 2.4. Dynamic PET Studies of [^18^F]**10a** in Piglets

#### 2.4.1. Baseline Studies

**Figure 3:**
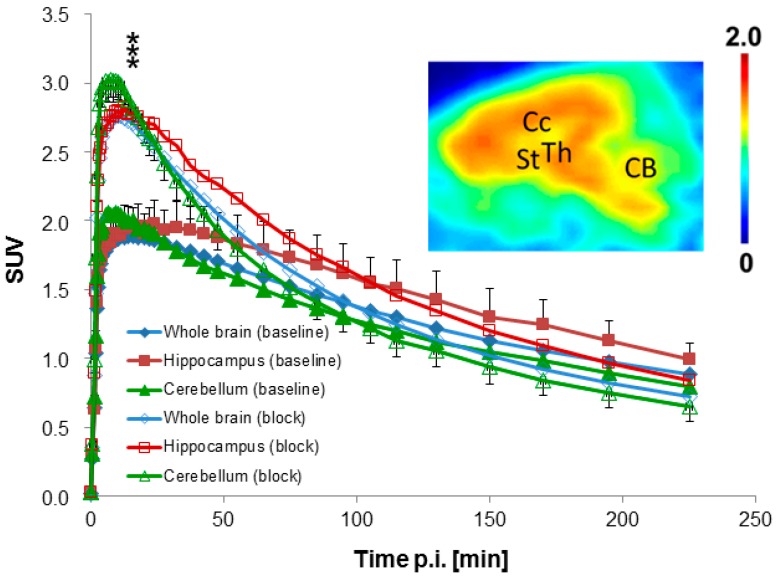
Time-activity curves in different brain regions obtained by dynamic PET scans of [^18^F]**10a** in piglets under baseline and blocking conditions (mean values; *n* = 3). Standard deviations are shown for two examples (hippocampus baseline and cerebellum block). *** *p* < 0.001 *vs.* baseline. Inset: Brain image in sagittal plane acquired from 0 to 20 min p.i. after injection of [^18^F]**10a** in a female piglet. Data are expressed as standardized uptake value (SUV). Regions of interest (ROI) were drawn based on an overlay with T1-weighted MR images of a pig brain. Abbreviations: CB = Cerebellum, CC = Corpus callosum, St = Striatum, Th = Thalamus.

After intravenous injection of 425 ± 78 MBq [^18^F]**10a** (*n* = 3), we observed high uptake of radioactivity in the brain with peak concentrations at 8–10 min p.i., followed by washout. The highest accumulation of radioactivity (SUV_max_ > 2.2) occurred in the thalamus, colliculi, and midbrain. Somewhat lower accumulation (SUV_max_ ~1.9–2.1) was observed in other brain regions (Figure 3). The summed PET image (inset of Figure 3) shows the corresponding distribution pattern in the brain of a female piglet from 0 to 20 min p.i.

#### 2.4.2. Metabolite Analysis

**Figure 4:**
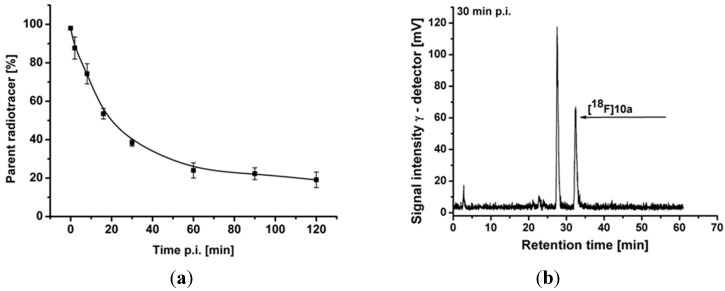
(**a**) Tri-exponential function fitting of the percentage of [^18^F]**10a** under baseline conditions (*n* = 3) which was used to obtain metabolite-corrected arterial plasma input functions for modelling; (**b**) Representative radio-chromatogram of a pig plasma sample obtained at 30 min after intravenous injection of [^18^F]**10a** under baseline conditions.

Radioactive metabolites of [^18^F]**10a** were assessed in plasma of pigs for up to 120 min p.i. (Figure 4a). For preparation of RP-HPLC samples, the proteins were precipitated and extracted two times with MeCN with a reproducible recovery of >90% of the starting radioactivity in the supernatant. The patterns of radioactive metabolites obtained by radio-TLC and radio-HPLC correlated well with each other. Parent fraction accounted for 88% ± 6%, 24% ± 4%, and 19% ± 4% of total radioactivity at 2, 60, and 120 min p.i., respectively.

One major radioactive metabolite was detected, which was more hydrophilic than [^18^F]**10a** and is therefore assumed not to pass the blood-brain barrier (Figure 4b). Based on previous experience from our group with the diazabicyclononane-containing α_7_ nAChR ligand [^18^F]NS10743 [21], we believe that this metabolite is formed by enzymatic oxidation of the nitrogen at position 1 in the identical motif of **10a**.

#### 2.4.3. Blocking Studies

To evaluate the binding specificity of [^18^F]**10a** *in* *vivo*, we performed blocking studies (*n* = 3). Animals were pretreated with 3 mg∙kg^−1^ of the α_7_ nAChR ligand NS6740 [3] at 10 min prior to injection of 429 ± 45 MBq [^18^F]**10a**, followed by a continuous infusion of NS6740 at 1 mg∙kg^−1^∙h^−1^ during the course of the PET scan [21].

Despite a significant increase of about 40%–50%, in the brain uptake, radioactivity uptake under blocking conditions was about 15%–20% lower than that under baseline conditions in all brain regions evaluated from about 90 min p.i. until the end of the study (Figure 3). The early increase of brain uptake is accompanied by a significant increase of the influx rate constant *K*_1_ (Table 4). The most likely explanation is a blood flow driven effect. There is strong evidence that α_7_ nAChRs are expressed on vascular endothelial and smooth muscle cells [55,56,57], which we assume were activated by NS6740 despite its classification as a “weak agonist” [3].

#### 2.4.4. Modeling

One-tissue and two-tissue compartment models (1TCM and 2TCM) were evaluated for mathematical description of [^18^F]**10a** time-activity curves (TACs) in pig brain regions (Figure 3) using metabolite-corrected arterial plasma input functions. The 2TCM was identified as the more appropriate model based on its smaller Akaike information criterion (AIC) value. The four rate constants *K*_1_, *k*_2_, *k*_3_, and *k*_4_ were fitted and the non-displaceable volume of distribution (*V*_ND_ = *K*_1_/*k*_2_) and the binding potential (*BP*_ND_ = *k*_3_/*k*_4_) as defined by Innis *et al.* [58] were calculated for each region of interest (Table 3 and Table 4).

The values of the influx rate constant *K*_1_ and the clearance rate constant *k*_2_ were comparable between the different brain regions with a mean of 0.362 mL·cm^−3^·min^−1^ and 0.131 mL·cm^−3^·min^−1^, respectively, in the whole brain. The total volume of distribution *V*_T_ calculated from *K*_1_/*k*_2_(1 + *k*_3_/*k*_4_) [58] was 17 mL∙cm^−3^ in thalamus and 13 mL∙cm^−3^ in cerebellum, *i.e.*, slightly lower than the comparable values for [^18^F]ASEM in baboons [18].

**Table 3:** Kinetic rate constants, non-displaceable volume of distribution (*V*_ND_), and non-displaceable binding potential (*BP*_ND_) of [^18^F]**10a** in different regions of piglet brain using 240 min of scan data under baseline conditions (*n* = 3).

Brain Region	Rate Constant				*V*_ND_	*BP*_ND_
	*K*_1_ (mL·cm^−3^·min^−1^)	*k*_2_ (min^−1^)	*k*_3_ (min^−1^)	*k*_4_ (min^−1^)		
Whole	0.362 ± 0.038	0.131 ± 0.062	0.093 ± 0.074	0.027 ± 0.018	3.32 ± 1.74	3.31 ± 1.02
Frontal Cortex	0.387 ± 0.059	0.182 ± 0.153	0.142 ± 0.165	0.027 ± 0.015	3.32 ± 2.06	4.22 ± 2.93
Parietal Cortex	0.399 ± 0.038	0.140 ± 0.089	0.126 ± 0.135	0.031 ± 0.021	3.88 ± 2.49	3.47 ± 1.65
Occipital Cortex	0.395 ± 0.053	0.123 ± 0.063	0.099 ± 0.087	0.030 ± 0.023	4.07 ± 2.23	3.16 ± 1.11
Hippocampus	0.400 ± 0.023	0.188 ± 0.083	0.145 ± 0.101	0.025 ± 0.013	2.43 ± 0.96	5.50 ± 1.01
Striatum	0.382 ± 0.028	0.144 ± 0.079	0.114 ± 0.093	0.027 ± 0.014	3.35 ± 1.81	3.98 ± 1.40
Thalamus	0.448 ± 0.027	0.150 ± 0.071	0.111 ± 0.085	0.030 ± 0.018	3.67 ± 2.10	3.51 ± 1.13
Colliculi	0.450 ± 0.072	0.134 ± 0.074	0.070 ± 0.042	0.027 ± 0.018	4.19 ± 2.37	2.68 ± 1.42
Midbrain	0.436 ± 0.045	0.162 ± 0.117	0.121 ± 0.132	0.029 ± 0.022	4.04 ± 3.06	3.40 ± 1.73
Pons	0.440 ± 0.045	0.149 ± 0.059	0.068 ± 0.042	0.026 ± 0.019	3.32 ± 1.40	2.76 ± 0.81
Cerebellum	0.421 ± 0.048	0.131 ± 0.052	0.067 ± 0.051	0.026 ± 0.020	3.62 ± 1.47	2.53 ± 0.57

**Table 4:** Kinetic rate constants, non-displaceable volume of distribution (*V*_ND_), and non-displaceable binding potential (*BP*_ND_) of [^18^F]**10a** in different regions of piglet brain using 240 min of scan data under blocking conditions (*n* = 3).

Brain Region	Rate Constant				*V*_ND_	*BP*_ND_
	*K*_1_ (mL·cm^−3^·min^−1^)	*k*_2_ (min^−1^)	*k*_3_ (min^−1^)	*k*_4_ (min^−1^)		
Whole	0.598 ± 0.108 *	0.062 ± 0.015	0.018 ± 0.022	0.011 ± 0.018	10.27 ± 3.94 *	0.84 ± 1.07 *
Frontal Cortex	0.647 ± 0.117 *	0.055 ± 0.011	0.014 ± 0.019	0.010 ± 0.020	12.34 ± 4.27 *	0.86 ± 0.92
Parietal Cortex	0.659 ± 0.120 *	0.055 ± 0.013	0.015 ± 0.020	0.011 ± 0.021	12.58 ± 4.52 *	0.82 ± 0.88 *
Occipital Cortex	0.668 ± 0.103 *	0.064 ± 0.019	0.022 ± 0.032	0.013 ± 0.023	11.36 ± 4.41 *	0.95 ± 1.03 *
Hippocampus	0.603 ± 0.118 *	0.063 ± 0.023 *	0.024 ± 0.030	0.011 ± 0.015	10.81 ± 5.02 *	0.84 ± 1.70 *
Striatum	0.624 ± 0.143 *	0.057 ± 0.015	0.020 ± 0.025	0.011 ± 0.017	11.78 ± 4.91 *	0.75 ± 1.29 *
Thalamus	0.762 ± 0.129 *	0.070 ± 0.025	0.023 ± 0.031	0.012 ± 0.021	12.09 ± 5.31 *	0.88 ± 1.00 *
Colliculi	0.735 ± 0.182 *	0.066 ± 0.007	0.011 ± 0.013	0.007 ± 0.015	11.41 ± 3.90 *	0.73 ± 0.74 *
Midbrain	0.685 ± 0.131 *	0.063 ± 0.010	0.013 ± 0.014	0.012 ± 0.018	11.17 ± 3.64 *	0.61 ± 0.77 *
Pons	0.670 ± 0.092 *	0.084 ± 0.030	0.019 ± 0.023	0.012 ± 0.016	8.93 ± 3.96 *	0.76 ± 1.16 *
Cerebellum	0.711 ± 0.148 *	0.076 ± 0.014	0.016 ± 0.019	0.011 ± 0.016	9.75 ± 3.57 *	0.71 ± 1.10 *

* *p* < 0.05 *vs.* baseline (Table 3).

Under blocking conditions *K*_1_ was significantly increased by 50% to 70% in all brain regions while *k*_2_ was decreased between 42% and 70% resulting in a significant (up to 4-fold) increase of *V*_ND_ (Table 4) and preventing the use of the occupancy plot [59] for calculation of *BP*_ND_. Therefore *BP*_ND_, as the ratio (at equilibrium) of specifically bound radioligand to that of nondisplaceable radioligand in tissue [58], was directly calculated form the rate constants *k*_3,_ which is proportional to the association rate constant, and *k*_4_, which is proportional to the dissociation rate constant from the specific compartment. The baseline study revealed that the rate constant *k*_3_ was highest in the hippocampus and lowest in the cerebellum and the values of *k*_4_ were low, rather uniform and ranged from 0.025 to 0.031 min^−1^. *BP*_ND_ values between 5.5 (hippocampus) and 2.5 (cerebellum) were reliably calculated from these data (SD_whole_ _brain_ ~30%). A similarly high *BP*_ND_ has recently been reported in baboons for the structurally related [^18^F]ASEM [18] but not for any previous α_7_ nAChR PET radioligands [60]. Under blocking conditions *k*_3_ was decreased by 70% to 90% in various brain regions while *k*_4_ was decreased between 60% and 70%, resulting in a significant decrease of *BP*_ND_ by 72% to 85% in all regions but the frontal cortex (Table 4), suggesting specific binding of [^18^F]**10a** to α_7_ nAChR in a similar range as the recently reported [^18^F]ASEM [18].

### 2.5. Comparative PET Study of *[^18^F]**10a*** ([^18^F]DBT-10) and *[^18^F]**10b*** ([^18^F]ASEM) in a Rhesus Monkey

**Figure 5:**
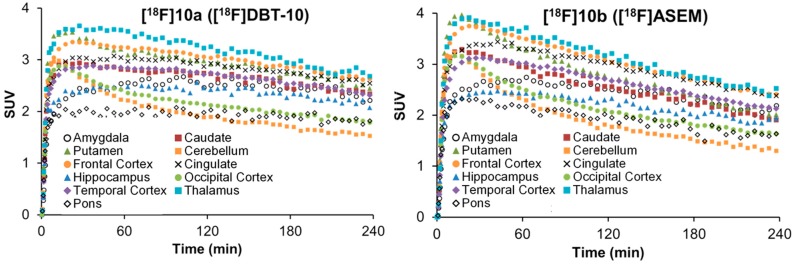
Time-activity curves of [^18^F]DBT-10 and [^18^F]ASEM in selected brain regions of a rhesus monkey.

A PET study in a single rhesus monkey was performed for direct comparison of [^18^F]DBT-10 (injected dose: 167 MBq) and [^18^F]ASEM (injected dose: 185 MBq). Regional time-activity curves for both radioligands in the monkey brain are presented in Figure 5. Initial uptake levels were lower, and kinetics slower for [^18^F]DBT-10 than [^18^F]ASEM. For both radioligands the uptake levels follow the order of thalamus > frontal cortex = putamen > caudate > hippocampus > occipital cortex > cerebellum.

The total volume of distribution *V*_T_ for both radioligands was similar with the highest values in the thalamus ([^18^F]DBT-10: 48.1 mL∙cm^−3^; [^18^F]ASEM: 46.7 mL∙cm^−3^) and the lowest in the cerebellum ([^18^F]DBT-10: 28.5 mL∙cm^−3^; [^18^F]ASEM: 26.8 mL∙cm^−3^). These values are almost two-fold higher than those reported for [^18^F]ASEM in baboons [18], supporting the species differences in α_7_ nAChR binding and distribution.

Altogether these preliminary data support the equal potency of both [^18^F]DBT-10 and [^18^F]ASEM for PET imaging of α_7_ nAChRs, with [^18^F]ASEM having slightly faster kinetics.

### 2.6. Toxicity Studies in Rats

To prepare [^18^F]**10a** for use in humans, toxicity studies were performed in rats according to EU cGLP. Compound **10a** was administered by a single intravenous injection to rats followed by an observation period of 2 or 15 day. The study was performed with 4 test groups, including 1 control and 3 dose groups (6.2, 62 and 620 μg∙kg^−1^), with 60 male and 60 female Wistar rats divided into two experiments: Day 2 (40 males and 40 females) and Day 15 (20 males and 20 females). During clinical observation the animals displayed no notable clinical effects. No statistically significant differences in body weights between control and treated groups in either gender were detected. Food consumption corresponded with body weight development.

All haematology parameters on Day 2 and Day 15 were within physiological range for this species. Individual divergences of some haematology parameters were small and not correlated with treatment. No test item effect on haematology parameters was observed.

There were no findings in clinical chemistry parameters which could be definitively considered as adverse. The average values of all test groups were within the historical control ranges. Occasional changes had no dose relationship, and they were therefore considered as a result of intra- and inter-individual variability for this species. The results of pathology examination indicated that **10a** after single intravenous administration did not result in changes in pathological and histopathological parameters on either Day 2 or Day 15. The no observed effect level (NOEL) of **10a** was determined to be 620 μg∙kg^−1^.

## 3. Experimental Section

### 3.1. General Information

Analytical thin-layer chromatography (TLC) was performed with Macherey-Nagel precoated plastic sheets with fluorescent indicator UV_254_ (Polygram^®^ SIL G/UV_254_, Düren, Germany). Visualization of the spots was effected by illumination with an UV lamp (254 nm and 366 nm). Dry-column flash chromatography (DCFC) [61] was performed with vacuum on silica gel 60 (particle size 15–40 μm, Ref. 815650) from Macherey-Nagel (Macherey-Nagel GmbH & Co. KG, Düren, Germany). NMR spectra (^1^H, ^13^C, ^13^C-APT, ^19^F, COSY, HSQC, HMBC) were recorded with Varian spectrometer (Varian Mercury-300BB and Mercury-400BB; Agilent Technologies, Santa Clara, CA, USA). Chemical shifts are reported as δ (δ_H_, δ_C_, δ_F_) values. Coupling constants are reported in Hz. Multiplicity is defined by s (singlet), d (doublet), t (triplet), and combinations thereof; br (broad) and m (multiplet). ESI/Ion trap mass spectra (LRMS) were recorded with a Bruker Esquire 3000 plus instrument (Billerica, MA, USA). High resolution mass spectra were recorded on an FT-ICR APEX II spectrometer (Bruker Daltonics; Bruker Corporation, Billerica, MA, USA) using electrospray ionization (ESI) in positive ion mode. Melting points were determined on a Linström capillary apparatus (Wagner & Munz GmbH, Vienna, Austria) in open capillary tubes and are uncorrected. Reagents and solvents were purchased from commercial sources and used without further purification unless otherwise noted. Compounds **2**, **4a**, and **5a** were prepared starting from **1a** (dibenzo[*b*,*d*]thiophene; Acros Organics, Geel, Belgium) according to literature methods [30,31,32]. The preparation of intermediates **3**, **5b**, **8a**, **8b**, and the final compound **10** have been described previously, but with different synthetic routes or from starting materials not used in our protocols [17,31,62]. Intermediate **6a** (two steps from **4a**), **6b** (one step from **5b**) and final compounds **10a**, **10b**, **11a** and **11b** (one step each, from amine **9a**), which have been prepared according to the literature [62], were also reported by Gao *et al.* [17]. To the best of our knowledge, intermediates **7a**, **8c**, **8d** and final compounds **10c**, **12a**,**b**, **13b**, and **14b** have not been reported so far.

### 3.2. Chemistry

*4-Nitrosodibenzo[b,d]thiophene* (**4**). *tert*-Butyl nitrite (2.14 mL, 1.86 g, 18 mmol) was added in one portion to a stirred suspension of dibenzo[*b*,*d*]thiophen-4-yl boronic acid (**1b**, 1.37 g, 6 mmol; Frontier Scientific, Logan, UT, USA) in MeCN (24 mL) under argon at 22 °C. The mixture was stirred at 50–55 °C while its colour turned from yellow to dark brown in the course of 24 h. The solvent was evaporated and the residual solid was dissolved in CH_2_Cl_2_ (80 mL), washed with H_2_O (25 mL), dried (MgSO_4_) and evaporated to leave a dark greenish brown residue (1.38 g), which was used for the oxidation step. A sample (40 mg) was dissolved in cyclohexane/CH_2_Cl_2_ (2:3, *v*/*v*, 3 mL) and filtered through a short plug of silica gel (15–40 μm, 2 g). Subsequent elution with cyclohexane/CH_2_Cl_2_ (2:3, *v*/*v*, 50 mL) gave a green eluate which was evaporated to yield 30 mg of the title compound **4** (R_f_ = 0.56, heptane/EtOAc, 3:1) as a green powder, m.p. 113–115 °C (lit. m.p. 115–117 °C [36]). ^1^H-NMR (300 MHz, CDCl_3_): δ 7.48–7.63 (m, 2H, 7-H, 8-H), 7.88–7.94 (m, 1H, 6-H), 7.94 (t, *J* = 7.7 Hz, 1H, 2-H), 8.13–8.22 (m, 1H, 9-H), 8.49 (dd, *J* = 7.7, 1.1 Hz, 1H, 3-H), 9.58 (dd, *J* = 7.6, 1.1 Hz, 1H, 1-H). ^13^C-NMR (75 MHz, CDCl_3_): δ 120.10 (C), 121.68 (CH), 123.67 (CH), 125.44 (CH), 125.84 (CH), 127.82 (CH), 128.21 (CH), 132.38 (C), 137.94 (C), 138.11 (C), 142.04 (C), 161.91 (C), 165.95 (C). The product is 88% pure, containing 12% of 4-nitrodibenzo[*b*,*d*]thiophene (**4b**), as determined by comparison with ^1^H-NMR of a pure sample.

*2-Nitrodibenzo[b,d]thiophene 5,5-dioxide* (**5a**) [32]. A mixture of 2-nitrodibenzo*[b,d]*thiophene (**4a**, 2.3 g, 10 mmol) in acetic acid (64 mL) was stirred at 80 °C while H_2_O_2_ (50%, 6.8 mL, 0.12 mol) was added. The temperature was raised to 120 °C and after 1 h a second portion H_2_O_2_ (50%, 4.5 mL, 0.08 mol) was added. Stirring was continued at 120 °C for 1 h and at 80 °C for 3 h. After cooling, the mixture was poured into water (180 mL) to form a precipitate which was filtered, washed and dried to yield the pure title compound **5a** (2.52 g, 9.64 mmol, 96% yield, R_f_ = 0.22, cyclohexane/CHCl_3_, 1:3) as a pale yellow powder, m.p. 256.5–257.5 °C (lit. m.p. 258 °C [32]); ^1^H-NMR (400 MHz, DMSO-*d*_6_) δ_H_ 7.74 (td, *J* = 7.6, 0.9 Hz, 1H_Ar_, 7-H), 7.87 (td, *J* = 7.6, 1.0 Hz, 1H_Ar_, 8-H), 8.07 (d, *J* = 7.7 Hz, 1H_Ar_, 6-H), 8.29 (m, 1H_Ar_, 9-H), 8.42 (dd, *J* = 8.4, 2.0 Hz, 1H_Ar_, 3-H), 8.48 (d, *J* = 7.8 Hz, 1H_Ar_, 4-H), 9.04 (d, *J* = 2.0 Hz, 1H_Ar_, 1-H). ^13^C-NMR (100 MHz, DMSO-*d*_6_): δ_C_ 118.26 (CH), 122.28 (CH), 123.60 (CH), 123.95 (CH), 126.10 (CH), 129.10 (C), 132.09 (CH), 132.98 (C), 135.04 (CH), 137.08 (C), 141.44 (C), 151.72 (C). LRMS: *m*/*z* (ESI) = 284.0 (M + Na)^+^.

*4-Nitrodibenzo[b,d]thiophene 5,5-dioxide* (**5b**). In a procedure similar to the preparation of **5a**, compound **4** (1.33 g, 5.8 mmol) was reacted with H_2_O_2_ (6.6 mL, 0.11 g, 116 mmol) in acetic acid to give 1.33 g of a dark yellow solid. The product was further purified by column chromatogaphy (silica gel 15–40 μm, 24 g) with cyclohexane/CH_2_Cl_2_ (1:1→1:2) to yield the pure title compound **5b** (1.1 g, 4.2 mmol, 72% yield, R_f_ = 0.2, CHCl_3_) as pale yellow crystals, m.p. 245.5–251 °C. ^1^H-NMR (400 MHz, DMSO-*d*_6_): δ_H_ 7.75 (td, *J* = 7.6, 0.8 Hz, 1H_Ar_, 7-H), 7.86 (td, *J* = 7.6, 1.0 Hz, 1H_Ar_, 8-H), 8.04 (d, *J* = 7.7 Hz, 1H_Ar_), 8.08 (t, *J* = 8.0 Hz, 1H_Ar_), 8.29 (d, *J* = 7.7 Hz, 1H_Ar_), 8.39 (dd, *J* = 8.2, 0.7 Hz, 1H_Ar_), 8.65 (dd, *J* = 7.7, 0.7 Hz, 1H_Ar_). ^13^C-NMR (100 MHz, DMSO-*d*_6_): δ_C_ 122.19 (CH), 123.21 (CH), 125.91 (CH), 128.29 (C), 129.16 (CH), 130.58 (C), 132.18 (CH), 134.45 (C), 134.71 (CH), 136.21 (CH), 137.26 (C), 143.39 (C, 4-C). LRMS: *m*/*z* (ESI) = 284.0 (M + Na)^+^.

*7-Bromo-2-nitrodibenzo[b,d]thiophene 5,5-dioxide* (**6a**). Compound **5a** (1.75 g, 6.7 mmol) was dissolved in H_2_SO_4_ (96%, 30 mL). *N*-Bromosuccinimide (1.28 g, 7.2 mmol) was added to this solution in several portions and the mixture was stirred at room temperature for 24 h. The suspension was poured into ice-water (90 mL) and stirred for 10 min. The precipitate was filtered off, washed with H_2_O until neutral, followed by MeOH, and dried at 60 °C to obtain a yellowish solid (2.29 g). The raw material was recrystallized twice from MeCN to afford the pure title compound **6a** (1.45 g, 4.27 mmol, 63.8% yield, R_f_ = 0.30, cyclohexane/CHCl_3_, 1:4) as a pale yellow powder, m.p. 281.0–282.5 °C. ^1^H-NMR (400 MHz, DMSO-*d*_6_): δ_H_ 8.08 (dd, *J* = 8.4, 1.8 Hz, 1H_Ar_, 8-H), 8.31 (d, *J* = 8.4 Hz, 1H_Ar_, 4-H), 8.41–8.47 (m, 3H_Ar_, 9-H, 3-H, 6-H), 9.07 (d, *J* = 1.8 Hz, 1H_Ar_, 1-H).^13^C-NMR (100 MHz, DMSO-*d*_6_): δ_C_ 118.52 (CH), 123.72 (CH), 125.11 (C), 125.36 (CH), 125.85 (CH), 126.35 (CH), 128.41 (C), 132.19 (C), 137.90 (CH), 138.66 (C), 141.11 (C), 151.78 (C). LRMS: *m*/*z* (ESI) = 364.0, 362.0 (M + Na)^+^.

*7-Bromo-2-fluorodibenzo[b,d]thiophene 5,5-dioxide* (**8a**). TMAF tetrahydrate (0.22 g, 1.3 mmol, Acros) was dried under an atmosphere of argon by azeotropic distillation with a mixture of DMSO (6 mL) and cyclohexane (12 mL) using a water separator for 6 h (bath temperature: 115–120 °C). The bath temperature was allowed to cool to 80 °C and the nitro derivative **6a** (0.34 g, 1 mmol) was added under stirring to the suspension of the dried TMAF in one portion. The mixture immediately turned dark brown and was stirred for additional 5 h at 95 °C. Reaction progress was monitored by TLC (cyclohexane/CHCl_3_, 1:4). The portion of cyclohexane was evaporated. The residual oil was poured into water (60 mL) and extracted with CH_2_Cl_2_ (4 × 15mL). 

The extracts were combined and washed with water (10 mL), dried (MgSO_4_) and evaporated. The residual yellow solid (0.283 g) was purified by column chromatography (silica gel 15–40 μm, 6 g) with CH_2_Cl_2_ to afford the pure title compound **8a**, (0.23 g, 0.74 mmol, 73.7% yield, R_f_ = 0.39, cyclohexane/CHCl_3_, 1:4) as a colorless powder, m.p. 266.5–268.5 °C. ^1^H-NMR (300 MHz, DMSO-*d*_6_): δ_H_ 7.52 (dt-like, *J* = 8.8, 8.7, 2.4 Hz, 1H_Ar_, 3-H), 8.05 (dd, *J* = 8.3, 1.8 Hz, 1H_Ar_, 8-H), 8.11 (dd, *J* = 8.6, 4.9 Hz, 1H_Ar_, 4-H), 8.16 (d, *J* = 8.3 Hz, 1H_Ar_, 9-H), 8.18 (dd, *J* = 9.2, 2.3 Hz, 1H_Ar_, 1-H), 8.35 (d, *J* = 1.6 Hz, 1H_Ar_, 6-H); ^13^C-NMR (75 MHz, DMSO-*d*_6_): δ_C_ 110.74 (d, ^2^*J*_CF_ = 25.6 Hz, CH), 118.22 (d, ^2^*J*_CF_ = 24.1 Hz, CH), 124.53 (s, C), 124.89 (d, ^3^*J*_CF_ = 10.2 Hz, CH), 125.09 (s, CH), 125.16 (s, CH), 128.98 (d, ^4^*J*_CF_ = 2.4 Hz, C), 132.82 (d, ^4^*J*_CF_ = 3.0 Hz, C), 133.45 (d, ^3^*J*_CF_ = 10.7 Hz, C), 137.49 (s, CH), 139.31 (s, C), 165.85 (d, ^1^*J*_CF_ = 252.2 Hz, C); ^19^F-NMR (282.36 MHz, DMSO-*d*_6_): δ_F_ −103.8 (m, F_Ar_, 2-F). LRMS: *m*/*z* (ESI) = 334.9, 336.9 (M + Na)^+^.

*2-Fluorodibenzo[b,d]thiophene 5,5-dioxide* (**7a**). Similar to the preparation of **8a**, TMAF tetrahydrate (1.35 g, 8.18 mmol) was azeotropically dried with cyclohexane/DMSO and subsequently reacted with **5a** (1.525 g, 5.84 mmol). After work-up, the yellow solid (1.25 g) was purified by column chromatography (silica gel 15–40 μm, 18 g) with cyclohexane/CH_2_Cl_2_ (1:1→1:3) as eluent to yield the pure title compound **7a** (1.03 g, 4.4 mmol, 75.2% yield, R_f_ = 0.4, cyclohexane/CHCl_3_, 1:4) as a colorless powder, m.p. 227–228 °C. ^1^H-NMR (400 MHz, CDCl_3_): δ_H_ 7.21 (dt, *J* = 8.45, 8.45, 2.27 Hz, 1H_Ar_, H-3), 7.45 (dd, *J* = 8.37, 2.26 Hz, 1H_Ar_, H-1), 7.57 (dt, *J* = 7.56, 7.53, 1.09 Hz, 1H_Ar_, H-7), 7.66 (dt, *J* = 7.65, 7.58, 1.17 Hz, 1H_Ar_, H-8), 7.75 (ddd, *J* = 7.70, 1.01, 0.57 Hz, 1H_Ar_, 9-H), 7.82 (dd, *J* = 8.42, 4.92 Hz, 1H_Ar_, H-4), 7.83 (ddd, *J* = 7.60, 1.09, 0.69 Hz, 1H_Ar_, 6-H); ^13^C-NMR (100 MHz, CDCl_3_): δ_C_ 109.34 (d, ^2^*J*_CF_ = 24.6 Hz, CH), 117.61 (d, ^2^*J*_CF_ = 23.9 Hz, CH), 121.98 (s, CH), 122.40 (s, CH), 124.58 (d, ^3^*J*_CF_ = 9.9 Hz, CH), 130.47 (d, ^4^*J*_CF_ = 2.5 Hz, C), 131.23 (s, CH), 133.72 (d, ^4^*J*_CF_ = 3.2 Hz, C), 134.13 (s, CH), 134.92 (d, ^3^*J*_CF_ = 9.7 Hz, C), 138.68 (s, C), 166.37 (d, ^1^*J*_CF_ = 255.0 Hz, C); ^19^F-NMR (282.36 MHz, DMSO-*d*_6_): δ_F_ ppm −103.63 (m. 1F, 2-F); LRMS: *m*/*z* (ESI) = 257.0, 258.0 (M + Na)^+^.

*3-Bromo-2-fluorodibenzo[b,d]thiophene 5,5-dioxide (**8c**) and 3,7-dibromo-2-fluoro-dibenzo[b,d]-thiophene 5,5-dioxide* (**8d**). Similar to the preparation of compound **6a**, compound **7a** (0.56 g, 2.4 mmol) was reacted with NBS (0.43 g, 2.4 mmol) in H_2_SO_4_. The solid residue obtained upon work-up (0.75 g) consisted of a mixture of starting material (R_f_ = 0.32), mono- (R_f_ = 0.43) and dibrominated derivative (R_f_ = 0.56) as shown by TLC analysis (cyclohexane/CHCl_3_, 1:4). The mixture was subjected to chromatographic purification (silica gel 15–40 μm, 28 g) with cyclohexane/CHCl_3_ (1:3) to yield the 2-fluoro-3,7-dibromo derivative **8d** (0.17 g, 0.43 mmol, 18%) from the first fraction and the 2-fluoro-3-bromo derivative **8c** (0.35 g, 1.12 mmol, 46.5%) from the second fraction. Compound **8c**: m.p. 269–274.5 °C. ^1^H-NMR (400 MHz, DMSO-*d*_6_): δ_H_ 7.72 (t, *J* = 7.6 Hz, 1H_Ar_, 7-H), 7.84 (t, *J* = 7.6 Hz, 1H_Ar_, 8-H), 8.01 (d, *J* = 7.7 Hz, 1H_Ar_, 6-H), 8.22 (d, *J* = 7.7 Hz, 1H_Ar_, 9-H), 8.32 (d, *J* = 8.9 Hz, 1H_Ar_, 1-H), 8.55 (d, *J* = 6.2 Hz, 1H_Ar_, 4-H). ^13^C-NMR (100 MHz, DMSO-*d*_6_): δ_C_ 110.78 (d, ^2^*J*_CF_ = 23.4 Hz, C), 111.57 (d, ^2^*J*_CF_ = 26.5 Hz, CH), 122.07 (s, CH), 123.33 (s, CH), 127.71 (d, ^3^*J*_CF_ = 1.5 Hz, CH), 129.16 (d, ^4^*J*_CF_ = 2.1 Hz, C), 131.80 (s, CH), 133.30 (d, ^3^*J*_CF_ = 9.9 Hz, C), 134.06 (d, ^4^*J*_CF_ = 3.5 Hz, C), 134.77 (s, CH), 137.28 (s, C), 162.00 (d, ^1^*J*_CF_ = 251.8 Hz, C). ^19^F-NMR (376.4 MHz, DMSO-*d*_6_): δ_F_ −98.68 (m, 1 F_Ar_, 2-F). LRMS: *m*/*z* (ESI) = 336.9, 335.0 (M + Na)^+^. Compound **8d**: m.p. 282.5–285 °C. ^1^H-NMR (300 MHz, CDCl_3_): δ_H_ 7.49 (d, *J* = 7.8 Hz, 1H_Ar_, 1-H), 7.60 (d, *J* = 8.2 Hz, 1H_Ar_, 9-H), 7.79 (dd, *J* = 8.2, 1.8 Hz, 1H_Ar_, 8-H), 7.94 (d, *J* = 1.7 Hz, 1H_Ar_, 6-H), 8.01 (d, *J* = 6.0 Hz, 1H_Ar_, 4-H). ^13^C-NMR (75 MHz, CDCl_3_): δ_C_ 110.07 (d, ^2^*J*_CF_ = 25.8 Hz, CH), 111.88 (d, ^2^*J*_CF_ = 12.2 Hz, C), 123.28 (s, CH), 125.53 (s, C), 125.84 (s, CH), 128.11 (d, ^3^*J*_CF_ = 1.9 Hz, CH), 128.75 (s, C), 132.68 (d, ^3^*J*_CF_ = 8.8 Hz, C), 134.32 (d, ^4^*J*_CF_ = 3.8 Hz, C), 137.41 (s, CH), 139.67 (s, C), 162.85 (d, *J* = 256.4 Hz, C). ^19^F-NMR (282.4 MHz, CDCl_3_) δ_F_ −98.68 (m, 1 F_Ar_, 2-F). LRMS: *m*/*z* (ESI) = 414.9, 416.9, 413.0 (M + Na)^+^.

*7-(1,4-Diazabicyclo[3.2.2]nonan-4-yl)-2-fluorodibenzo[b,d]thiophene 5,5-dioxide* (**10a**). A mixture of Pd_2_(dba)_3_ (11 mg, 0.012 mmol) and BINAP (15 mg, 0.024 mmol) in toluene (1.5 mL) was stirred for 30 min at 90 °C. The red-orange colored solution of the catalyst was allowed to cool (22 °C) and added to a mixture of 1,4-diazabicyclo[3.2.2]nonane (**9a**, 53 mg, 0.42 mmol) and **8a** (0.126 g, 0.40 mmol) in toluene (2 mL). Cs_2_CO_3_ (Alfa Aesar, Karlsruhe, Germany; previously dried for 2 h at 120 °C, 4 mbar; 0.39 g, 1.2 mmol) was then added, and the reaction mixture was stirred under an atmosphere of argon for 24 h at 90° C. After cooling to room temperature, the solid was filtered off and washed with CH_2_Cl_2_ (2 × 4 mL). The filtrate was evaporated and chromatographically purified (silica gel 15–40 μm, 8 g) with a gradient from CHCl_3_ (100%) to CHCl_3_/MeOH/NH_3_ (aq) (100:8:0.8). The fractions containing the product were combined, evaporated and the solid residue was recrystallized from EtOH to afford the title compound **10a** (0.075 g, 0.21 mmol, 52% yield, R_f_ = 0.18, CHCl_3_/MeOH/NH_3_ (aq.), 100:10:1) as yellow crystals, m.p. 317–319 °C (dec.). ^1^H-NMR (400 MHz, CDCl_3_): δ_H_ 1.78 (qd-like, *J* = 9.7, 4.6 Hz, 2H, 6′-H_a_, 9′-H_a_), 2.11 (m, 2H, 6′-H_b_, 9′-H_b_), 2.99 (m, 2H, 7′-H_a_, 8′-H_a_), 3.09 (A-part of AA′BB′, 2H, 2′-H_2_), 3.14 (m, 2H, 7′-H_b_, 8′-Hb_a_), 3.61 (B-part of AA′BB′, 2H, 3′-H_2_), 4.08 (m, not resolved, 1H, 5′-H),6.89 (dd, *J* = 8.8, 2.5 Hz, 1H_Ar_, 8-H), 7.00 (td-like, *J* = 8.5, 8.5, 2.2 Hz, 1H_Ar_, 3-H), 7.10 (d, *J* = 2.5 Hz,1H_Ar_,6-H), 7.24 (dd, *J* = 8.8, 2.2 Hz, 1H_Ar_, 1-H), 7.49 (d, *J* = 8.7 Hz, 1H_Ar_, 9-H), 7.70 (dd, *J* = 8.4, 4.9 Hz, 1H_Ar_, 4-H). ^13^C-NMR (100 MHz, CDCl_3_): δ_C_ 26.77 (s, 2 CH_2_), 44.69 (s, CH_2_), 46.51 (s, 2 CH_2_), 51.74 (s, CH), 57.05 (s, CH_2_), 105.19 (s, CH), 107.66 (d, ^2^*J*_CF_ = 25.1 Hz, CH), 114.70 (d, ^2^*J*_CF_ = 24.3 Hz, CH), 117.03 (s, CH), 117.28 (d, ^4^*J*_CF_ = 2.2 Hz, C), 123.15 (s, CH), 124.27 (d, ^3^*J*_CF_ = 10.3 Hz, CH), 132.88 (d, ^4^*J*_CF_ = 3.0 Hz, C), 136.19 (d, ^3^*J*_CF_ = 9.6 Hz, C), 140.61 (s, C), 151.15 (s, C), 166.58 (d, ^1^*J*_CF_ = 253.6 Hz, C). ^19^F-NMR (282.4 MHz, CDCl_3_): δ_F_ −104.5 (m, 1F_Ar_, 2-F). HRMS *m*/*z* (ESI): calcd for C_19_H_20_FN_2_O_2_S (M + H)^+^ 359.12240, found 359.12223.

*3-(1,4-Diazabicyclo[3.2.2]nonan-4-yl)-6-fluorodibenzo[b,d]thiophene 5,5-dioxide* (**10b**). In a procedure similar to the preparation of **10a**, compound **8b** (0.14 g, 0.45 mmol) and amine **9a** (0.059 g, 0.47 mmol) were reacted in the presence of Cs_2_CO_3_ (0.44 g, 1.35 mmol) and a catalyst made from Pd_2_(dba)_3_ (10.3 mg, 0.011 mmol) and BINAP (14 mg, 0.022 mmol). For purification the same protocol as for **10a** was followed to afford the title compound **10b** (0.077 g, 0.21 mmol, 48% yield, R_f_ = 0.19, CHCl_3_/MeOH/NH_3_ (aq), 100:10:1) as yellow crystals, m.p. 272.5–274 °C (dec.). ^1^H-NMR (300 MHz, CDCl_3_): δ_H_ 1.77 (qd-like, *J* = 9.7, 4.6 Hz, 2H, 6′-H_a_, 9′-H_a_), 2.11 (ddtd, *J* = 12.4, 9.7, 5.0, 2.6 Hz, 2H, 6′-H_b_, 9′-H_b_), 3.05–2.92 (m, 2H, 7′-H_a_, 8′-H_a_), 3.20–3.06 (m, A-part of AA′BB′, 4H, 7′-H_b_, 8′-Hb_a_, 3′-H_2_), 3.61 (t-like, *J* = 5.7 Hz, B-part of AA′BB′, 2H, 3′-H_2_), 4.08 (m, not resolved, 1H, 5′-H), 6.89 (dd, *J* = 8.8, 2.5 Hz, 1H_Ar_, 2-H), 6.97 (t, *J* = 8.4 Hz, 1H_Ar_, 7-H), 7.09 (d, *J* = 2.5 Hz, 1H_Ar_, 4-H), 7.35 (d, *J* = 7.6 Hz, 1H_Ar_, 9-H), 7.50 (dt, *J* = 8.0, 5.2 Hz, 1H_Ar_, 8-H), 7.53 (d, *J* = 8.7 Hz, 1H_Ar_, 1-H). ^13^C-NMR (75 MHz, CDCl_3_): δ_C_ 26.78 (s, 2 CH_2_), 44.70 (s, CH_2_), 46.52 (s, 2 CH_2_), 51.76 (s, CH), 57.06 (s, CH_2_), 105.09 (s, C_Ar_H), 115.17 (d, ^2^*J*_CF_ = 19.4 Hz, C_Ar_H), 115.85 (d, ^4^*J*_CF_ = 3.4 Hz, C_Ar_H), 117.09 (s, C_Ar_H), 117.65 (d, ^4^*J*_CF_ = 2.8 Hz, C_Ar_), 123.25 (s, C_Ar_H), 123.63 (d, ^2^*J*_CF_ = 17.7 Hz, C_Ar_), 135.83 (d, ^3^*J*_CF_ = 2.7 Hz, C_Ar_), 136.17 (d, ^3^*J*_CF_ = 7.9 Hz, C_Ar_H), 140.42 (s, C_Ar_), 151.12 (s, C_Ar_), 158.05 (d, ^1^*J*_CF_ = 257.4 Hz, C_Ar_). ^19^F-NMR (282.4 MHz, CDCl_3_): δ_F_ −115.66 (m, F_Ar_, 6-F). HRMS *m*/*z* (ESI): calcd for C_19_H_20_FN_2_O_2_S (M + H)^+^ 359.12240, found 359.12235.

*3-(1,4-Diazabicyclo[3.2.2]nonan-4-yl)-2-fluorodibenzo[b,d]thiophene 5,5-dioxide* (**10c**). In a procedure similar to the preparation of **10a**, compound **8c** (0.126 g, 0.4 mmol) and **9a** (0.053 g, 0.42 mmol) were reacted in the presence of Cs_2_CO_3_ (0.39 g, 1.2 mmol) and a catalyst mixture made from Pd_2_(dba)_3_ (11 mg, 0.012 mmol) and BINAP (14.9 mg, 0.024 mmol). For purification the same protocol for **10a** was followed to afford the title compound **10c** (0.063 g, 0.18 mmol, 44% yield, R_f_ = 0.20, CHCl_3_/MeOH/NH_3_ (aq), 100:10:1) as pale yellow crystals, m.p. 305–309 °C (dec.). ^1^H-NMR (300 MHz, CDCl_3_): δ_H_ 1.75 (m, 2H, 6′-H_a_, 9′-H_a_), 2.11 (m, 2H, 6′-H_b_, 9′-H_b_), 3.04 (m, 4H, 7′-H_2_, 8′-H_2_), 3.16 (t, *J* = 5.6 Hz, A-part of AA′BB′, 2H, 2′-H_2_), 3.44 (t, *J* = 5.6 Hz, B-part of AA′BB′, 2H, 3′-H_2_), 3.80 (m, 1H, 5′-H), 7.32 (s, 1H_Ar_, 4-H), 7.36 (d, *J* = 5.0 Hz, 1H_Ar_, 1-H), 7.43 (ddd, *J* = 7.7, 5.1, 3.4 Hz, 1H_Ar_, 7-H), 7.55–7.63 (m, 2H_Ar_, 8-H, 9-H), 7.75 (d, *J* = 7.6 Hz, 1H_Ar_, 6-H). ^13^C-NMR (75 MHz, CDCl_3_): δ_C_ 27.64 (s, 2 CH_2_), 46.70 (s, 2 CH_2_), 47.50 (d, ^4^*J*_CF_ = 1.2 Hz, CH_2_), 55.83 (d, ^4^*J*_CF_ = 6.0 Hz, CH), 56.30 (s, CH_2_), 109.97 (d, ^2^*J*_CF_ = 24.9 Hz, CH), 111.82 (d, ^3^*J*_CF_ = 5.5 Hz, CH), 120.89 (s, CH), 122.19 (s, CH), 123.72 (d, ^3^*J*_CF_ = 9.4 Hz, C), 129.25 (s, CH), 131.40 (d, ^4^*J*_CF_ = 2.3 Hz, C), 133.98 (d, ^4^*J*_CF_ = 2.9 Hz, C), 134.05 (s, CH), 137.87 (s, C), 142.90 (d, ^2^*J*_CF_ = 10.5 Hz, C), 157.62 (d, ^1^*J*_CF_ = 252.1 Hz, C). ^19^F-NMR (282.4 MHz, CDCl_3_): δ_F_ −112.95 (m, 1 F_Ar_, 2-F). HRMS *m*/*z* (ESI): calcd for C_19_H_20_FN_2_O_2_S (M+H)^+^ 359.12240, found 359.12235.

*6-Fluoro-3-(9-methyl-3,9-diazabicyclo[3.3.1]nonan-3-yl)dibenzo[b,d]thiophene 5,5-dioxide* (**12b**). In a procedure similar to the preparation of **10a**, compound **8b** (0.13 g, 0.42 mmol) and amine **9b** (0.065 g, 0.46 mmol) were reacted in the presence of Cs_2_CO_3_ (0.28 g, 0.84 mmol) and a catalyst made from Pd_2_(dba)_3_ (11.5 mg, 0.013 mmol) and BINAP (15.7 mg, 0.0252 mmol). For purification the same protocol for **10a** was followed to afford the title compound **12b** (0.065 g, 0.175 mmol, 42% yield, R_f_ = 0.31, CHCl_3_/MeOH/NH_3_ (aq), 100:10:1) as yellow crystals, m.p. 264–272 °C (dec.). ^1^H-NMR (300 MHz, CDCl_3_): δ_H_ 1.52–1.64 (m, 3H, 7′-H_a_, 6′-H_a_, 8′-H_a_), 1.93–2.15 (m, 3H, 7′-H_b_, 6′-H_b_, 8′-H_b_), 2.59 (s, 3H, 9′-NCH_3_), 3.01 (s, 2H, 1′-H, 5′-H), 3.39–3.51 (m, 4H, 2′-H_2_, 4′-H_2_), 6.97–7.03 (m, 2H_Ar_, 7-H, 2-H), 7.22 (d, *J* = 2.4 Hz, 1H_Ar_, 4-H), 7.39 (dd, *J* = 7.7, 0.5 Hz, 1H_Ar_, 9-H), 7.52 (ddd, *J* = 8.2, 7.8, 5.2 Hz, 1H_Ar_, 8-H), 7.59 (d, *J* = 8.7 Hz, 1H_Ar_, 1-H). ^13^C-NMR (75 MHz, CDCl_3_): δ_C_ 18.99 (s, 1 C_sec_), 27.93 (s, 2C_sec_), 40.62 (s, CH_3_), 47.40 (s, 2CH_2_), 53.03 (s, 2CH), 105.41 (s, C_Ar_H), 115.46 (d, ^2^*J*_CF_ = 19.4 Hz, C_Ar_H), 116.05 (d, ^4^*J*_CF_ = 3.4 Hz, C_Ar_H), 117.22 (s, C_Ar_H), 118.83 (d, ^4^*J*_CF_ = 2.6 Hz, C_Ar_), 122.99 (s, C_Ar_H), 123.77 (d, ^2^*J*_CF_ = 18.1 Hz, C_Ar_), 135.76 (d, ^3^*J*_CF_ = 2.5 Hz, C_Ar_), 136.20 (d, ^3^*J*_CF_ = 7.8 Hz, 1C_Ar_H), 140.10 (s, C_Ar_), 152.27 (s, C_Ar_), 158.06 (d, ^1^*J*_CF_ = 257.4 Hz, C_Ar_). ^19^F-NMR (282 MHz, CDCl_3_): δ −115.56 (dd-like, *J* = 8.4, 5.1 Hz, 1F, 6-F). HRMS *m*/*z* (ESI): calcd for C_20_H_21_FN_2_O_2_S (M + H)^+^ 373.13805, found 373.13773.

*7-(1,4-Diazabicyclo[3.2.2]nonan-4-yl)-2-nitrodibenzo[b,d]thiophene 5,5-dioxide* (**11a**). In a procedure similar to the preparation of **10a**, compound **6a** (0.17 g, 0.5 mmol) and amine **9a** (0.064 g, 0.51 mmol) were reacted in the presence of Cs_2_CO_3_ (0.49 g, 1.5 mmol) and a catalyst mixture made from Pd_2_(dba)_3_ (18.3 mg, 0.02 mmol) and BINAP (25 mg, 0.04 mmol). For purification the same protocol for **10a** was followed to afford the title compound **11a** (0.10 g, 0.18 mmol, 53% yield, R_f_ = 0.21, CHCl_3_/MeOH/NH_3_ (aq), 100:10:1) as dark red crystals, m.p. 291–294 °C (dec.). ^1^H-NMR (300 MHz, CDCl_3_): δ_H_ 1.79 (qd-like, *J* = 9.6, 4.6 Hz, 2H, 6′-H_a_, 9′-H_a_), 2.12 (m, 2H, 6′-H_b_, 9′-H_b_), 2.99 (m, 2H, 7′-H_a_, 8′-H_a_), 3.10 (A-part of AA′BB′, 2H, 2′-H_2_), 3.13 (m, 2H, 7′-H_b_, 8′-Hb_a_), 3.65 (B-part of AA′BB′, 2H, 3′-H_2_), 4.11 (m, not resolved, 1H, 5′-H), 6.94 (dd, *J* = 8.8, 2.6 Hz, 1H_Ar_, 8-H), 7.11 (d, *J* = 2.5 Hz, 1H_Ar_, 6-H), 7.63 (d, *J* = 8.8 Hz, 1H_Ar_, 9-H), 7.85 (d, *J* = 8.3 Hz, 1H_Ar_, 4-H), 8.15 (dd, *J* = 8.3, 2.0 Hz, 1H_Ar_, 3-H), 8.35 (d, *J* = 1.9 Hz, 1H_Ar_, 1-H). ^13^C-NMR (75 MHz, CDCl_3_): δ_C_ 26.70 (2 CH_2_), 44.71 (CH_2_), 46.47 (2 CH_2_), 51.72 (CH), 57.02 (CH_2_), 105.18 (CH), 115.16 (CH), 116.04 (C), 117.33 (CH), 122.59 (CH), 123.18 (CH), 123.67 (CH), 135.15 (C), 140.17 (C), 141.86 (C), 151.46 (C), 151.82 (C). HRMS *m*/*z* (ESI): calcd for C_19_H_20_N_3_O_4_S (M + H)^+^ 386.11690, found 386.11704.

### 3.3. Manual Radiosynthesis of *[^18^F]**10a***

No-carrier-added (n.c.a.) [^18^F]fluoride was produced via the ^18^O(p,n)^18^F nuclear reaction by irradiation of a [^18^O]H_2_O target (Hyox 18 enriched water; Rotem Industries Ltd, Mishor Yamin, Israel) on a Cyclone^®^18/9 (IBA RadioPharma Solutions, Louvain-la-Neuve, Belgium) with 18 MeV proton beam using a Nirta^®^ [^18^F]fluoride XL target or [^18^O]H_2_O recycled by the established in-house method [63]. Starting with 1–2 GBq of n.c.a. ^18^F-fluoride, [^18^F]F^−^-containing anion resin was eluted with a 20 mg mL^−1^ aqueous solution of K_2_CO_3_ (1.78 mg, 12.9 mmol) and added to a 5 mL V-vial in the Discover PET wave microwave CEM^®^ (CEM Corporation, Matthews, NC, USA) cavity in the presence of Kryptofix^®^222 (11.2 mg, 29.7 mmol) in 1 mL MeCN. The aqueous [^18^F]fluoride was dried under vacuum and argon flow in the microwave cavity (75 W, 20 cycles) at 50–60 °C for 10–12 min. Additional aliquots of MeCN (2 × 1.0 mL) were added for azeotropic drying. Thereafter, the precursor (1 mg) was added to the reactive anhydrous K[^18^F]F-Kryptofix^®^222/K_2_CO_3_-complex. The reaction parameters were optimized varying the reaction time, temperature, solvent (MeCN, DMF, DMSO), and heating condition (thermal *vs.* microwave-assisted). Thereafter, the crude reaction mixture was diluted with H_2_O/MeCN (4:1, *v*/*v*) and directly applied onto an isocratic semi-preparative HPLC for isolation of [^18^F]**10a**. Fractions were collected, diluted with 30 mL of H_2_O and sodium hydroxide was added to neutralize the solution (~100–200 μL of 1 M NaOH). Final purification was performed using a Sep-Pak^®^ C18 light cartridge (Waters, Milford, MA, USA) followed by elution with 1 mL of ethanol. To obtain an injectable solution the solvent was concentrated under a gentle argon stream and [^18^F]**10a** was formulated in a sterile isotonic saline solution (5%–10% EtOH, *v*/*v*). The identity of [^18^F]**10a** was verified by radio-HPLC analysis of a sample of the radiotracer solution spiked with the non-radioactive reference **10a**. Radiochemical and chemical purities were assessed by radio-TLC and analytical HPLC. The mass determination for specific activity was determined on the base of a calibration curve carried out under the same analytical HPLC conditions.

### 3.4. Automated Radiosynthesis of *[^18^F]**10a*** and *[^18^F]**10b*** ([^18^F]ASEM)

Remote controlled radiosynthesis was performed using a TRACERLAB™ FX_FN_ synthesizer (GE Healthcare, Waukesha, WI, USA) equipped with a PU-980 pump (JASCO, Gross-Umstadt, Germany), WellChrom K-2001 UV detector (KNAUER GmbH, Berlin, Germany), NaI(Tl)-counter and automated data acquisition (NINA software version 4.8 rev. 4; Nuclear Interface GmbH, Dortmund, Germany). For transfer to the automated module we started with activities in the range of 5–12 GBq of n.c.a. ^18^F-fluoride. Based upon the conditions optimized manually, the nitro-to-fluoro displacement was achieved by adding the respective nitro precursor (1 mg) dissolved in anhydrous DMF (1 mL) to the K[^18^F]F-Kryptofix^®^222/K_2_CO_3_-complex. The reaction mixture was stirred at 120 °C for 10 min. After cooling, the reaction mixture was diluted with 3 mL of H_2_O/MeCN (4:1) and directly applied onto the semi-preparative Reprosil-Pur 120 C18-AQ HPLC column (250 × 20 mm, 10 μm) using a solvent composition of 35% MeCN/H_2_O/0.05%TFA as eluent and a flow rate of 10 mL·min^−1^. The desired product was collected in 40 mL of H_2_O, neutralized with 1 M NaOH and directly transferred to a pre-activated Sep-Pak^®^ C18 light cartridge. The cartridge was washed with 2 mL of water and [^18^F]**10a** or [^18^F]**10b** was obtained after elution with 1 mL of EtOH. Injectable solutions were obtained by partial evaporation of the solvent under a gentle argon stream at 70 °C and dilution in isotonic saline (5%–10% of EtOH, *v*/*v*). Radiochemical purity and specific activity were assessed following the chromatographic methods described in quality control.

### 3.5. Quality Control

To control the quality of the K[^18^F]F-Kryptofix^®^222/K_2_CO_3_-complex, reactions using ethylene glycol ditosylate were performed randomly. Radio-TLC of [^18^F]**10a** and [^18^F]**10b** was performed on Polygram^®^ ALOX N/UV254 plates (Macherey-Nagel GmbH & Co. KG) with dichloromethane/methanol (9:1, *v*/*v*). The spots of the references were visualized using UV light at 254 nm. Radiochemical purity and specific activity were determined using analytical radio-HPLC with a Reprosil-Pur C18-AQ column (250 × 4.6 mm, 5 μm) and 26% MeCN/H_2_O/0.05% TFA as eluent at a flow rate of 1 mL·min^−1^. Analytical radio-HPLC profiles for the *in* *vivo* metabolism analysis in plasma was assessed by using a gradient mode (0–10 min: 10% MeCN/20 mM NH_4_OAc_aq._, 10–40 min: 10%→90% MeCN/20 mM NH_4_OAc_aq._, 40–50 min: 90%→10% MeCN/20 mM NH_4_OAc_aq._, 50–60 min: 10% MeCN/20 mM NH_4_OAc_aq._) on a Reprosil-Pur C18-AQ (250 × 4.6 mm, 5 μm) column at a flow rate of 1 mL·min^−1^.

### 3.6. Determination of in Vitro Stability and Lipophilicity (Log D_7.2_)

*in* *vitro* radiochemical stability of [^18^F]**10a** was investigated in 0.9% NaCl solution, PBS (pH 7.2), and 0.01 M Tris-HCl (pH 7.4 at 21 °C) at 40 °C and in EtOH at room temperature for up to 90 min. Samples were taken at 15, 30, 60, and 90 min of incubation time and analyzed by radio-TLC and radio-HPLC. Log D_7.2_ of [^18^F]**10a** was experimentally determined in n-octanol/phosphate-buffered saline (PBS; 0.01 M, pH 7.2) at room temperature by the shake-flask method in multiple distribution. Measurement was performed twice in triplicate.

### 3.7. Biological Evaluation

#### 3.7.1. Animals

Male Sprague-Dawley rats (275–300 g) were obtained from Charles River Laboratories, and female piglets (German Landrace pigs DL × Large White/Pietrain; mean weight 15.8 ± 0.8 kg, mean age 8 weeks) as well as female CD-1 mice (10–12 weeks, 22–26 g) were obtained from Medizinisch-Experimentelles Zentrum, Universität Leipzig, and all animals were provided with food and water ad libitum. All of the animal experiments were performed in accordance with the European Communities Council Directive of 24th November 1986 (86/609/EEC), and experiments using female piglets and female CD-1 mice were approved by the Animal Care and Use Committee of Saxony (TVV 08/13). Imaging experiments in rhesus monkeys were performed in accordance with a protocol approved by the Yale Institutional Animal Care and Use Committee.

#### 3.7.2. Binding Assays

##### Affinity for Human α_7_ nAChR

The affinity of the test compounds for human α_7_ nAChR was determined by radioligand displacement experiments as previously described [12]. In brief, SH-SY5Y cells stably expressing human α_7_ nAChR [64] were grown in DMEM/Ham’sF-12, supplemented with 10% FCS, stable glutamine, geneticin (100 μg∙mL^−1^), penicillin/streptomycin (100 U∙mL^−1^, 100 μg∙mL^−1^) at 37 °C in an atmosphere containing 5% CO_2_. Cells were harvested by scraping, sedimented (800 rpm, 3 min), diluted with 50 mM TRIS–HCl, pH 7.4, and stored at −25°C until use. For determination of α_7_ nAChR affinity of reference compounds, frozen cell suspensions were thawed, homogenized by a 27-gauge needle, and diluted with incubation buffer (50 mM TRIS–HCl, pH 7.4, 120 mM NaCl, 5 mM KCl). Membrane suspension was incubated with [^3^H]methyllycaconitine ([^3^H]MLA; ~0.3 nM final concentration; specific activity of 2.220 GBq∙mmol^−1^; NEN Life Sciences Products, Boston, MA, USA) and various concentrations of the test compound. Nonspecific binding was determined by co-incubation with 300 μM (−)-nicotine tartrate. The incubation was performed at room temperature for 120 min and terminated by rapid filtration using Whatman GF/B glass-fibre filters, presoaked in 0.3% polyethyleneimine, and a 48-channel harvester (Biomedical Research and Development Laboratories, Gaithersburg, MD, USA), followed by 4× washing with ice-cold 50 mM TRIS-HCl, pH 7.4. Filter-bound radioactivity was quantified by liquid scintillation counting. The 50% inhibition concentrations (IC_50_) were estimated from the competition curves by nonlinear regression using GraphPadPrism software and the *K*_i_ values calculated according to the Cheng-Prusoff equation [65].

##### Affinity for Human α_4_β_2_ and α_3_β_4_ nAChR

The affinity of the test compounds for human α_4_β_2_ and α_3_β_4_ nAChR was determined as described above. In brief, HEK293 cells stably expressing human α_4_β_2_ or α_3_β_4_ nAChR [66] were cultured. The cells were harvested and processed as described above, and membrane suspension was incubated with (±)-[^3^H]epibatidine (0.4 to 0.6 nM final concentration; specific activity of 1250 GBq∙mmol^−1^; PerkinElmer Human Health, Rodgau, Germany) and various concentrations of the test compound. Nonspecific binding was determined in the presence of 300 μM (−)-nicotine tartrate. Incubation of the samples, separation of free from receptor-bound radioligand, and data evaluation were performed as described above.

##### Affinity for Subunit-Specific Antibodies Immobilized Native Rat α_6_β_2_* nAChR

The specificity of the anti-α_6_ antibody was tested as described [67]. The receptors were prepared and immobilized as previously described [68] by the subunit-specific antibody. The inhibition of [^3^H]epibatidine binding to the immobilized subtypes by the test compounds was measured by pre-incubating increasing concentrations of the compounds for 1 h at room temperature, followed by incubation overnight at 4 °C with 0.1 nM (±)-[^3^H]epibatidine (specific activity of 1250 GBq∙mmol^−1^; PerkinElmer Human Health, Rodgau, Germany). Incubations were performed in a buffer containing 50 mM Tris-HCl, pH 7, 150 mM NaCl, 5 mM KCl, 1 mM MgCl_2_, 2.5 mM CaCl_2_, 2 mg∙mL^−1^ BSA, and 0.05% Tween 20. Specific ligand binding was defined as total binding minus the binding in the presence of 100 nM (±)-epibatidine dihydrochloride hydrate. After incubation, the wells were washed seven times with ice-cold PBS containing 0.05% Tween 20, the bound [^3^H]epibatidine released by adding 200 μL of 2 M NaOH to each well and after incubation for 2 h determined by means of liquid scintillation in a beta counter.

Saturation binding analysis revealed an affinity of (±)-[^3^H]epibatidine of 25 pM (CV 20%) for the nAChRs immobilized by anti-α_6_ antibody from rat striatum, which contains two major α_6_β_2_* subtypes (α_6_β_2_β_3_ and α_6_α_4_β_2_β_3_) in very similar proportions.

The experimental data obtained from the binding experiments were analyzed by means of a non-linear least square procedure using the LIGAND program [69]. The *K*_i_ values of the test compounds were also determined by means of the LIGAND program by simultaneously fitting the data obtained from three to five independent saturation and competition binding experiments.

##### Affinity for Human 5-HT_3_ Receptor

The affinity of the test compounds for human 5-HT_3_ receptor was evaluated by a contract research organization (Eurofins Panlabs, Inc., Taipei, Taiwan). All compounds were tested at 1 μM in radioligand binding assays using human recombinant HEK-293 cells for the percentage inhibition of the binding by the 5-HT_3_ specific radioligand [^3^H]GR-65630 (working concentration = 0.69 nM, *K*_D_ = 0.20 nM). In addition, for compound **10a** a *K*_i_ value was estimated based on the inhibition of the radioligand binding at seven concentrations of the test compound (20 μM–5 nM).

##### Affinity for Other Target Proteins

The activity of compound **10a** was evaluated by a contract research organization (Eurofins Panlabs, Inc.). The test concentration was 1 μM and radioligand binding assays for dopamine transporter (DAT), serotonin transporter (SERT), norepinephrine transporter (NET), monoamine transporter (VMAT), choline transporter, and α_1_ nAChR performed according to the manufacturer’s protocols. Responses were judged as significant at ≥50% inhibition or stimulation of the binding of the specific radioligand.

#### 3.7.3. PET Studies in Piglets

Six female animals were used in this study with anesthesia and surgery performed as described previously [70]. In brief, the animals received initially a premedication with midazolam (1 mg∙kg^−1^ i.m.) followed by induction of anesthesia with 3% isoflurane in 70% N_2_O/30% O_2_. All incision sites were infiltrated with 1% lidocaine, and the anesthesia was maintained with 1.5% isoflurane throughout the surgical procedure. A central venous catheter was introduced through the left external jugular vein and used for the administration of the radiotracer and drugs, and for volume substitution with heparinized (50 IE∙mL^−1^) lactated Ringer’s solution (2 mL∙kg^−1^∙h^−1^). An endotracheal tube was inserted by tracheotomy for artificial ventilation (Servo Ventilator 900C, Siemens-Elema, Solna, Sweden) after immobilization with pancuronium bromide (0.2 mg∙kg^−1^∙h^−1^). The artificial ventilation was adjusted to obtain normoxia and normocapnia (Radiometer ABL 500, Copenhagen, Denmark). Polyurethane catheters (i.d., 0.5 mm) were advanced through the left and the right femoral arteries into the abdominal aorta to withdraw arterial blood samples for regular monitoring of blood gases and for radiotracer input function measurements and metabolite analyses. The body temperature was monitored by a rectal temperature probe and maintained at ~38 °C by a heating pad. After the surgical procedure has been completed, anesthesia was maintained with 0.5% isoflurane in 70% N_2_O/30% O_2_, and the animals were allowed to stabilize for 1 h before imaging procedure.

##### PET Scanning Protocol

PET imaging was performed according to the protocol described recently [21]. In brief, a clinical tomograph (ECAT Exact HR+; Siemens Healthcare GmbH, Erlangen, Germany) was used with animals lying prone with the head placed in a custom-made head holder. For attenuation and scatter correction, transmission scans were acquired using three rotating ^68^Ge rod sources. The radiotracer was injected as an intravenous (i.v.) bolus (10 mL saline with 340–494 MBq [^18^F]**10a**) by a syringe pump within 2 min followed by flushing with 10 mL heparinized saline (50 IE∙mL^−1^). PET scanning started at the time of injection (0 min) and dynamic emission data were acquired for a total of 240 min. Three animals were investigated under baseline conditions, and another three under blocking conditions with administration of 3 mg∙kg^−1^ i.v. of the α_7_ nAChR partial agonist NS6740 at 3 min before radiotracer injection followed by a continuous infusion throughout the scan (1 mg∙kg^−1^∙h^−1^).

Arterial blood was sampled using a peristaltic pump during the first 20 min of the scan followed by manual sampling at 25, 30, 40, 50, 60, 90, 120, 150, 180, 210, and 240 min after injection. Plasma was obtained by centrifugation (13,000 rpm, 2 min at 21 °C) and total radioactivity concentration was measured using a gamma-counter (1470 Wizard; PerkinElmer, Shelton, CT, USA) cross-calibrated to the PET scanner. In addition, arterial whole blood was sampled manually at 4, 16, 30, 60, and 120 min p.i. and plasma obtained for metabolite analyses to determine the fraction of non-metabolized [^18^F]**10a**.

##### Arterial Input Function

The plasma input function was corrected for the presence of radioactive metabolites by determination of the parent fraction as described previously [12]. Plasma samples were de-proteinized by addition of ice-cold acetonitrile (500 μL MeCN/100 μL plasma) followed by centrifugation (13,000 rpm, 10 min). Aliquots of the original plasma sample and of the supernatant obtained after centrifugation were counted for radioactivity, and the supernatant was analyzed by radio-HPLC following the chromatographic methods described in quality control. The parent fraction as a function of time was fitted iteratively with the Levenberg-Marquardt algorithm, including decay correction, to calculate metabolite corrected input function [71].

##### Quantification of PET Data

After correction for attenuation, scatter, decay and scanner-specific dead time, images were reconstructed by filtered back-projection using a Hanning-filter of 4.9 mm FWHM into 40 frames of increasing length. A summed PET image of the 240-min scan was reconstructed for each animal and used for alignment with a T1-weighed MR image of a 6-week-old farm-bred pig as described previously [72]. The time-activity curves (TACs) were calculated for the following volumes of interest (VOIs): frontal cortex, temporal cortex, parietal cortex, occipital cortex, hippocampus, striatum (defined as mean radioactivity in caudate and putamen), cerebellum, thalamus, middle cortex, ventral cortex, midbrain, pons, and colliculi. Radioactivity in all VOIs was calculated as the average of radioactivity concentration (kBq∙cm^−3^) in the left and right sides. To generate standardized uptake values (SUVs) the TACs of the individual VOIs were normalized to the injected dose and corrected for animal weight (in kBq∙g^−1^). Kinetic analysis of the time-activity curves (TACs) was performed using an “in house” data analysis tool [73].

#### 3.7.4. PET Study in Rhesus Monkey

One male rhesus monkey (*Macaca mulatta*, 6 years old, body weight = 10 kg) underwent two PET imaging sessions with [^18^F]**10a** and [^18^F]**10b**, respectively. The scans were conducted 19 days apart. The monkey was initially sedated by intramuscular ketamine at 10 mg∙kg^−1^ at ~2 h before the PET scan and anesthetized by 1.5%–3% isoflurane throughout the imaging session. PET scan was performed using a FOCUS-220 PET scanner (Siemens Preclinical Solutions, Knoxville, TN, USA). After a transmission scan, 167 MBq of [^18^F]**10a** (specific activity of 362 GBq∙μmol^−1^) or 185 MBq of [^18^F]**10b** (specific activity of 159 GBq·μmol^−1^) were administered intravenously. Emission data were acquired in list mode for 240 min. Raw list-mode PET data was histogrammed (frames of 6 × 30 s; 3 × 1 min; 2 × 2 min; 46 × 5 min to scan termination) and reconstructed with 2D filtered back projection, using a 0.15 cm^−1^ Shepp filter and including corrections for scanner normalization, detector dead time, randoms, and radiation scatter and attenuation. The PET images were then registered to MR images, acquired with a Siemens 3 T Trio scanner, with a 6-parameter rigid body registration. The MR native space was then normalized using nonlinear affine registration to a high-resolution rhesus monkey atlas using BioImage Suite 3.01 (http://www.bioimagesuite.org/index.html). Time-activity curves were extracted by mapping atlas-defined regions to PET native space using the optimal transformation matrices calculated in the registration and normalization steps. Regions extracted included amygdala, caudate, cerebellum, cingulate, cortical regions (frontal, occipital, and temporal cortex), hippocampus, putamen, thalamus, and pons.

#### 3.7.5. Toxicity Studies in Rats

The acute toxicity of **10a** was assessed when administered by a single intravenous injection to rats followed by an observation period of 2 or 15 day (Hameln rds, Modra, Slovak Republic). The study was performed with 4 test groups, including 1 control and 3 dose groups (6.2, 62 and 620 μg∙kg^−1^), with 60 males and 60 females Wistar rats divided into two experiment cohorts: Day 2 (40 males and 40 females) and Day 15 (20 males and 20 females). The animals were weighed and allocated to the test groups based on their body weights. The animals were sacrificed in two time periods (day 2 and day 15 after test item administration) and then examined macroscopically and histopathologically. The following parameters were evaluated: mortality, clinical observation, body weights, food consumption, haematology (white blood cells, red blood cells, hemoglobin, hematocrit, mean corpuscular volume, mean corpuscular hemoglobin, platelets, lympohcytes, neutrophils, eosinophils, basophils, monocytes), clinical chemistry (alkaline phosphatase, aspartate aminotransferase, alanine aminotransferase, glucose, cholesterol, triacylglycerols, creatinine, urea, bilirubin, albumin, calcium, phosphorus, sodium, potassium chloride), pathology and histopathology.

## 4. Conclusions

We have designed and synthesized a number of compounds based on a new pharmacophore and tested their *in* *vitro* binding affinity and selectivity for α_7_ nAChR over other receptor subtypes. Among the new ligands, compound **10a** was found to have high α_7_ nAChR binding affinity and selectivity, and was selected for radiolabeling with ^18^F and evaluation as PET imaging radioligand for α_7_ nAChR *in* *vivo*. [^18^F]**10a** was produced in high radiochemical yield and purity both manually and in an automated synthesis. PET imaging experiments in piglets and monkey demonstrated that [^18^F]**10a** possessed appropriate pharmacokinetic properties and displayed specific and displaceable binding to α_7_ nAChR in the brain. Taken together, the new radioligand [^18^F]**10a** ([^18^F]DBT-10) appears to be a promising agent for PET imaging and quantification of α_7_ nAChR availability in the primate brain.

## Supplementary Materials

Supplementary materials including protocols for the preparation of intermediate compounds and NMR spectra of compounds **10**, **10a**, **10b**, **10c**, **11a**, **12a**, **12b**, **13b** and **14b** can be accessed at: http://www.mdpi.com/1420-3049/20/10/18387/s1.

## References

[1] Dineley K.T., Pandya A.A., Yakel J.L. (2015). Nicotinic ACh receptors as therapeutic targets in CNS disorders. Trends Pharmacol. Sci..

[2] Weiland S., Bertrand D., Leonard S. (2000). Neuronal nicotinic acetylcholine receptors: From the gene to the disease. Behav. Brain Res..

[3] Briggs C.A., Gronlien J.H., Curzon P., Timmermann D.B., Ween H., Thorin-Hagene K., Kerr P., Anderson D.J., Malysz J., Dyhring T. (2009). Role of channel activation in cognitive enhancement mediated by α_7_ nicotinic acetylcholine receptors. Br. J. Pharmacol..

[4] Papke R.L. (2014). Merging old and new perspectives on nicotinic acetylcholine receptors. Biochem. Pharmacol..

[5] Palma E., Conti L., Roseti C., Limatola C. (2012). Novel approaches to study the involvement of α_7_-nAChR in human diseases. Curr. Drug Targets.

[6] Horenstein N.A., Leonik F.M., Papke R.L. (2008). Multiple pharmacophores for the selective activation of nicotinic α_7_-type acetylcholine receptors. Mol. Pharmacol..

[7] Mazurov A., Hauser T., Miller C.H. (2006). Selective α_7_ nicotinic acetylcholine receptor ligands. Curr. Med. Chem..

[8] Kim S.W., Ding Y.S., Alexoff D., Patel V., Logan J., Lin K.S., Shea C., Muench L., Xu Y., Carter P. (2007). Synthesis and positron emission tomography studies of C-11-labeled isotopomers and metabolites of GTS-21, a partial α_7_ nicotinic cholinergic agonist drug. Nucl. Med. Biol..

[9] Toyohara J., Ishiwata K., Sakata M., Wu J., Nishiyama S., Tsukada H., Hashimoto K. (2010). *In*
*vivo* evaluation of α_7_ nicotinic acetylcholine receptor agonists [^11^C]A-582941 and [^11^C]A-844606 in mice and conscious monkeys. PLoS ONE.

[10] Hashimoto K., Nishiyama S., Ohba H., Matsuo M., Kobashi T., Takahagi M., Iyo M., Kitashoji T., Tsukada H. (2008). [^11^C]CHIBA-1001 as a novel PET ligand for α_7_ nicotinic receptors in the brain: A PET study in conscious monkeys. PLoS ONE.

[11] Ettrup A., Mikkelsen J.D., Lehel S., Madsen J., Nielsen E.Ø., Palner M., Timmermann D.B., Peters D., Knudsen G.M. (2011). ^11^C-NS14492 as a novel PET radioligand for imaging cerebral α_7_ nicotinic acetylcholine receptors: *In*
*vivo* evaluation and drug occupancy measurements. J. Nucl. Med..

[12] Deuther-Conrad W., Fischer S., Hiller A., Stergaard Nielsen E., Brunicardi Timmermann D., Steinbach J., Sabri O., Peters D., Brust P. (2009). Molecular imaging of α_7_ nicotinic acetylcholine receptors: Design and evaluation of the potent radioligand [^18^F]NS10743. Eur. J. Nucl. Med. Mol. Imaging.

[13] Rötering S., Scheunemann M., Fischer S., Hiller A., Peters D., Deuther-Conrad W., Brust P. (2013). Radiosynthesis and first evaluation in mice of [^18^F]NS14490 for molecular imaging of α_7_ nicotinic acetylcholine receptors. Bioorg. Med. Chem..

[14] Briggs C.A., Schrimpf M.R., Anderson D.J., Gubbins E.J., Grønlien J.H., Håkerud M., Ween H., Thorin-Hagene K., Malysz J., Li J. (2008). α_7_ nicotinic acetylcholine receptor agonist properties of tilorone and related tricyclic analogues. Br. J. Pharmacol..

[15] Schrimpf M.R., Sippy K.B., Briggs C.A., Anderson D.J., Li T., Ji J., Frost J.M., Surowy C.S., Bunnelle W.H., Gopalakrishnan M. (2012). SAR of α_7_ nicotinic receptor agonists derived from tilorone: Exploration of a novel nicotinic pharmacophore. Bioorg. Med. Chem. Lett..

[16] Teodoro R., Deuther-Conrad W., Rötering S., Scheunemann M., Patt M., Donat C.K., Wenzel B., Peters D., Sabri O., Brust P. (2014). Comparative evaluation of two novel fluorine-18 PET radiotracers for the α_7_ nicotinic acetylcholine receptor. J. Nucl. Med..

[17] Gao Y., Kellar K.J., Yasuda R.P., Tran T., Xiao Y., Dannals R.F., Horti A.G. (2013). Derivatives of dibenzothiophene for positron emission tomography imaging of α_7_-nicotinic acetylcholine receptors. J. Med. Chem..

[18] Horti A.G., Gao Y., Kuwabara H., Wang Y., Abazyan S., Yasuda R.P., Tran T., Xiao Y., Sahibzada N., Holt D.P. (2014). ^18^F-ASEM, a radiolabeled antagonist for imaging the α_7_-nicotinic acetylcholine receptor with PET. J. Nucl. Med..

[19] Horti A.G. (2015). Development of [^18^F]ASEM, a specific radiotracer for quantification of the α_7_-nAChR with positron-emission tomography. Biochem. Pharmacol..

[20] Wong D.F., Kuwabara H., Pomper M., Holt D.P., Brasic J.R., George N., Frolov B., Willis W., Gao Y., Valentine H. (2014). Human brain imaging of α_7_ nAChR with [^18^F]ASEM: A new PET radiotracer for neuropsychiatry and determination of drug occupancy. Mol. Imaging Biol..

[21] Deuther-Conrad W., Fischer S., Hiller A., Becker G., Cumming P., Xiong G., Funke U., Sabri O., Peters D., Brust P. (2011). Assessment of α_7_ nicotinic acetylcholine receptor availability in juvenile pig brain with [^18^F]NS10743. European J. Nucl. Med. Mol. Imaging.

[22] Toma L., Quadrelli P., Bunnelle W.H., Anderson D.J., Meyer M.D., Cignarella G., Gelain A., Barlocco D. (2002). 6-Chloropyridazin-3-yl derivatives active as nicotinic agents: Synthesis, binding, and modeling studies. J. Med. Chem..

[23] Eibl C., Tomassoli I., Munoz L., Stokes C., Papke R.L., Gündisch D. (2013). The 3,7-diazabicyclo[3.3.1]nonane scaffold for subtype selective nicotinic acetylcholine receptor (nAChR) ligands. Part 1: The influence of different hydrogen bond acceptor systems on alkyl and (hetero)aryl substituents. Bioorg. Med. Chem..

[24] Papke R.L., Schiff H.C., Jack B.A., Horenstein N.A. (2005). Molecular dissection of tropisetron, an α_7_ nicotinic acetylcholine receptor-selective partial agonist. Neurosci. Lett..

[25] Lehel S., Madsen J., Ettrup A., Mikkelsen J.D., Timmermann D.B., Peters D., Knudsen G.M. (2009). [^11^C]NS-12857: A novel PET ligand for α_7_-nicotinergic receptors. J. Labelled Compd. Rad..

[26] O’Donnell C.J., Peng L., O’Neill B.T., Arnold E.P., Mather R.J., Sands S.B., Shrikhande A., Lebel L.A., Spracklin D.K., Nedza F.M. (2009). Synthesis and SAR studies of 1,4-diazabicyclo[3.2.2]nonane phenyl carbamates—Subtype selective, high affinity α_7_ nicotinic acetylcholine receptor agonists. Bioorg. Med. Chem. Lett..

[27] Slowinski F., Ben Ayad O., Vache J., Saady M., Leclerc O., Lochead A. (2011). Synthesis of bridgehead-substituted azabicyclo[2.2.1]heptane and -[3.3.1]nonane derivatives for the elaboration of α_7_ nicotinic ligands. J. Org. Chem..

[28] Wawzonek S., Thelen P.J. (1950). Preparation of *N*-methylgranatanine. J. Am. Chem. Soc..

[29] Lambert F.L., Ellis W.D., Parry R.J. (1965). Halogenation of aromatic compounds by *N*-bromo- and *N*-chlorosuccinimide under ionic conditions. J. Org. Chem..

[30] Butts C.P., Eberson L., Hartshorn M.P., Radner F., Robinson W.T., Wood B.R. (1997). Regiochemistry of the reaction between dibenzothiophene radical cation and nucleophiles or nitrogen dioxide. Acta Chem. Scand..

[31] Gilman H., Nobis J.F. (1949). Some aminodibenzothiophenes. J. Am. Chem. Soc..

[32] Cullinane N.M., Davies C.G., Davies G.I. (1936). Substitution derivatives of diphenylene sulphide and diphenylenesulphone. J. Chem. Soc..

[33] Adams D.J., Clark J.H. (1999). Nucleophilic routes to selectively fluorinated aromatics. Chem. Soc. Rev..

[34] Boechat N., Clark J.H. (1993). Fluorodenitrations using tetramethylammonium fluoride. J. Chem. Soc. Chem. Commun..

[35] Manna S., Maity S., Rana S., Agasti S., Maiti D. (2012). Ipso-nitration of arylboronic acids with bismuth nitrate and perdisulfate. Org. Lett..

[36] Molander G.A., Cavalcanti L.N. (2012). Nitrosation of aryl and heteroaryltrifluoroborates with nitrosonium tetrafluoroborate. J. Org. Chem..

[37] Wu X.F., Schranck J., Neumann H., Beller M. (2011). Convenient and mild synthesis of nitroarenes by metal-free nitration of arylboronic acids. Chem. Commun..

[38] Xiao Y., Hammond P.S., Mazurov A.A., Yohannes D. (2012). Multiple interaction regions in the *orthosteric* ligand binding domain of the α_7_ neuronal nicotinic acetylcholine receptor. J. Chem. Inf. Model..

[39] Brunzell D.H., McIntosh J.M., Papke R.L. (2006). Diverse strategies targeting α_7_ homomeric and α_6_β_2_* heteromeric nicotinic acetylcholine receptors for smoking cessation. Ann. N. Y. Acad. Sci..

[40] Baddick C.G., Marks M.J. (2011). An autoradiographic survey of mouse brain nicotinic acetylcholine receptors defined by null mutants. Biochem. Pharmacol..

[41] Gotti C., Guiducci S., Tedesco V., Corbioli S., Zanetti L., Moretti M., Zanardi A., Rimondini R., Mugnaini M., Clementi F. (2010). Nicotinic acetylcholine receptors in the mesolimbic pathway: Primary role of ventral tegmental area α_6_β_2_* receptors in mediating systemic nicotine effects on dopamine release, locomotion, and reinforcement. J. Neurosci..

[42] Quik M., Polonskaya Y., Gillespie A., Jakowec M., Lloyd G.K., Langston J.W. (2000). Localization of nicotinic receptor subunit mRNAs in monkey brain by *in*
*situ* hybridization. J. Comp. Neurol..

[43] Han Z.Y., Le Novere N., Zoli M., Hill J.A., Champtiaux N., Changeux J.P. (2000). Localization of nAChR subunit mRNAs in the brain of Macaca mulatta. European J. Neurosci..

[44] Exley R., Maubourguet N., David V., Eddine R., Evrard A., Pons S., Marti F., Threlfell S., Cazala P., McIntosh J.M. (2011). Distinct contributions of nicotinic acetylcholine receptor subunit α_4_ and subunit α_6_ to the reinforcing effects of nicotine. Proc. Natl. Acad. Sci. USA.

[45] Faure P., Tolu S., Valverde S., Naudé J. (2014). Role of nicotinic acetylcholine receptors in regulating dopamine neuron activity. Neuroscience.

[46] Gotti C., Marks M.J., Millar N.S., Wonnacott S. Nicotinic acetylcholine receptors: α_6_. http://www.guidetopharmacology.org/GRAC/ObjectDisplayForward?objectId=467&familyId=76&familyType=IC.

[47] Le Novere N., Grutter T., Changeux J.P. (2002). Models of the extracellular domain of the nicotinic receptors and of agonist- and Ca^2+^-binding sites. Proc. Natl. Acad. Sci. USA.

[48] Ding M., Ghanekar S., Elmore C.S., Zysk J.R., Werkheiser J.L., Lee C.M., Liu J., Chhajlani V., Maier D.L. (2012). [^3^H]Chiba-1001(methyl-SSR180711) has low *in*
*vitro* binding affinity and poor *in*
*vivo* selectivity to nicotinic alpha-7 receptor in rodent brain. Synapse.

[49] Mazurov A.A., Kombo D.C., Akireddy S., Murthy S., Hauser T.A., Jordan K.G., Gatto G.J., Yohannes D. (2013). Novel nicotinic acetylcholine receptor agonists containing carbonyl moiety as a hydrogen bond acceptor. Bioorg. Med. Chem. Lett..

[50] Girard B.M., Merriam L.A., Tompkins J.D., Vizzard M.A., Parsons R.L. (2013). Decrease in neuronal nicotinic acetylcholine receptor subunit and PSD-93 transcript levels in the male mouse MPG after cavernous nerve injury or explant culture. Am. J. Physiol..

[51] Glushakov A.V., Voytenko L.P., Skok M.V., Skok V. (2004). Distribution of neuronal nicotinic acetylcholine receptors containing different alpha-subunits in the submucosal plexus of the guinea-pig. Auton. Neurosci. Basic Clin..

[52] Teodoro R., Wenzel B., Oh-Nishi A., Fischer S., Peters D., Suhara T., Deuther-Conrad W., Brust P. (2015). A high-yield automated radiosynthesis of the alpha-7 nicotinic receptor radioligand [^18^F]NS10743. Appl. Radiat. Isot..

[53] Jacobson O., Chen X. (2010). PET designated flouride-18 production and chemistry. Curr. Top. Med. Chem..

[54] Ravert H.T., Holt D.P., Gao Y., Horti A.G., Dannals R.F. (2015). Microwave-assisted radiosynthesis of [^18^F]ASEM, a radiolabeled α_7_-nicotinic acetylcholine receptor antagonist. J. Labelled Compd. Radiopharm..

[55] Egleton R.D., Brown K.C., Dasgupta P. (2009). Angiogenic activity of nicotinic acetylcholine receptors: Implications in tobacco-related vascular diseases. Pharmacol. Ther..

[56] Cooke J.P., Bitterman H. (2004). Nicotine and angiogenesis: A new paradigm for tobacco-related diseases. Ann. Med..

[57] Pena V.B., Bonini I.C., Antollini S.S., Kobayashi T., Barrantes F.J. (2011). α_7_-Type acetylcholine receptor localization and its modulation by nicotine and cholesterol in vascular endothelial cells. J. Cell. Biochem..

[58] Innis R.B., Cunningham V.J., Delforge J., Fujita M., Gjedde A., Gunn R.N., Holden J., Houle S., Huang S.C., Ichise M. (2007). Consensus nomenclature for *in*
*vivo* imaging of reversibly binding radioligands. J. Cereb. Blood Flow Metab..

[59] Cunningham V.J., Rabiner E.A., Slifstein M., Laruelle M., Gunn R.N. (2010). Measuring drug occupancy in the absence of a reference region: The Lassen plot re-visited. J. Cereb. Blood Flow Metab..

[60] Brust P., Peters D., Deuther-Conrad W. (2012). Development of radioligands for the imaging of α_7_ nicotinic acetylcholine receptors with positron emission tomography. Curr. Drug Targets.

[61] Harwood L.M. (1985). “Dry-column” flash chromatography. Aldrichimica Acta.

[62] Schrimpf M., Sippy K., Ji J., Li T., Frost J., Briggs C., Bunnelle W. (2005). Amino-Substituted Tricyclic Derivatives and Methods of Use. U.S. Patent.

[63] Rötering S., Franke K., Zessin J., Brust P., Füchtner F., Fischer S., Steinbach J. (2015). Convenient recycling and reuse of bombarded [^18^O]H_2_O for the production and the application of [^18^F]F. Appl. Radiat. Isot..

[64] Charpantier E., Wiesner A., Huh K.H., Ogier R., Hoda J.C., Allaman G., Raggenbass M., Feuerbach D., Bertrand D., Fuhrer C. (2005). α_7_ neuronal nicotinic acetylcholine receptors are negatively regulated by tyrosine phosphorylation and Src-family kinases. J. Neurosci..

[65] Cheng Y., Prusoff W.H. (1973). Relationship between the inhibition constant (*K*_1_) and the concentration of inhibitor which causes 50 per cent inhibition (IC_50_) of an enzymatic reaction. Biochem. Pharmacol..

[66] Michelmore S., Croskery K., Nozulak J., Hoyer D., Longato R., Weber A., Bouhelal R., Feuerbach D. (2002). Study of the calcium dynamics of the human α_4_β_2_, α_3_β_4_ and α_1_β_1_γδ nicotinic acetylcholine receptors. Naunyn-Schmiedeberg’s Arch. Pharmacol..

[67] Grady S.R., Moretti M., Zoli M., Marks M.J., Zanardi A., Pucci L., Clementi F., Gotti C. (2009). Rodent habenulo-interpeduncular pathway expresses a large variety of uncommon nAChR subtypes, but only the αβ_4_* and αβ_3_β_4_* subtypes mediate acetylcholine release. J. Neurosci..

[68] Pucci L., Grazioso G., Dallanoce C., Rizzi L., De Micheli C., Clementi F., Bertrand S., Bertrand D., Longhi R., De Amici M. (2011). Engineering of α-conotoxin MII-derived peptides with increased selectivity for native α_6_β_2_* nicotinic acetylcholine receptors. FASEB J..

[69] Munson P.J., Rodbard D. (1980). Ligand: A versatile computerized approach for characterization of ligand-binding systems. Anal. Biochem..

[70] Brust P., Patt J.T., Deuther-Conrad W., Becker G., Patt M., Schildan A., Sorger D., Kendziorra K., Meyer P., Steinbach J. (2008). *In*
*vivo* measurement of nicotinic acetylcholine receptors with [^18^F]norchloro-fluoro-homoepibatidine. Synapse.

[71] Gillings N.M., Bender D., Falborg L., Marthi K., Munk O.L., Cumming P. (2001). Kinetics of the metabolism of four PET radioligands in living minipigs. Nucl. Med. Biol..

[72] Brust P., Zessin J., Kuwabara H., Pawelke B., Kretzschmar M., Hinz R., Bergman J., Eskola O., Solin O., Steinbach J. (2003). Positron emission tomography imaging of the serotonin transporter in the pig brain using [^11^C](+)-McN5652 and S-[^18^F]fluoromethyl-(+)-McN5652. Synapse.

[73] Brust P., Hinz R., Kuwabara H., Hesse S., Zessin J., Pawelke B., Stephan H., Bergmann R., Steinbach J., Sabri O. (2003). *In*
*vivo* measurement of the serotonin transporter with (*S*)-([^18^F]fluoromethyl)-(+)-McN5652. Neuropsychopharmacology.

