# Supplementary material for: A Promising PET Tracer for Imaging of α7 Nicotinic Acetylcholine Receptors in the Brain: Design, Synthesis, and in Vivo Evaluation of a Dibenzothiophene-Based Radioligand
Source: Molecules. 2015 Oct 9;20(10):18387–421. doi: 10.3390/molecules201018387 (PMC6332508; doi:10.3390/molecules201018387)
Supplement: Supplementary file 1 [file molecules-20-18387-s001.pdf]

Supplementary Materials

Table of Contents

Organic Chemistry: General Methods	S2
Abbreviations	S2
Synthetic Procedures	S2
Synthesis of brominated dibenzo*[b,d]*thiophene sulfone based starting materials	S2
Synthesis of **10**, Fluorinated Reference Compounds **10a**–**c**, **12a**,**b**, **13b**, **14b** and Nitro Labelling Precursor **11a** for Synthesis of [^{18}F]**10a**	S7
Images of NMR Spectra	S13
Compound **10a**	S13
Compound **10b**	S13
Compound **10c**	S14
Compound **10**	S15
Compound **11a**	S16
Compound **12a**	S17
Compound **12b**	S18
Compound **13b**	S19
Compound **14b**	S20
References	S21

Organic Chemistry: General Methods

Analytical thin-layer chromatography (TLC) was performed with Macherey-Nagel precoated plastic sheets with fluorescent indicator UV_{254} (Polygram® SIL G/UV_{254}, Macherey-Nagel GmbH & Co. KG, Düren, Germany). Visualization of the spots was effected by illumination with an UV lamp (254 nm and 366 nm). NMR spectra (^{1}H, ^{13}C, ^{13}C-APT, ^{19}F, COSY, HSQC, HMBC) were recorded with Varian spectrometer (Varian Mercury-300BB and Mercury-400BB). Chemical shifts are reported as δ (δ_H, δ_C, δ_F) values. Coupling constants are reported in Hertz. Multiplicity is defined by s (singlet), d (doublet), t (triplet), and combinations thereof; br (broad) and m (multiplet). ESI-ion trap mass spectra (LRMS) were recorded with a Bruker Esquire 3000 plus. High resolution mass spectra were recorded on a FT-ICR APEX II (Bruker Daltonics, Bruker Corporation, Billerica, MA, USA) using electrospray ionization (ESI) in positive ion mode. Melting points were determined on a Linström capillary melting point apparatus (Wagner & Munz GmbH, Vienna, Austria) in open capillary tubes and are uncorrected.

Chromatographic purification was performed as dry-column flash chromatography (DCFC) [1–4] with vacuum on silica gel 60 (particle size 15–40 µm, Ref. 815650.5) from Macherey-Nagel.

Reagents and solvents were purchased from commercial sources and used without further purification unless otherwise noted. Compounds **2** [5], **4a** [6,7] and **5a** [8,9] were prepared starting from **1a** (dibenzo[*b*,*d*]thiophene, Acros Organics, Geel, Belgium) according to literature methods. The intermediate compounds **3** [10,11], **5b** [12], **8a** [12], **8b** [12] and final compound **10** [13] have been already described, however, with different synthetic routes or starting from precursors not used in our protocols. Intermediate compounds **6a** (two steps from **4a**), **6b** (one step from **5b**) and final compounds **10a**, **10b**, and **11a** (labeling precursor for [^{18}F]**10a**) have been prepared using protocols according to literature [14,13], and similarly to those already used by Gao *et al.* [12] Intermediate compounds **7a**, **8c**, **8d** and final compounds **10c**, **12a**, **12b**, **13b** and **14b** have not been reported so far, to the best of our knowledge.

Abbreviations Used

MeOH, methanol; EtOH, ethanol; MeCN, acetonitrile; DMF, dimethylformamide; EtOAc, ethyl acetate; THF, tetrahydrofuran; TEA, triethylamine; $Pd_2(dba)_3$, *tris*(dibenzylideneacetone)dipalladium; BINAP, racemic 2,2′-bis(diphenylphosphino)-1,1′-binaphthyl; NBS, *N*-bromosuccinimide; TMAF·4 H_2O, tetramethylammonium fluoride tetrahydrate, DCFC, dry-column flash chromatography, TLC, thin-layer chromatography.

Synthetic Procedures

Synthesis of brominated dibenzo[*b*,*d*]thiophene sulfone based starting materials

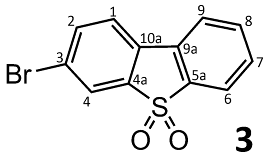

3

3-Bromo-dibenzo[b,d]thiophene 5,5-dioxide (**3**). Representative procedure for NBS bromination. Compound **3** was prepared in two steps from dibenzo[*b*,*d*]thiophene (**1a**). First, oxidation of **1a** with

H_2O_2 in acetic acid [5] yielded the corresponding sulfone (**2**) in 97% yield. Dibenzo[*b*,*d*]thiophene 5,5-dioxide **2** (0.67 g, 3.1 mmol) was dissolved in H_2SO_4 (96%, 10 mL). NBS (0.52 g, 2.9 mmol) was added to this solution in several portions and the mixture was stirred at room temperature for 24 h. The formed suspension was poured into ice-water (60 mL) and the solid was filtered off, washed and dried (0.87 g). TLC analysis (cyclohexane/CH_2Cl_2, 2:3) showed a mixture of starting material (R_f = 0.19), 3-bromo (R_f = 0.31) and 3,7-dibromo derivative (R_f = 0.43) as well. The solid was dissolved in hot MeCN (20 mL) and the portion of crystals formed under cooling, which is enriched in 3,7-dibromo derivative (0.22 g) as identified by TLC was removed. The remaining material obtained from the filtrate (0.647 g) was subjected to purification by DCFC (silica gel 15–40 µm, 26 g) with a gradient from cyclohexane/$CHCl_3$ (2:3, 100%) to cyclohexane/$CHCl_3$ (1:3, 100%). The fraction containing the 3-bromo derivative was evaporated and the residual solid (0.485 g) was recrystallized from EtOH to give 0.38 g (1.28 mmol, 44% yield) as colorless crystals, m.p. 219–221 °C (lit m.p. 224–225 °C. [10,11]); ^{1}H-NMR (400 MHz, $CDCl_3$) δ 7.55 (td, *J* = 7.6, 1.0 Hz, 1H), 7.64 (d, *J* = 8.2 Hz, 1H), 7.65 (td, *J* = 7.7, 1.2 Hz, 1H), 7.74 (dd, *J* = 8.2, 1.8 Hz, 1H), 7.76 (d, *J* = 8.1 Hz, 1H), 7.81 (d, *J* = 7.6 Hz, 1H), 7.93 (d, *J* = 1.7 Hz, 1H); ^{13}C-NMR (100 MHz, $CDCl_3$) δ 139.35, 137.56, 137.02, 134.25, 130.84, 130.82, 130.60, 125.56, 124.35, 123.07, 122.43, 121.74.

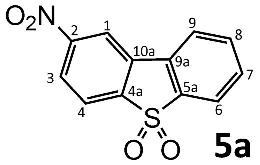

5a

2-Nitrodibenzo[b,d]thiophene 5,5-dioxide (**5a**). Representative procedure for sulfide to sulfone oxidation [8,9]. A mixture of 2-nitrodibenzo[*b*,*d*]thiophene (**4a**, [6,7] 2.3 g, 10 mmol) in acetic acid (64 mL) was stirred at 80 °C while H_2O_2 (50%, 6.8 mL, 0.12 mol) was added. The temperature was raised to 125 °C and after 1 h a second portion H_2O_2 (50%, 4.5 mL, 0.08 mol) was added. Stirring at 120 °C was continued for 1 h and at 80 °C for 3 h. After cooling, the mixture was poured into water (180 mL) to form a precipitate which was filtered, thoroughly washed and dried to yield the pure title compound **5a** (2.52 g, 9.64 mmol, 96% yield, R_f = 0.22, cyclohexane/$CHCl_3$, 1:3) as a pale yellow powder, m.p. 256.5–257.5 °C (lit m.p. 258 °C [8], 257–258 °C [9]);^{1}H-NMR (400 MHz, DMSO-d_6) δ_H 7.74 (td, *J* = 7.6, 0.9 Hz, $1H_{Ar}$, 7-H), 7.87 (td, *J* = 7.6, 1.0 Hz, $1H_{Ar}$, 8-H), 8.07 (d, *J* = 7.7 Hz, $1H_{Ar}$, 6-H), 8.29 (m, $1H_{Ar}$, 9-H), 8.42 (dd, *J* = 8.4, 2.0 Hz, $1H_{Ar}$, 3-H), 8.48 (d, *J* = 7.8 Hz, $1H_{Ar}$, 4-H), 9.04 (d, *J* = 2.0 Hz, $1H_{Ar}$, 1-H). ^{13}C-NMR (100 MHz, DMSO-d_6) δ_C 118.26 (CH, 3-C), 122.28 (CH, 1-C), 123.60 (CH, 6-C), 123.95 (CH, 9-C), 126.10 (CH, 4-C), 129.10 (C, 9a-C), 132.09 (CH, 7-C), 132.98 (C, 10a-C), 135.04 (CH, 8-C), 137.08 (C, 5a-C), 141.44 (C, 4a-C), 151.72 (C, 2-C); LRMS: *m*/*z* (ESI) = 284.0 $(M + Na)^+$.

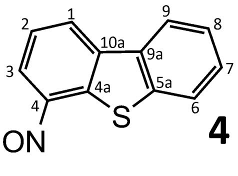

4

4-Nitrosodibenzo[b,d]thiophene (**4**). *Tert*-butyl nitrite (2.14 mL, 1.86 g, 18 mmol) was added in one portion to a stirred white suspension of dibenzo[*b*,*d*]thiophen-4-yl boronic acid (**1b**, Frontier Scientific,

Logan, UT, USA, 1.37 g, 6 mmol) in MeCN (24 mL) under argon at 22 °C. The mixture was stirred at 50–55 °C while its colour turned from yellow to dark brown in the course of 24 h. The solvent was evaporated and the residual solid was dissolved in CH_2Cl_2 (80 mL), washed with H_2O (25 mL), dried ($MgSO_4$) and evaporated to leave a dark greenish brown residue (1.38 g) which was used for the oxidation step. A sample (40 mg) was dissolved in cyclohexane/CH_2Cl_2 (2:3, *v*/*v*, 3 mL) and filtered through a short plug of silica gel (15–40 µm, 2 g). Subsequent elution with cyclohexane/CH_2Cl_2 (2:3, *v*/*v*, 50 mL) gave a green eluate which was evaporated to yield 30 mg of the title compound (**4**, R_f = 0.56, heptane/EtOAc, 3:1) as a green powder, m.p 113–115 °C (lit m.p. 115–117 °C [15]; The product is 88% pure as calculated by ^{1}H-NMR and accompanied by 12% 4-nitrodibenzo[*b*,*d*]thiophene (**4b**) as minor portion, as determined by comparison with ^{1}H-NMR of a pure sample. Compound **4**: ^{1}H-NMR (300 MHz, $CDCl_3$) δ 7.48–7.63 (m, 2H, 7-H, 8-H), 7.88–7.94 (m, 1H, 6-H), 7.94 (t, *J* =7.7 Hz, 1H, 2-H), 8.13–8.22 (m, 1H, 9-H), 8.49 (dd, *J* = 7.7, 1.1 Hz, 1H, 3-H), 9.58 (dd, *J* = 7.6, 1.1 Hz, 1H, 1-H); ^{13}C-NMR (75 MHz, $CDCl_3$) δ 120.10 (C), 121.68 (CH), 123.67 (CH), 125.44 (CH), 125.84 (CH), 127.82 (CH), 128.21 (CH), 132.38 (C), 137.94 (C), 138.11 (C), 142.04 (C), 161.91 (C), 165.95 (C). Compound **4b** (obtained and isolated in pure form as by-product from nitration of **1**, [6,7] in 2% yield [not described herein]): ^{1}H-NMR (300 MHz, $CDCl_3$) δ_H 7.48–7.59 (m, 2H, 7-H, 8-H), 7.62 (t, *J* = 8.0 Hz, 1H, 2-H), 7.98–7.90 (m, 1H, H-6), 8.23–8.15 (m, 1H, H-9), 8.45 (dd, *J* = 7.8, 0.9 Hz, 1H, 3-H), 8.49 (dd, *J* = 8.1, 0.8 Hz, 1H, 1-H); ^{13}C-NMR (75 MHz, $CDCl_3$) δ_C 121.98 (1C, CH), 122.89 (1C, CH), 123.39 (1C, CH), 124.83 (1C, CH), 125.43 (1C, CH), 127.38 (1C, CH), 128.30 (1C, CH), 133.96 (1C, C), 135.57 (1C, C), 138.94 (1C, C), 141.08 (1C, C), 143.03 (1C, C).

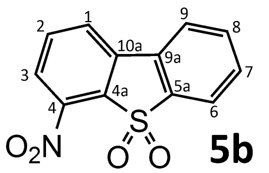

5b

4-Nitrodibenzo[b,d]thiophene 5,5-dioxide (**5b**). According to the representative procedure for sulfide to sulfone oxidation compound **4** (1.33 g, 5.8 mmol) was reacted with H_2O_2 (6.6 mL, 0.11 g, 116 mmol) in acetic acid to give 1.333 g of a dark yellow solid. The product was further purified by DCFC (silica gel 15–40 µm, 24 g) with cyclohexane/CH_2Cl_2 (1:1→1:2) to yield the pure title compound **5b** (1.1 g, 4.2 mmol, 72% yield, R_f = 0.2, $CHCl_3$) as pale yellow crystals, m.p. 245.5–251 °C; ^{1}H-NMR (400 MHz, DMSO-d_6) δ_H 7.75 (td, *J* = 7.6, 0.8 Hz, $1H_{Ar}$, 7-H), 7.86 (td, *J* = 7.6, 1.0 Hz, $1H_{Ar}$, 8-H), 8.04 (d, *J* = 7.7 Hz, $1H_{Ar}$), 8.08 (t, *J* = 8.0 Hz, $1H_{Ar}$), 8.29 (d, *J* = 7.7 Hz, $1H_{Ar}$), 8.39 (dd, *J* = 8.2, 0.7 Hz, $1H_{Ar}$), 8.65 (dd, *J* = 7.7, 0.7 Hz, $1H_{Ar}$); ^{13}C-NMR (100 MHz, DMSO-d_6) δ_C 122.19 (CH), 123.21 (CH), 125.91 (CH), 128.29 (C), 129.16 (CH), 130.58 (C), 132.18 (CH), 134.45 (C), 134.71 (CH), 136.21 (CH), 137.26 (C), 143.39 (C, 4-C); LRMS: *m*/*z* (ESI) = 284.0 (M + Na)$^+$.

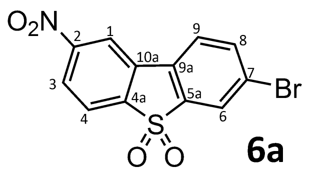

6a

7-Bromo-2-nitrodibenzo[b,d]thiophene 5,5-dioxide (**6a**). According to the representative procedure for NBS bromination compound **5a** (1.752 g, 6.7 mmol) was treated with NBS (1.28 g, 7.2 mmol) in H_2SO_4 (96%, 30 mL). The yellowish solid (2.287 g) obtained after aqueous work-up was recrystallized twice from MeCN to afford the pure title compound **6a** (1.45 g, 4.27 mmol, 63.8% yield, R_f = 0.30, cyclohexane/$CHCl_3$, 1:4) as a pale yellow powder, m.p. 281.0–282.5 °C; ^{1}H NMR (400 MHz, DMSO-d_6) δ_H 8.08 (dd, J = 8.4, 1.8 Hz, 1H$_{Ar}$, 8-H), 8.31 (d, J = 8.4 Hz, 1H$_{Ar}$, 4-H), 8.41–8.47 (m, 3H$_{Ar}$, 9-H, 3-H, 6-H), 9.07 (d, J = 1.8 Hz, 1H$_{Ar}$, 1-H); ^{13}C-NMR (100 MHz, DMSO-d_6) δ_C 118.52 (CH, 1-C), 123.72 (CH, 4-C), 125.11 (C, 7-C), 125.36 (CH, 9-C), 125.85 (CH, 6-C), 126.35 (CH, 3-C), 128.41 (C, 9a-C), 132.19 (C, 10a-C), 137.90 (CH, 8-C), 138.66 (C, 5a-C), 141.11 (C, 4a-C), 151.78 (C, 2-C); LRMS: *m*/*z* (ESI) = 364.0, 362.0 (M + Na)$^+$.

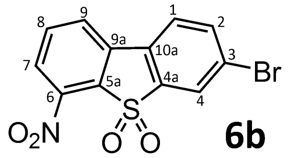

6b

3-Bromo-6-nitrodibenzo[b,d]thiophene 5,5-dioxide (**6b**). According to the representative procedure for NBS bromination, compound **5b** (0.64 g, 2.45 mmol) was treated with NBS (0.44 g, 2.48 mmol) in H_2SO_4 (96%, 8.6 mL). The yellowish solid (0.826 g) obtained after aqueous work-up was recrystallized from MeCN to afford the pure title compound **6b** (0.65 g, 1.9 mmol, 77.5% yield, R_f = 0.30, $CHCl_3$) as a pale yellow powder, m.p. 288–301 °C (dec.); ^{1}H-NMR (400 MHz, DMSO-d_6) δ_H 8.08 (dd, J = 8.3, 1.8 Hz, 1H$_{Ar}$, 2-H), 8.10 (t, J = 8.0 Hz, 1H$_{Ar}$, 8-H), 8.26 (d, J = 8.3 Hz, 1H$_{Ar}$), 8.41 (d, J = 1.7 Hz, 1H$_{Ar}$, 4-H), 8.43 (dd, J = 8.2, 0.7 Hz, 1H$_{Ar}$, 1-H), 8.68 (dd, J = 7.7, 0.7 Hz, 1H); ^{13}C-NMR (100 MHz, DMSO-d_6) δ_C 125.16 (CH, 1-C [or 4-C]), 125.21 (CH, 4-C [or 1-C]), 126.19 (CH, 7-C), 127.62 (C, 3-C), 129.36 (CH, 9-C), 130.29 (C, 10a-C), 133.64 (C, 9a-C), 136.43 (CH, 8-C), 137.37 (C, 5a-C), 137.60 (CH, 2-C), 138.86 (C, 4a-C), 143.35 (C, 6-C); LRMS: *m*/*z* (ESI) = 363.9, 362.1 (M + Na)$^+$.

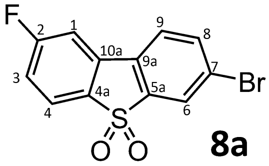

8a

7-Bromo-2-fluorodibenzo[b,d]thiophene 5,5-dioxide (**8a**). Representative procedure for fluorodenitration with TMAF. TMAF tetrahydrate (0.215 g, 1.3 mmol, Acros Organics) was dried under an atmosphere of argon by azeotropic distillation with a mixture of DMSO (6 mL) and cyclohexane (12 mL) using a water separator for 6 h (bath: 115–120 °C). The bath temperature was allowed to cool to 80 °C and the nitro derivative **6a** (0.34 g, 1 mmol) was added under stirring to the suspension of the dried TMAF in one portion. The mixture immediately turned dark brown and was stirred for additional 2 h at 95 °C. Reaction progress was monitored by TLC (cyclohexane/$CHCl_3$, 1:4). The portion of cyclohexane was evaporated. The residual oil was poured into water (60 mL) and extracted with CH_2Cl_2 (4 × 15 mL). The extracts were combined and washed with water (10 mL), dried ($MgSO_4$) and evaporated. The residual yellow solid (0.283 g) was purified by DCFC (silica gel 15–40 µm, 6 g) with CH_2Cl_2. An eluate (40 mL) was collected and evaporated to leave a solid, which was triturated with EtOH (2 mL), filtered and dried to afford the

pure title compound **8a** (0.23 g, 0.74 mmol, 73.7% yield, R_f = 0.39, cyclohexane/$CHCl_3$, 1:4), as colorless powder, m.p. 266.5–268.5 °C; ^{1}H-NMR (300 MHz, DMSO-*d*6) δ_H 7.52 (dt-like, *J* = 8.8, 8.7, 2.4 Hz, $1H_{Ar}$, 3-H), 8.05 (dd, *J* = 8.3, 1.8 Hz, $1H_{Ar}$, 8-H), 8.11 (dd, *J* = 8.6, 4.9 Hz, $1H_{Ar}$, 4-H), 8.16 (d, *J* = 8.3 Hz, $1H_{Ar}$, 9-H), 8.18 (dd, *J* = 9.2, 2.3 Hz, $1H_{Ar}$, 1-H), 8.35 (d, *J* = 1.6 Hz, $1H_{Ar}$, 6-H); ^{13}C NMR (75 MHz, DMSO-*d*6) δ_C 110.74 (d, $^2J_{CF}$ = 25.6 Hz, CH, 3-C), 118.22 (d, $^2J_{CF}$ = 24.1 Hz, CH, 1-C), 124.53 (s, C, 7-C), 124.89 (d, $^3J_{CF}$ = 10.2 Hz, CH, 4-C), 125.09 (s, CH, 6-C [or 9-C]), 125.16 (s, CH, 9-C [or 6-C]), 128.98 (d, $^4J_{CF}$ = 2.4 Hz, C, 9a-C), 132.82 (d, $^4J_{CF}$ = 3.0 Hz, C, 4a-C), 133.45 (d, $^3J_{CF}$ = 10.7 Hz, C, 10a-C), 137.49 (s, CH, 8-C), 139.31 (s, C, 5a-C), 165.85 (d, $^1J_{CF}$ = 252.2 Hz, C, 2-C); ^{19}F-NMR (282.36 MHz, DMSO-d6) δ_F −103.8 (m, F_{Ar}, 2-F); LRMS: *m*/*z* (ESI) = 334.9, 336.9 $(M + Na)^+$.

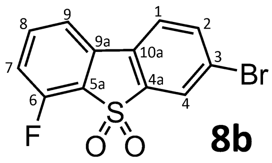

8b

3-Bromo-6-fluorodibenzo[b,d]thiophene 5,5-dioxide (**8b**). According to the representative procedure for fluorodenitration, TMAF tetrahydrate (0.616 g, 3.73 mmol) was azeotropically dried with cyclohexane/DMSO and subsequently reacted with compound **6b** (0.94 g, 2.76 mmol). After work-up, the obtained yellow solid (0.86 g) was purified by DCFC (silica gel 15–40 µm, 24 g) with cyclohexane/CH_2Cl_2 (1:1→1:3) to yield 0.63 g of the pure title compound (**8b**, 2.0 mmol, 72.9% yield, R_f = 0.52, $CHCl_3$), as colorless powder, m.p. 221–222 °C; ^{1}H-NMR (300 MHz, $CDCl_3$) δ_H 7.21 (td, *J* = 8.2, 0.8 Hz, $1H_{Ar}$, 8-H),7.55 (dd, *J* = 7.6, 0.7 Hz, 1H), 7.69–7.59 (m, 2H), 7.77 (dd, *J* = 8.2, 1.8 Hz, 1H), 7.93 (d, *J* = 1.7 Hz, $1H_{Ar}$, 4-H); ^{13}C-NMR (75 MHz, $CDCl_3$) δ_C 117.49 (d, $^4J_{CF}$ = 3.6 Hz, CH, 9-C), 118.32 (d, $^2J_{CF}$ = 19.3 Hz, CH, 7-C), 123.47 (s, CH, 4-C), 125.07 (s, C, 3-C), 125.58 (s, CH, 1-C), 129.66 (d, $^4J_{CF}$ = 2.7 Hz, C, 10a-C), 133.62 (d, $^3J_{CF}$ = 2.5 Hz, C, 9a-C), 136.63 (d, $^3J_{CF}$ = 7.8 Hz, CH, 8-C), 137.17 (s, CH, 2-C), 139.92 (d, $^4J_{CF}$ = 0.8 Hz, C, 4a-C), 158.00 (d, $^1J_{CF}$ = 259.5 Hz, C, 6-C), one of the ring-fused carbons (5a-C) could not be detected; ^{19}F NMR (282 MHz, $CDCl_3$) δ_F −114.14 (m, 1 F_{Ar}, 6-F); LRMS: *m*/*z* (ESI) = 336.9, 335.0 $(M +Na)^+$.

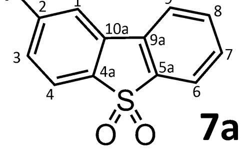

7a

2-Fluorodibenzo[b,d]thiophene 5,5-dioxide (**7a**). According to the representative procedure for fluorodenitration, TMAF tetrahydrate (1.35 g, 8.18 mmol) was azeotropically dried with cyclohexane/DMSO and subsequently reacted with compound **5a** (1.525 g, 5.84 mmol). After work-up, the obtained yellow solid (1.25 g) was purified by DCFC (silica gel 15–40 µm, 18 g) with cyclohexane/CH_2Cl_2 (1:1→1:3) as eluent to yield the pure title compound **7a** (1.03 g, 4.4 mmol, 75.2% yield, R_f = 0.4, cyclohexane/$CHCl_3$, 1:4), as colorless powder, m.p. 227–228 °C; ^{1}H-NMR (400 MHz, $CDCl_3$) δ_H 7.21 (dt, *J* = 8.45, 8.45, 2.27 Hz, $1H_{Ar}$, H-3), 7.45 (dd, *J* = 8.37, 2.26 Hz, $1H_{Ar}$, H-1), 7.57 (dt, *J* = 7.56, 7.53, 1.09 Hz, $1H_{Ar}$, H-7), 7.66 (dt, *J* = 7.65, 7.58, 1.17 Hz, $1H_{Ar}$, H-8), 7.75 (ddd, *J* = 7.70, 1.01, 0.57 Hz, $1H_{Ar}$, 9-H), 7.82 (dd, *J* = 8.42, 4.92 Hz, $1H_{Ar}$, H-4), 7.83 (ddd, *J* = 7.60, 1.09, 0.69 Hz, $1H_{Ar}$,

6-H); ^{13}C-NMR (100 MHz, $CDCl_3$) δ_C 109.34 (d, $^2J_{CF}$ = 24.6 Hz, CH, 3-C), 117.61 (d, $^2J_{CF}$ = 23.9 Hz, CH, 1-C), 121.98 (s, CH, 6-C), 122.40 (s, CH, 9-C), 124.58 (d, $^3J_{CF}$ = 9.9 Hz, CH, 4-C), 130.47 (d, $^4J_{CF}$ = 2.5 Hz, C, 9a-C), 131.23 (s, CH, 7-C), 133.72 (d, $^4J_{CF}$ = 3.2 Hz, C, 4a-C), 134.13 (s, CH, 8-C), 134.92 (d, $^3J_{CF}$ = 9.7 Hz, C, 10a-C), 138.68 (s, C, 5a-C), 166.37 (d, $^1J_{CF}$ = 255.0 Hz, C, 2-C); ^{19}F-NMR (376 MHz, $CDCl_3$) δ_F −103.63 (m. 1F_{Ar}, 2-F); LRMS: *m/z* (ESI) = 257.0, 258.0 $(M + Na)^+$.

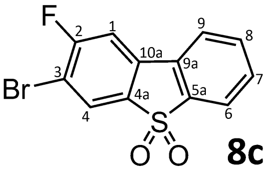

8c

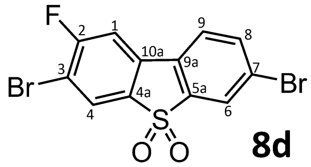

8d

3-Bromo-2-fluorodibenzo[b,d]thiophene 5,5-dioxide (**8c**) *and 3,7-dibromo-2-fluorodibenzo[b,d] thiophene 5,5-dioxide* (**8d**). According to the representative procedure for bromination, compound **7a** (0.562 g, 2.4 mmol) was reacted with NBS (0.43 g, 2.4 mmol) in H_2SO_4. The solid residue obtained upon work-up (0.751 g) consisted of a mixture of starting material (R_f = 0.32), mono- (R_f = 0.43) and dibrominated derivative (R_f = 0.56) as shown by TLC analysis (cyclohexane/$CHCl_3$, 1:4). The mixture was subjected to purification by DCFC (silica gel 15–40 µm, 28 g) with cyclohexane/$CHCl_3$ (1:3, 100%) to yield the 2-fluoro-3,7-dibromo derivative **8d** (0.17 g, 0.43 mmol, 18%) from the first fraction and the 2-fluoro-3-bromo derivative **8c** (0.35 g, 1.12 mmol, 46.5%) from the second fraction. Compound **8c**: m.p. 269–274.5 °C; ^{1}H-NMR (400 MHz, DMSO-d_6) δ_H 7.72 (t, J = 7.6 Hz, 1H_{Ar}, 7-H), 7.84 (t, J = 7.6 Hz, 1H_{Ar}, 8-H), 8.01 (d, J = 7.7 Hz, 1H_{Ar}, 6-H), 8.22 (d, J = 7.7 Hz, 1H_{Ar}, 9-H), 8.32 (d, J = 8.9 Hz, 1H_{Ar}, 1-H), 8.55 (d, J = 6.2 Hz, 1H_{Ar}, 4-H); ^{13}C-NMR (100 MHz, DMSO-d_6) δ_C 110.78 (d, $^2J_{CF}$ = 23.4 Hz, C, 3-C), 111.57 (d, $^2J_{CF}$ = 26.5 Hz, CH, 1-C), 122.07 (s, CH, 9-C), 123.33 (s, CH, 6-C), 127.71 (d, $^3J_{CF}$ = 1.5 Hz, CH, 4-C), 129.16 (d, $^4J_{CF}$ = 2.1 Hz, C, 9a-C), 131.80 (s, CH, 7-C), 133.30 (d, $^3J_{CF}$ = 9.9 Hz, C, 10a-C), 134.06 (d, $^4J_{CF}$ = 3.5 Hz, C, 4a-C), 134.77 (s, CH, 8-C), 137.28 (s, C, 5a-C), 162.00 (d, $^1J_{CF}$ = 251.8 Hz, C, 2-C); ^{19}F-NMR (376.4 MHz, DMSO-d_6) δ_F −98.68 (m, 1F_{Ar}, 2-F); LRMS: *m/z* (ESI) = 336.9, 335.0 $(M + Na)^+$. Compound **8d**: m.p. 282.5–285 °C; ^{1}H-NMR (300 MHz, $CDCl_3$) δ_H 7.49 (d, J = 7.8 Hz, 1H_{Ar}, 1-H), 7.60 (d, J = 8.2 Hz, 1H_{Ar}, 9-H), 7.79 (dd, J = 8.2, 1.8 Hz, 1H_{Ar}, 8-H), 7.94 (d, J = 1.7 Hz, 1H_{Ar}, 6-H), 8.01 (d, J = 6.0 Hz, 1H_{Ar}, 4-H); ^{13}C-NMR (75 MHz, $CDCl_3$) δ_C 110.07 (d, $^2J_{CF}$ = 25.8 Hz, CH, 1-C), 111.88 (d, $^2J_{CF}$ = 12.2 Hz, C, 3-C), 123.28 (s, CH, C-9 [or C-6]), 125.53 (s, C, 7-C), 125.84 (s, CH, C-6 [or C-9]), 128.11 (d, $^3J_{CF}$ = 1.9 Hz, CH, 4-C), 128.75 (s [not resolved], C, 4a-C), 132.68 (d, $^3J_{CF}$ = 8.8 Hz, C, 10a-C), 134.32 (d, $^4J_{CF}$ = 3.8 Hz, C, 9a-C), 137.41 (s, CH, 8-C), 139.67 (s, C, 5a-C), 162.85 (d, J = 256.4 Hz, C, 2-C); ^{19}F-NMR (282.4 MHz, $CDCl_3$) δ_F −98.68 (m, 1 F_{Ar}, 2-F); LRMS: *m/z* (ESI) = 414.9, 416.9, 413.0 $(M + Na)^+$.

Synthesis of **10**, fluorinated reference compounds **10a**–**10c**, **12a**,**b**, **13b**, **14b** and nitro labelling precursor **11a** (for synthesis of [^{18}F]**10a**)

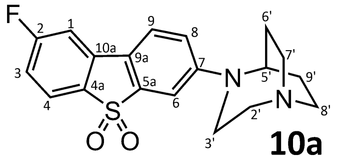

10a

7-(1,4-Diazabicyclo[3.2.2]nonan-4-yl)-2-fluorodibenzo[b,d]thiophene 5,5-dioxide (**10a**). Representative procedure for Buchwald-Hartwig amination. A mixture of $Pd_2(dba)_3$ (11 mg, 0.012 mmol) and BINAP (15 mg, 0.024 mmol) in toluene (1.5 mL) was stirred for 30 min at 90° C. The red-orange colored solution of the catalyst was allowed to cool (22 °C) and added to a mixture of 1,4-diazabicyclo[3.2.2]nonane (**9a**, 53 mg, 0.42 mmol) and **8a** (0.126 g, 0.40 mmol) in toluene (2 mL). Cs_2CO_3 (0.39 g, 1.2 mmol; Alfa Aesar, Karlsruhe, Germany), which was previously dried (4 mbar, 2 h at 120 °C) was then added, and the reaction mixture was stirred under an atmosphere of argon for 24 h at 90° C. After cooling to room temperature, the solid was filtered off and washed with CH_2Cl_2 (2 × 4 mL). The filtrate was evaporated and purified by dry-column flash chromatography (silica gel 15–40 µm, 8 g) with a gradient from $CHCl_3$ (100%) to $CHCl_3$/MeOH/NH_3 (aq) (100:8:0.8, 100%). The fractions containing the product were combined, evaporated and the solid residue was recrystallized from EtOH to afford the title compound **10a** (0.075 g, 0.21 mmol, 52% yield, R_f = 0.18, $CHCl_3$/MeOH/NH_3 (aq), 100:10:1) as yellow crystals, m.p. 317–319 °C (dec.); ^{1}H-NMR (400 MHz, $CDCl_3$) δ_H 1.78 (qd-like, J = 9.7, 4.6 Hz, 2H, 6′-H_a, 9′-H_a), 2.11 (m, 2H, 6′-H_b, 9′-H_b), 2.99 (m, 2H, 7′-H_a, 8′-H_a), 3.09 (A-part of AA′BB′, 2H, 2′-H_2), 3.14 (m, 2H, 7′-H_b, 8′-Hb_a), 3.61 (B-part of AA′BB′, 2H, 3′-H_2), 4.08 (m, not resolved, 1H, 5′-H), 6.89 (dd, J = 8.8, 2.5 Hz, 1H_{Ar}, 8-H), 7.00 (td-like, J = 8.5, 8.5, 2.2 Hz, 1H_{Ar}, 3-H), 7.10 (d, J = 2.5 Hz, 1H_{Ar}, 6-H), 7.24 (dd, J = 8.8, 2.2 Hz, 1H_{Ar}, 1-H), 7.49 (d, J = 8.7 Hz, 1H_{Ar}, 9-H), 7.70 (dd, J = 8.4, 4.9 Hz, 1H_{Ar}, 4-H); ^{13}C-NMR (100 MHz, $CDCl_3$) δ_C 26.77 (s, 2 CH_2, 6′-C, 9′-C), 44.69 (s, CH_2, 3′-C), 46.51 (s, 2 CH_2, 7′-C, 8′-C), 51.74 (s, CH, 5′-C), 57.05 (s, CH_2, 2′-C), 105.19 (s, CH, 6-C), 107.66 (d, $^2J_{CF}$ = 25.1 Hz, CH, 1-C), 114.70 (d, $^2J_{CF}$ = 24.3 Hz, CH, 3-C), 117.03 (s, CH, 8-C), 117.28 (d, $^4J_{CF}$ = 2.2 Hz, C, 9a-C), 123.15 (s, CH, 9-C), 124.27 (d, $^3J_{CF}$ = 10.3 Hz, CH, 4-C), 132.88 (d, $^4J_{CF}$ = 3.0 Hz, C, 4a-C), 136.19 (d, $^3J_{CF}$ = 9.6 Hz, C, 10a-C), 140.61 (s, C, 5a-C), 151.15 (s, C, 7-C), 166.58 (d, $^1J_{CF}$ = 253.6 Hz, C, 2-C); ^{19}F-NMR (282.4 MHz, $CDCl_3$) δ_F −104.5 (m, 1F_{Ar}, 2-F); HRMS *m*/*z* (ESI) calcd for $C_{19}H_{20}FN_2O_2S$ (M + H)$^+$ 359.12240, found 359.12223.

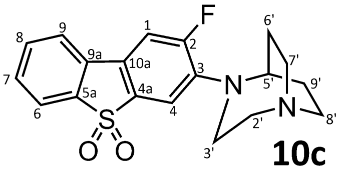

10c

3-(1,4-Diazabicyclo[3.2.2]nonan-4-yl)-2-fluorodibenzo[b,d]thiophene 5,5-dioxide (**10c**). Compound **8c** (0.126 g, 0.4 mmol) and amine **9a** (0.053 g, 0.42 mmol) were reacted in the presence of Cs_2CO_3 (0.39 g, 1.2 mmol) and a catalyst made from $Pd_2(dba)_3$ (11 mg, 0.012 mmol) and BINAP (14.9 mg, 0.024 mmol) according to the representative Buchwald-Hartwig amination as described for **10a**. For purification the same protocol as for **10a** was followed to afford the title compound **10c** (0.063 g, 0.18 mmol, 44% yield, R_f = 0.20, $CHCl_3$/MeOH/NH_3(aq), 100:10:1), as pale yellow crystals, m.p. 305–309 °C (dec.); ^{1}H NMR (300 MHz, $CDCl_3$) δ_H 1.75 (m, 2H, 6′-H_a, 9′-H_a), 2.11 (m, 2H, 6′-H_b, 9′-H_b), 3.04 (m, 4H, 7′-H_2, 8′-H_2), 3.16 (t, J = 5.6 Hz, A-part of AA′BB′, 2H, 2′-H_2), 3.44 (t, J = 5.6 Hz, B-part of AA′BB′, 2H, 3′-H_2), 3.80 (m, 1H, 5′-H), 7.32 (s, 1H_{Ar}, 4-H), 7.36 (d, J = 5.0 Hz, 1H_{Ar}, 1-H), 7.43 (ddd, J = 7.7, 5.1, 3.4 Hz, 1H_{Ar}, 7-H), 7.55–7.63 (m, 2H_{Ar}, 8-H, 9-H), 7.75 (d, J = 7.6 Hz, 1H_{Ar}, 6-H); ^{13}C-NMR (75 MHz, $CDCl_3$) δ_C 27.64 (s, 2 CH_2, 6′-C, 9′-C), 46.70 (s, 2 CH_2, 7′-C, 8′-C), 47.50 (d, $^4J_{CF}$ = 1.2 Hz, CH_2, 3′-C), 55.83 (d, $^4J_{CF}$ = 6.0 Hz, CH, 5′-C), 56.30 (s, CH_2, 2′-C), 109.97 (d, $^2J_{CF}$ = 24.9 Hz, CH, 1-C), 111.82 (d, $^3J_{CF}$ = 5.5 Hz,

CH, 4-C), 120.89 (s, CH, 9-C), 122.19 (s, CH, 6-C), 123.72 (d, $^{3}J_{CF}$ = 9.4 Hz, C, 10a-C), 129.25 (s, CH, 7-C), 131.40 (d, $^{4}J_{CF}$ = 2.3 Hz, C, 4a-C), 133.98 (d, $^{4}J_{CF}$ = 2.9 Hz, C, 9a-C), 134.05 (s, CH, 8-C), 137.87 (s, C, 5a-C), 142.90 (d, $^{2}J_{CF}$ = 10.5 Hz, C, 3-C), 157.62 (d, $^{1}J_{CF}$ = 252.1 Hz, C, 1-C); ^{19}F NMR (282.4 MHz, $CDCl_3$) δ_F −112.95 (m, 1 F_{Ar}, 2-F); HRMS *m*/*z* (ESI) calcd for $C_{19}H_{20}FN_2O_2S$ $(M + H)^+$ 359.12240, found 359.12235.

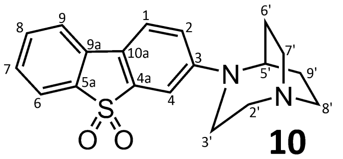

10

3-(1,4-Diazabicyclo[3.2.2]nonan-4-yl)-dibenzo[b,d]thiophene 5,5-dioxide (**10**). Compound **3** (0.22 g, 0.75 mmol) and amine **9a** (0.075 g, 0.6 mmol) were reacted in the presence of Cs_2CO_3 (0.76 g, 2.3 mmol) and a catalyst made from $Pd_2(dba)_3$ (13 mg, 0.012 mmol) and BINAP (17.5 mg, 0.024 mmol) according to the representative Buchwald-Hartwig amination. For purification the same protocol as for **10a** was followed to afford the title compound **10** (0.15 g, 0.44 mmol, 73% yield, R_f = 0.20, $CHCl_3$/MeOH/NH_3 (aq), 100:10:1) as yellow crystals, m.p. 238.5–240 °C (dec.); ^{1}H-NMR (400 MHz, $CDCl_3$) δ 1.76 (qd-like, *J* = 9.6, 4.6 Hz, 2H, 6′-H_a, 9′-H_a), 2.11 (m, 2H, 6′-H_b, 9′-H_b), 2.99 (m, 2H, 7′-H_a, 8′-H_a), 3.09 (A-part of AA′BB′, 2H, 2′-H_2), 3.13 (m, 2H, 7′-H_b, 8′-H_b), 3.60 (B-part of AA′BB′, 2H, 3′-H_2), 4.08 (m, not resolved, 1H, 5′-H), 6.90 (dd, *J* = 8.7, 2.5 Hz, 1H_{Ar}, 8-H), 7.11 (d, *J* = 2.5 Hz, 1H_{Ar}, 6-H), 7.33 (td, *J* = 7.5, 1.0 Hz, 1H_{Ar}, 3-H), 7.53 (td, *J* = 7.6, 1.0 Hz, 1H_{Ar}, 2-H), 7.55 (d, *J* = 8.7 Hz, 1H_{Ar}, 9-H), 7.59 (d, *J* = 7.7 Hz, 1H_{Ar}, 1-H), 7.72 (d, *J* = 7.7 Hz, 1H_{Ar}, 6-H); ^{13}C-NMR (100 MHz, $CDCl_3$) δ_C 26.81 (2 CH_2, 6′-C, 9′-C), 44.66 (CH_2, 3′-C),. 46.54 (2 CH_2, 7′-C, 8′-C), 51.80 (CH, 5′-C), 57.06 (CH_2, 2′-C), 105.31 (CH, 4-C), 117.24 (CH, 2-C), 118.75 (C, 10a-C), 120.19 (CH, 9-C), 122.08 (CH, 6-C), 122.81 (CH, 1-C), 127.86 (CH, 7-C), 132.92 (C, 9a-C), 133.89 (CH, 8-C), 137.10 (C, 5a-C), 139.65 (C, 4a-C), 150.82 (C, 3-C); HRMS *m*/*z* (ESI) calcd for $C_{19}H_{21}N_2O_2S$ $(M + H)^+$ 341.13183, found 341.13141.

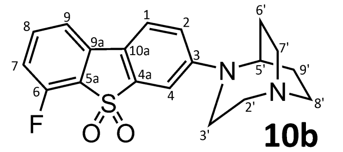

10b

3-(1,4-Diazabicyclo[3.2.2]nonan-4-yl)-6-fluorodibenzo[b,d]thiophene 5,5-dioxide (**10b**). Compound **8b** (0.141 g, 0.45 mmol) and amine **9a** (0.059 g, 0.47 mmol) were reacted in the presence of Cs_2CO_3 (0.44 g, 1.35 mmol) and a catalyst made from $Pd_2(dba)_3$ (10.3 mg, 0.0112 mmol) and BINAP (14 mg, 0.0224 mmol) according to the representative Buchwald-Hartwig amination. For purification the same protocol as for **10a** was followed to afford the title compound **10b** (0.077 g, 0.214 mmol, 48% yield, R_f = 0.19, $CHCl_3$/MeOH/NH_3 (aq), 100:10:1) as yellow crystals, m.p. 272.5–274 °C (dec.); ^{1}H-NMR (300 MHz, $CDCl_3$) δ_H 1.77 (qd-like, *J* = 9.7, 4.6 Hz, 2H, 6′-H_a, 9′-H_a), 2.11 (ddtd, *J* = 12.4, 9.7, 5.0, 2.6 Hz, 2H, 6′-H_b, 9′-H_b), 3.05–2.92 (m, 2H, 7′-H_a, 8′-H_a), 3.20–3.06 (m, A-part of AA′BB′, 4H, 7′-H_b, 8′-Hb_a, 3′-H_2), 3.61 (t-like, *J* = 5.7 Hz, B-part of AA′BB′, 2H, 3′-H_2), 4.08 (m, not resolved, 1H, 5′-H), 6.89 (dd, *J* = 8.8, 2.5 Hz, 1H_{Ar}, 2-H), 6.97 (t, *J* = 8.4 Hz, 1H_{Ar}, 7-H), 7.09 (d, *J* = 2.5 Hz, 1H_{Ar}, 4-H), 7.35 (d, *J* = 7.6 Hz, 1H_{Ar}, 9-H), 7.50 (dt, *J* = 8.0, 5.2 Hz, 1H_{Ar}, 8-H), 7.53 (d, *J* = 8.7 Hz, 1H_{Ar}, 1-H); ^{13}C-NMR (75 MHz, $CDCl_3$) δ_C 26.78 (s, 2 CH_2, 6′-C, 9′-C), 44.70 (s, CH_2, 3′-C), 46.52 (s, 2 CH_2, 7′-C,

8′-C), 51.76 (s, CH, 5′-C), 57.06 (s, CH_2, 2′-C), 105.09 (s, $C_{Ar}H$, 4-C), 115.17 (d, $^2J_{CF}$ = 19.4 Hz, $C_{Ar}H$, 7-C), 115.85 (d, $^4J_{CF}$ = 3.4 Hz, $C_{Ar}H$, 9-C), 117.09 (s, $C_{Ar}H$, 2-C), 117.65 (d, $^4J_{CF}$ = 2.8 Hz, C_{Ar}, 10a-C), 123.25 (s, $C_{Ar}H$, 1-C), 123.63 (d, $^2J_{CF}$ = 17.7 Hz, C_{Ar}, 5a-C), 135.83 (d, $^3J_{CF}$ = 2.7 Hz, C_{Ar}, 9a-C), 136.17 (d, $^3J_{CF}$ = 7.9 Hz, $C_{Ar}H$, 8-C), 140.42 (s, C_{Ar}, 4a-C), 151.12 (s C_{Ar}, 3-C), 158.05 (d, $^1J_{CF}$ = 257.4 Hz, C_{Ar}, 6-C); ^{19}F NMR (282.4 MHz, $CDCl_3$) δ_F −115.66 (m, F_{Ar}, 6-F); HRMS *m/z* (ESI) calcd for $C_{19}H_{20}FN_2O_2S$ $(M + H)^+$ 359.12240, found 359.12235.

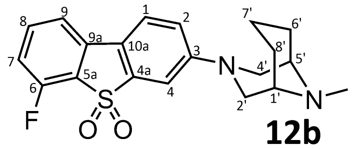

12b

6-Fluoro-3-(9-methyl-3,9-diazabicyclo[3.3.1]nonan-3-yl)dibenzo[b,d]thiophene 5,5-dioxide (**12b**). Compound **8b** (0.132 g, 0.42 mmol) and amine **9b** (0.065 g, 0.46 mmol) were reacted in the presence of Cs_2CO_3 (0.275 g, 0.84 mmol) and a catalyst made from $Pd_2(dba)_3$ (11.5 mg, 0.0126 mmol) and BINAP (15.7 mg, 0.0252 mmol) according to the representative Buchwald-Hartwig amination. For purification the same protocol as for **10a** was followed to afford the title compound **12b** (0.065 g, 0.175 mmol, 42% yield, R_f = 0.31, $CHCl_3$/MeOH/NH_3(aq), 100:10:1) as yellow crystals, m.p. 264–272 °C (dec.); ^{1}H-NMR (300 MHz, $CDCl_3$) δ_H 1.52–1.64 (m, 3H, 7′-H_a, 6′-H_a, 8′-H_a), 1.93–2.15 (m, 3H, 7′-H_b, 6′-H_b, 8′-H_b), 2.59 (s, 3H, 9′-NCH_3), 3.01 (s, 2H, 1′-H, 5′-H), 3.39–3.51 (m, 4H, 2′-H_2, 4′-H_2), 6.97–7.03 (m, 2H_{Ar}, 7-H, 2-H), 7.22 (d, *J* = 2.4 Hz, 1H_{Ar}, 4-H), 7.39 (dd, *J* = 7.7, 0.5 Hz, 1H_{Ar}, 9-H), 7.52 (ddd, *J* = 8.2, 7.8, 5.2 Hz, 1H_{Ar}, 8-H), 7.59 (d, *J* = 8.7 Hz, 1H_{Ar}, 1-H); ^{13}C-NMR (75 MHz, $CDCl_3$) δ_C 18.99 (s, 1C_{sec}, 7′-C), 27.93 (s, 2C_{sec}, 6′-C, 8′-C), 40.62 (s, 1C_{prim}, N-CH_3), 47.40 (s, 2C_{sec}, 2′-C, 4′-C), 53.03 (s, 2C_{tert}, 1′-C, 5′-C), 105.41 (s, 1$C_{Ar}H$, 4-C), 115.46 (d, $^2J_{CF}$ = 19.4 Hz, 1$C_{Ar}H$, 7-C), 116.05 (d, $^4J_{CF}$ = 3.4 Hz, 1$C_{Ar}H$, 9-C), 117.22 (s, 1$C_{Ar}H$, 2-C), 118.83 (d, $^4J_{CF}$ = 2.6 Hz, 1C_{Ar}, 10a-C), 122.99 (s, 1$C_{Ar}H$, 1-C), 123.77 (d, $^2J_{CF}$ = 18.1 Hz, 1C_{Ar}, 5a-C), 135.76 (d, $^3J_{CF}$ = 2.5 Hz, 1C_{Ar}, 9a-C), 136.20 (d, $^3J_{CF}$ = 7.8 Hz, 1$C_{Ar}H$, 8-C), 140.10 (s, 1C_{Ar}, 4a-C), 152.27 (s, 1C_{Ar}, 3-C), 158.06 (d, $^1J_{CF}$ = 257.4 Hz, 1C_{Ar}, 6-C); ^{19}F NMR (282 MHz, $CDCl_3$) δ −115.56 (dd-like, *J* = 8.4, 5.1 Hz, 1F, 6-F); HRMS *m/z* (ESI) calcd for $C_{20}H_{22}FN_2O_2S$ $(M + H)^+$ 373.13805, found 373.13773.

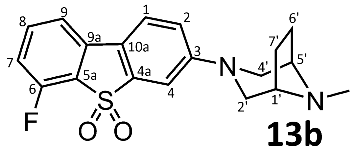

13b

6-Fluoro-3-(8-methyl-3,8-diazabicyclo[3.2.1]octan-3-yl)dibenzo[b,d]thiophene 5,5-dioxide (**13b**). Compound **8b** (0.132 g, 0.42 mmol) and amine **9c** (0.058 g, 0.46 mmol) were reacted in the presence of Cs_2CO_3 (0.358 g, 1.1 mmol) and a catalyst made from $Pd_2(dba)_3$ (11.5 mg, 0.0126 mmol) and BINAP (15.7 mg, 0.0252 mmol) according to the representative Buchwald-Hartwig amination. For purification the same protocol as for **10a** was followed to afford the title compound **13b** (0.071 g, 0.2 mmol, 47% yield, R_f = 0.32, $CHCl_3$/MeOH/NH_3(aq), 100:10:1) as yellow crystals, m.p. 260.5–262.5 °C (dec.); ^{1}H-NMR (400 MHz, $CDCl_3$) δ_H 1.68–1.73 (m, 2H, 6′-H_a, 7′-H_a), 2.05–2.10 (m, 2H, 6′-H_b, 7′-H_b), 2.36 (s, 3H, 8′-NCH_3), 3.15 (dd, *J* = 10.9, 2.2 Hz, 2H, 2′-H_a, 4′-H_a), 3.31 (td, *J* = 4.4, 2.2 Hz, 2H, 1′-H, 5′-H), 3.42 (dd, *J* = 11.0, 2.2 Hz, 2H, 2′-H_b, 4′-H_b), 6.92 (dd, *J* = 8.7, 2.5 Hz, 1H_{Ar}, 2-H), 6.99 (t, *J* = 8.4 Hz,

1H$_{Ar}$, 7-H), 7.14 (d, J = 2.4 Hz, 1H$_{Ar}$, 4-H), 7.37 (d, J = 7.5 Hz, 1H$_{Ar}$, 9-H), 7.50 (ddd, J = 8.3, 7.7, 5.2 Hz, 1H$_{Ar}$, 8-H), 7.55 (d, J = 8.7 Hz, 1H$_{Ar}$, 1-H); ^{13}C-NMR (101 MHz, CDCl$_3$) δ$_C$ 25.79 (s, 2C$_{sec}$, 6′-C, 7′-C), 40.41 (s, 1C$_{prim}$, N-CH$_3$), 53.10 (s, 2C$_{sec}$, 2′-C, 4′-C), 60.22 (s, 2C$_{tert}$, 1′-C, 5′-C), 105.49 (s, 1C$_{Ar}$H, 4-C), 115.50 (d, $^2J_{CF}$ = 19.4 Hz, 1C$_{Ar}$H, 7-C), 116.07 (d, $^4J_{CF}$ = 3.3 Hz, 1C$_{Ar}$H, 9-C), 117.24 (s, 1C$_{Ar}$H, 2-C), 119.06 (d, $^4J_{CF}$ = 2.7 Hz, 1C$_{Ar}$, 10a-C),122.94 (s, 1C$_{Ar}$H, 1-C), 123.78 (d, $^2J_{CF}$ = 18.3 Hz, 1C$_{Ar}$, 5a-C), 135.67 (d, $^3J_{CF}$ = 2.6 Hz, 1C$_{Ar}$, 9a-C), 136.19 (d, $^3J_{CF}$ = 7.9 Hz, 1C$_{Ar}$H, 8-C), 139.99 (s, 1C$_{Ar}$, 4a-C), 153.26 (s, 1C$_{Ar}$, 3-C), 158.03 (d, $^1J_{CF}$ = 257.5 Hz, 1C$_{Ar}$, 6-C); ^{19}F-NMR (376 MHz, CDCl$_3$) δ −115.57 (dd, J = 8.5, 5.2 Hz); HRMS m/z (ESI) calcd for $C_{19}H_{20}FN_2O_2S$ (M + H)$^+$ 359.12240, found 359.12211.

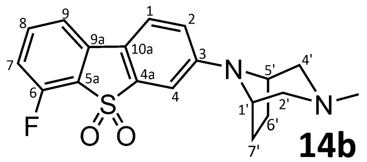

14b

6-Fluoro-3-(3-methyl-3,8-diazabicyclo[3.2.1]octan-8-yl)dibenzo[b,d]thiophene 5,5-dioxide (**14b**). Compound **8b** (0.132 g, 0.42 mmol) and amine **9d** (0.058 g, 0.46 mmol) were reacted in the presence of Cs_2CO_3 (0.358 g, 1.1 mmol) and a catalyst made from $Pd_2(dba)_3$ (11.5 mg, 0.0126 mmol) and BINAP (15.7 mg, 0.0252 mmol) according to the representative Buchwald-Hartwig amination. For purification the same protocol as for **10a** was followed to afford the title compound **14b** (0.101 g, 0.28 mmol, 67% yield, R$_f$ = 0.32, $CHCl_3$/MeOH/NH_3(aq), 100:10:1) as yellow crystals, m.p. 254.5–256 °C (dec.); ^{1}H-NMR (300 MHz, CDCl$_3$) δ$_H$ 1.93–2.13 (m, 4H, 6′-H$_2$, 7′-H$_2$), 2.17 (s, 3H, 8′-NCH$_3$), 2.38 (dd, J = 11.1, 1.3 Hz, 2H, 2′-H$_a$, 4′-H$_a$), 2.58 (dd, J = 11.0, 2.3 Hz, 2H, 2′-H$_b$, 4′-H$_b$), 4.23 (m, 2H, 1′-H, 5′-H), 6.88 (dd, J = 8.7, 2.4 Hz, 1H$_{Ar}$, 2-H), 6.98 (td, J = 8.4, 0.6 Hz, 1H$_{Ar}$, 7-H), 7.07 (d, J = 2.3 Hz, 1H$_{Ar}$, 4-H), 7.36 (dd, J = 7.7, 0.6 Hz, 1H$_{Ar}$, 9-H), 7.50 (ddd, J = 8.3, 7.7, 5.0 Hz, 1H$_{Ar}$, 8-H), 7.54 (d, J = 8.6 Hz, 1H$_{Ar}$, 1-H); ^{13}C NMR (75 MHz, CDCl$_3$) δ$_C$ 28.20 (s, 2C$_{sec}$, 6′-C, 7′-C), 45.44 (s, 1C$_{prim}$, N-CH$_3$), 55.65 (s, 2C$_{tert}$, 1′-C, 5′-C), 57.29 (s, 2C$_{sec}$, 2′-C, 4′-C), 107.17 (s, 1C$_{Ar}$H, 4-C), 115.26 (d, $^2J_{CF}$ = 19.4 Hz, 1C$_{Ar}$H, 7-C), 115.90 (d, $^4J_{CF}$ = 3.4 Hz, 1C$_{Ar}$H, 9-C), 118.12 (d, $^4J_{CF}$ = 2.7 Hz, 1C$_{Ar}$, 10a-C), 119.19 (s, 1C$_{Ar}$H, 2-C), 123.51 (s, 1C$_{Ar}$H, 1-C), 123.84 (d, $^2J_{CF}$ = 13.8 Hz, 1C$_{Ar}$, 5a-C), 135.85 (d, $^3J_{CF}$ = 2.6 Hz, 1C$_{Ar}$, 9a-C), 136.18 (d, $^3J_{CF}$ = 7.9 Hz, 1C$_{Ar}$H, 8-C), 140.57 (s, 1C$_{Ar}$, 4a-C), 148.99 (s, 1C$_{Ar}$, 3-C), 158.05 (d, $^1J_{CF}$ = 257.5 Hz, 1C$_{Ar}$, 6-C); ^{19}F-NMR (282 MHz, CDCl$_3$) δ −115. 67 (m, F$_{Ar}$, 6-F); HRMS m/z (ESI) calcd for $C_{19}H_{20}FN_2O_2S$ (M + H)$^+$ 359.12240, found 359.12237.

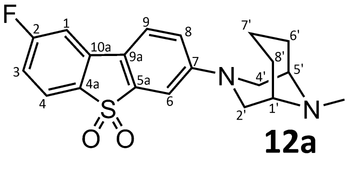

12a

2-Fluoro-7-(9-methyl-3,9-diazabicyclo[3.3.1]nonan-3-yl)dibenzo[b,d]thiophene 5,5-dioxide (**12a**). Compound **8a** (0.132 g, 0.42 mmol) and amine **9b** (0.065 g, 0.46 mmol) were reacted in the presence of Cs_2CO_3 (0.358 g, 1.1 mmol) and a catalyst made from $Pd_2(dba)_3$ (11.5 mg, 0.0126 mmol) and BINAP (15.7 mg, 0.0252 mmol) according to the representative Buchwald-Hartwig amination. For purification the same protocol as for **10a** was followed to afford the title compound **12a** (0.06 g, 0.2 mmol, 38% yield, R$_f$ = 0.33, $CHCl_3$/MeOH/NH_3(aq), 100:10:1) as yellow crystals, m.p. 214–217 °C (dec.); ^{1}H-NMR

(400 MHz, $CDCl_3$) δ_H 1.58–1.65 (m, 3H, 7′-H_a, 6′-H_a, 8′-H_a), 1.99–2.16 (m, 3H, 7′-H_b, 6′-H_b, 8′-H_b), 2.62 (s, 3H, 9′-NCH_3), 3.05 (br, 2H, 1′-H, 5′-H), 3.45 (dd, J = 11.7, 3.6 Hz, 2H, 2′-H_a, 4′-H_a), 3.52 (d, J = 11.7 Hz, 2H, 2′-H_b, 4′-H_b), 7.01–7.09 (m, 2H_{Ar}, 8-H, 3-H), 7.24–7.33 (m, 2H_{Ar}, 6-H, 1-H), 7.58 (d, J = 8.7 Hz, 1H_{Ar}, 9-H), 7.75 (dd, J = 8.4, 4.9 Hz, 1H_{Ar}, 4-H); ^{13}C-NMR (101 MHz, $CDCl_3$) δ_C 18.97 (s, 1C_{sec}, 7′-C), 27.94 (s, 2C_{sec}, 6′-C, 8′-C), 40.62 (s, 1C_{prim}, N-CH_3), 47.34 (s, 2C_{sec}, 2′-C, 4′-C), 53.01 (s, 2C_{tert}, 1′-C, 5′-C), 105.48 (s, CH, 6-C), 107.87 (d, $^2J_{CF}$ = 24.7 Hz, CH, 1-C), 115.00 (d, $^2J_{CF}$ = 24.0 Hz, CH, 3-C), 117.17 (s, CH, 8-C), 118.46 (d, $^4J_{CF}$ = 2.3 Hz, C, 9a-C), 122.88 (s, CH, 9-C), 124.32 (d, $^3J_{CF}$ = 10.1 Hz, CH, 4-C), 132.98 (d, $^4J_{CF}$ = 2.8 Hz, C, 4a-C), 136.12 (d, $^3J_{CF}$ = 10.1 Hz, C, 10a-C), 140.25 (s, C, 5a-C), 152.29 (s, C, 7-C), 166.56 (d, $^1J_{CF}$ = 253.8 Hz, C, 2-C); ^{19}F-NMR (377 MHz, $CDCl_3$) δ_F −104.01 (m, F_{Ar}, 2-F); HRMS m/z (ESI) calcd for $C_{20}H_{22}FN_2O_2S$ $(M + H)^+$ 373.13805, found 373.13813.

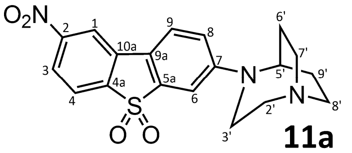

11a

7-(1,4-Diazabicyclo[3.2.2]nonan-4-yl)-2-nitrodibenzo[b,d]thiophene 5,5-dioxide (**11a**). Compound **6a** (0.17 g, 0.5 mmol) and amine **9a** (0.064 g, 0.505 mmol) were reacted in the presence of Cs_2CO_3 (0.49 g, 1.5 mol) and a catalyst mixture made from $Pd_2(dba)_3$ (18.3 mg, 0.02 mmol) and BINAP (25 mg, 0.04 mmol) according to the representative Buchwald-Hartwig amination as described for **10a**. Purification via dry-column flash chromatography (silica gel 15–40 µm, 9 g) with a gradient from $CHCl_3$ (100%) to $CHCl_3$/MeOH/NH_3(aq) (100:8:0.8, 100%) afforded the title compound **11a** (0.103 g, 0.27 mmol, 53% yield, R_f = 0.21, $CHCl_3$/MeOH/NH_3(aq), 100:10:1) as red crystals, m.p. 291–294 °C (dec.); ^{1}H-NMR (300 MHz, $CDCl_3$) δ_H 1.79 (qd-like, J = 9.6, 4.6 Hz, 2H, 6′-H_a, 9′-H_a), 2.12 (m, 2H, 6′-H_b, 9′-H_b), 2.99 (m, 2H, 7′-H_a, 8′-H_a), 3.10 (A-part of AA′BB′, 2H, 2′-H_2), 3.13 (m, 2H, 7′-H_b, 8′-H_{ba}), 3.65 (B-part of AA′BB′, 2H, 3′-H_2), 4.11 (m, not resolved, 1H, 5′-H), 6.94 (dd, J = 8.8, 2.6 Hz, 1H_{Ar}, 8-H), 7.11 (d, J = 2.5 Hz, 1H_{Ar}, 6-H), 7.63 (d, J = 8.8 Hz, 1H_{Ar}, 9-H), 7.85 (d, J = 8.3 Hz, 1H_{Ar}, 4-H), 8.15 (dd, J = 8.3, 2.0 Hz, 1H_{Ar}, 3-H), 8.35 (d, J = 1.9 Hz, 1H_{Ar}, 1-H); ^{13}C-NMR (75 MHz, $CDCl_3$) δ_C 26.70 (2 CH_2, 6′-C, 9′-C), 44.71 (CH_2, 3′-C), 46.47 (2 CH_2, 7′-C, 8′-C), 51.72 (CH, 5′-C), 57.02 (CH_2, 2′-C), 105.18 (CH, 6-C), 115.16 (CH, 8-C), 116.04 (1 C_{Ar}, 9a-C), 117.33 (CH, 1-C), 122.59 (CH, 9-C [or 3-C, 4-C]), 123.18 (CH, 4-C [or 3-C, 9-C]), 123.67 (CH, 3-C [or 9-C, 4-C]), 135.15 (C, 10a-C), 140.17 (C, 5a-C), 141.86 (C, 4a-C), 151.46 (C, 7-C), 151.82 (C, 2-C); HRMS m/z (ESI) calcd for $C_{19}H_{20}N_3O_4S$ $(M + H)^+$ 386.11690, found 386.11704.

Images of NMR Spectra

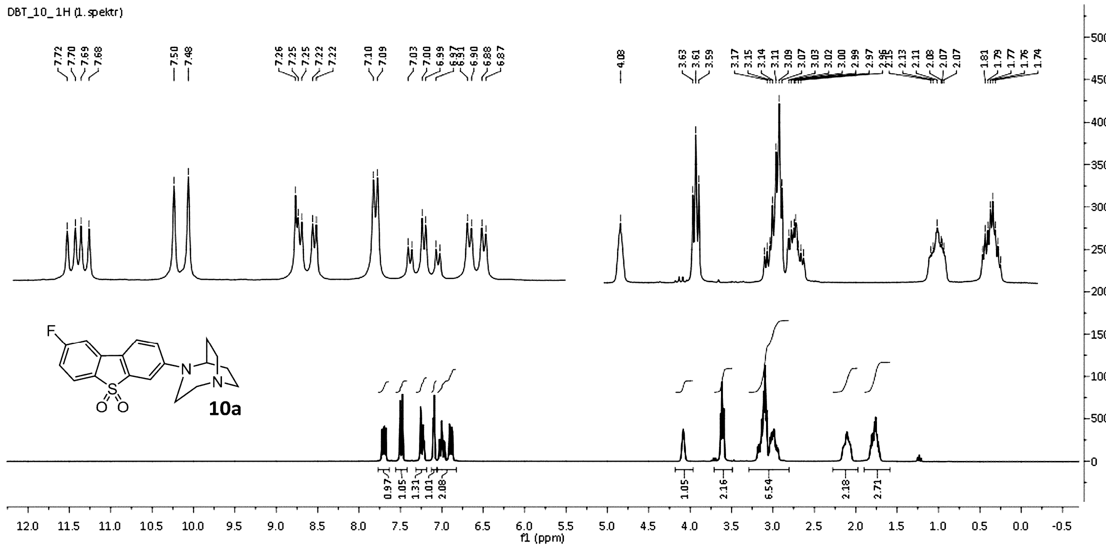

Figure S1. ^{1}H-NMR spectrum of compound **10a** in $CDCl_3$.

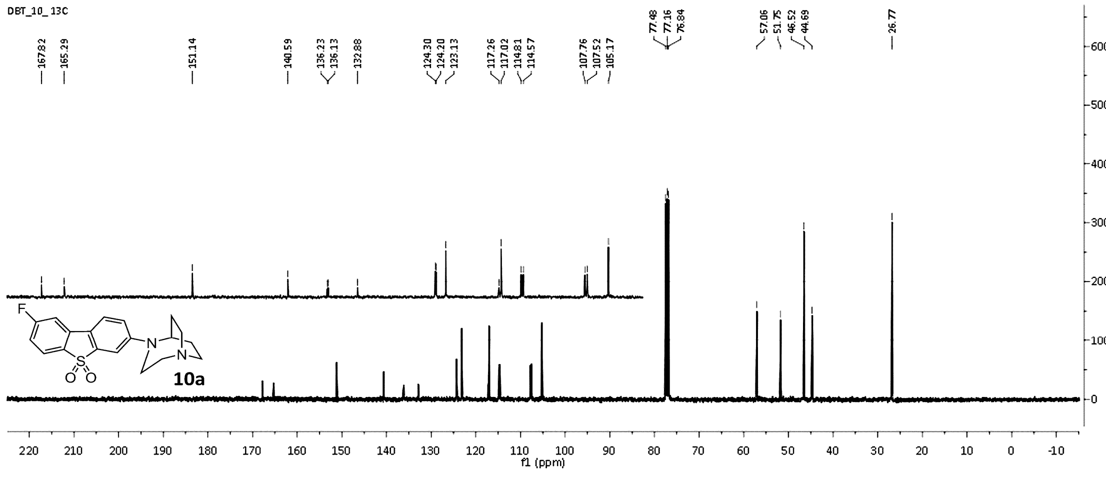

Figure S2. ^{13}C-NMR-spectrum of compound **10a** in $CDCl_3$.

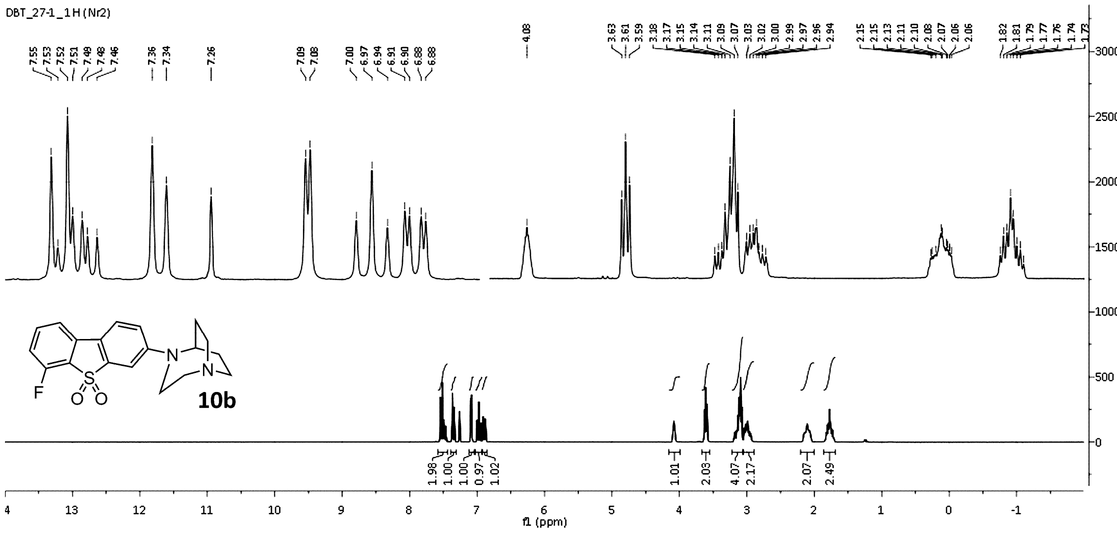

Figure S3. ^{1}H-NMR spectrum of compound **10b** in $CDCl_3$.

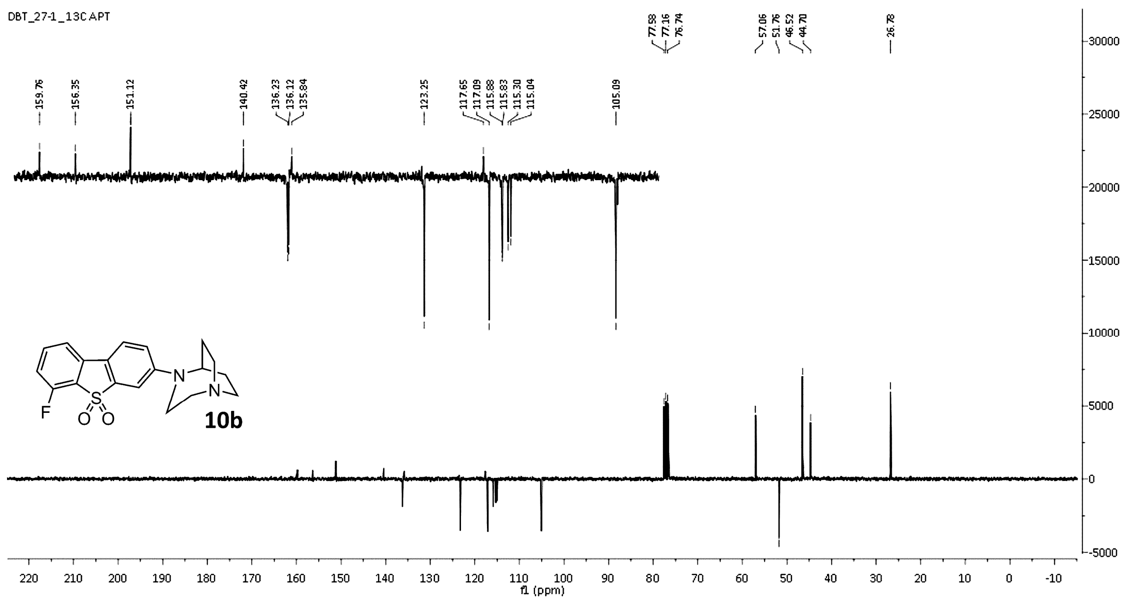

Figure S4. ^{13}C-NMR-APT spectrum of compound **10b** in $CDCl_3$.

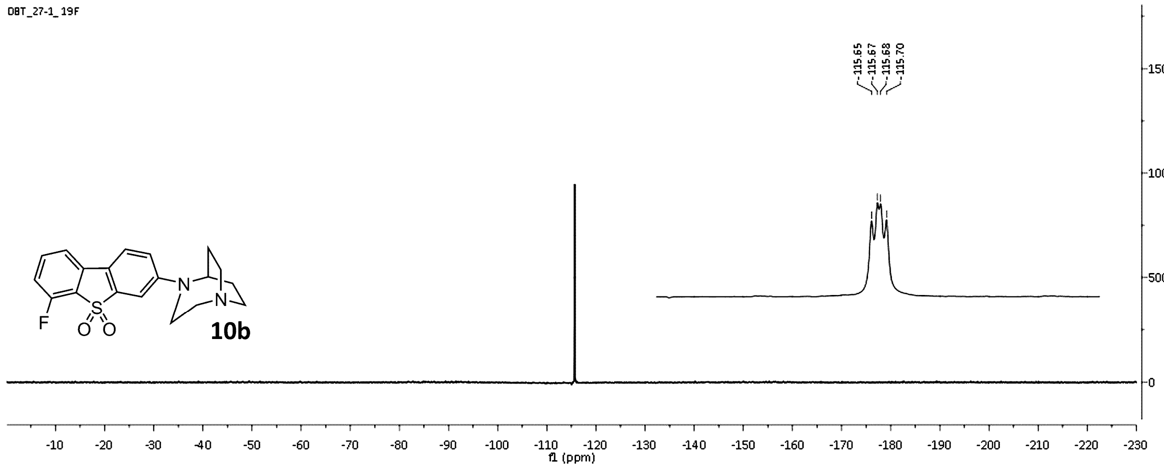

Figure S5. ^{19}F-NMR spectrum of compound **10b** in $CDCl_3$.

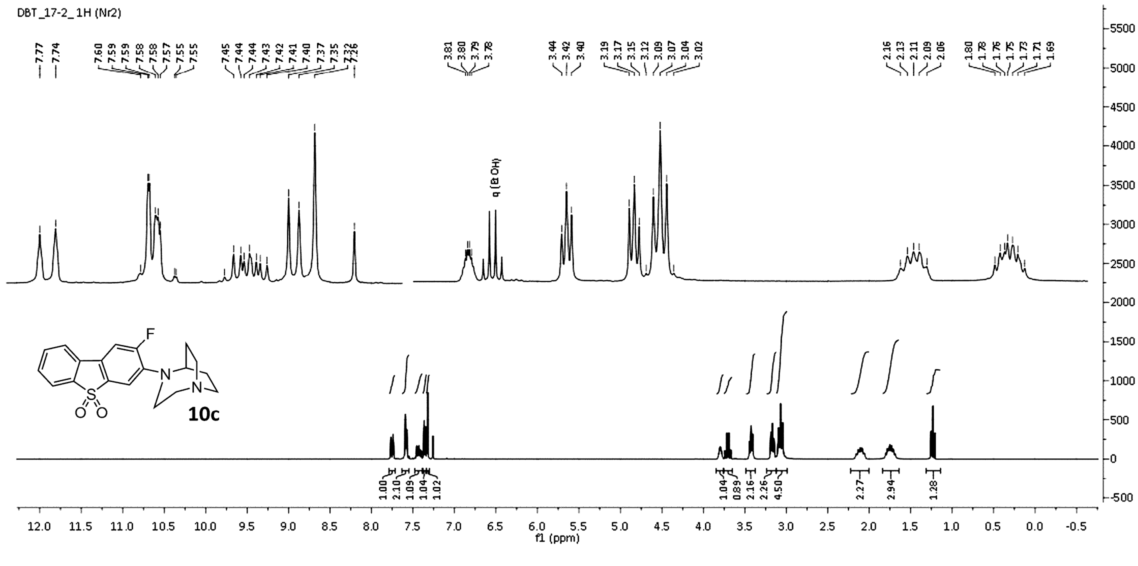

Figure S6. ^{1}H-NMR spectrum of compound **10c** in $CDCl_3$.

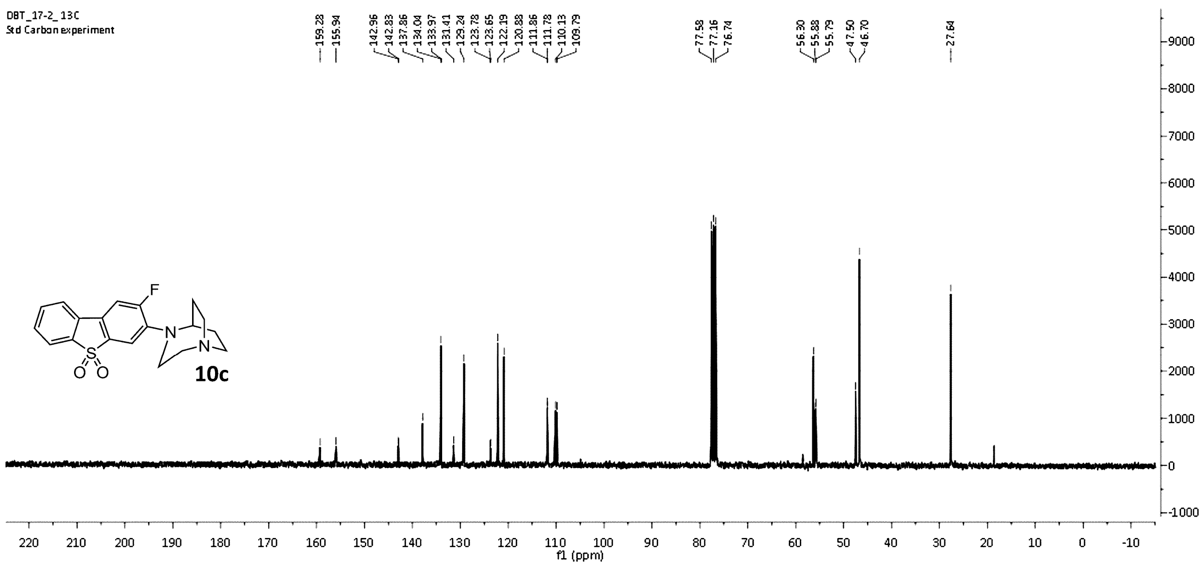

Figure S7. ^{13}C-NMR spectrum of compound **10c** in $CDCl_3$.

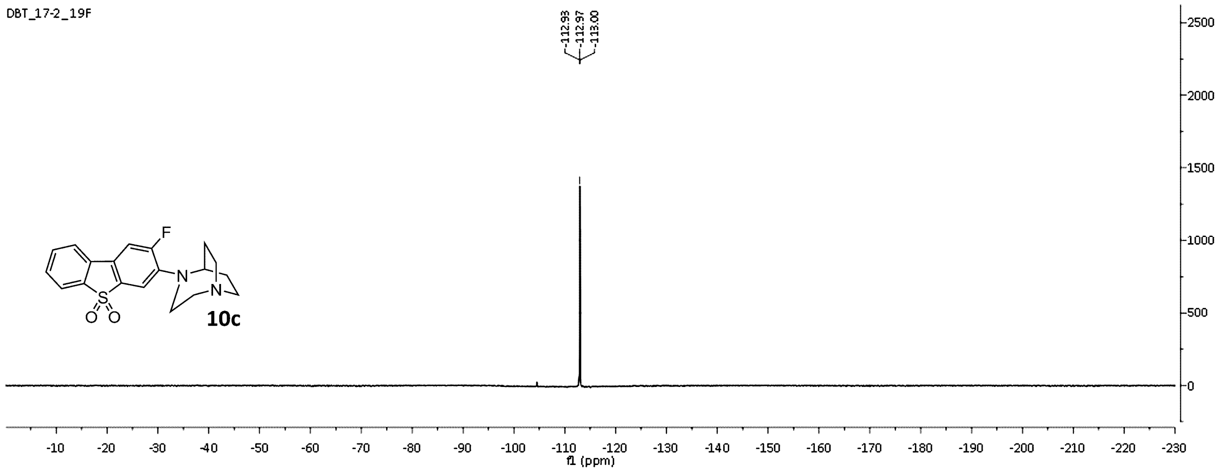

Figure S8. ^{19}F-NMR spectrum of compound **10c** in $CDCl_3$.

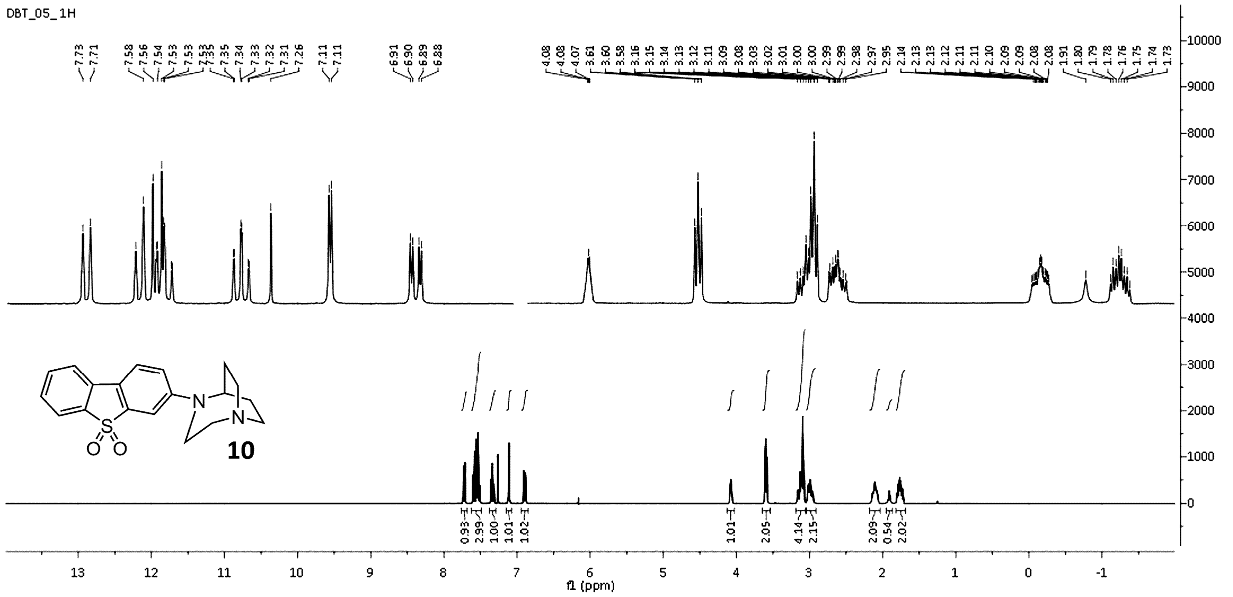

Figure S9. ^{1}H-NMR spectrum of compound **10** in $CDCl_3$.

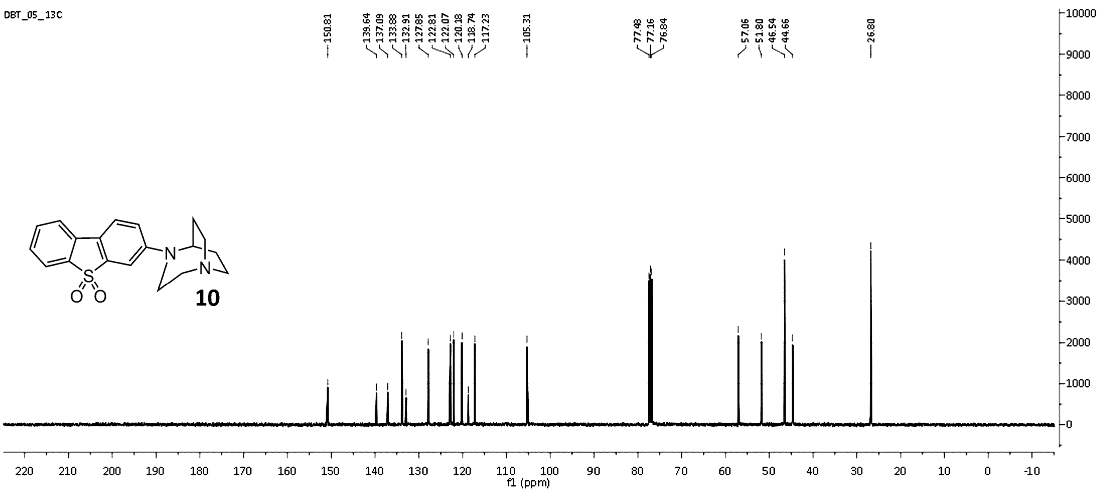

Figure S10. ^{13}C-NMR spectrum of compound **10** in $CDCl_3$.

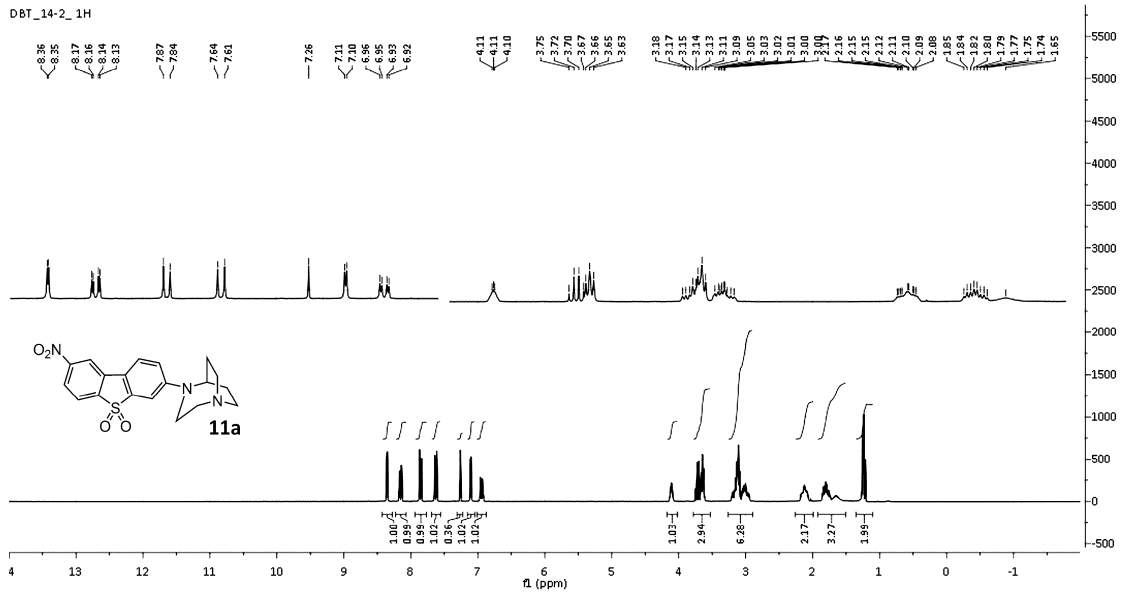

Figure S11. ^{1}H-NMR spectrum of compound **11a** in $CDCl_3$.

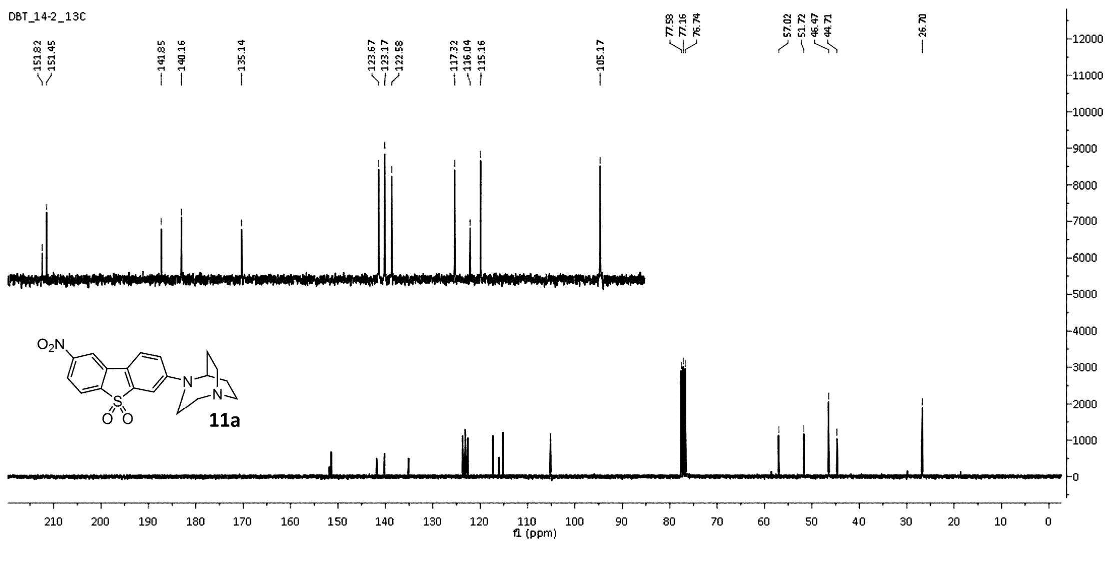

Figure S12. ^{13}C-NMR spectrum of compound **11a** in $CDCl_3$.

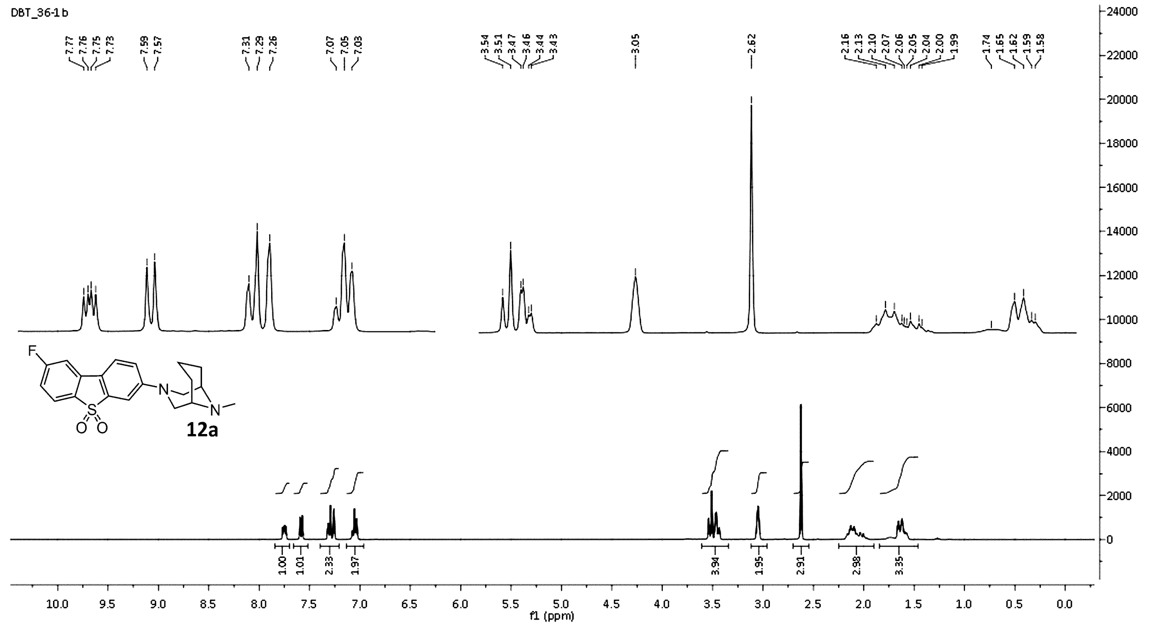

Figure S13. ^{1}H-NMR spectrum of compound **12a** in $CDCl_3$.

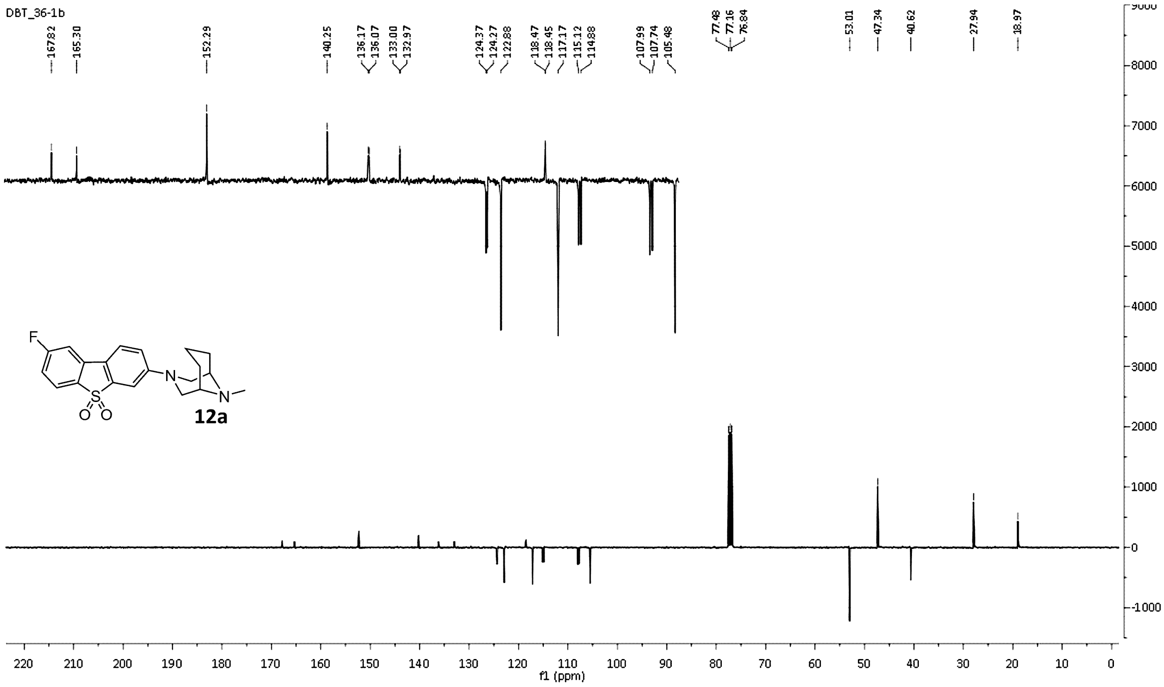

Figure 14. ^{13}C-APT NMR spectrum of compound **12a** in $CDCl_3$.

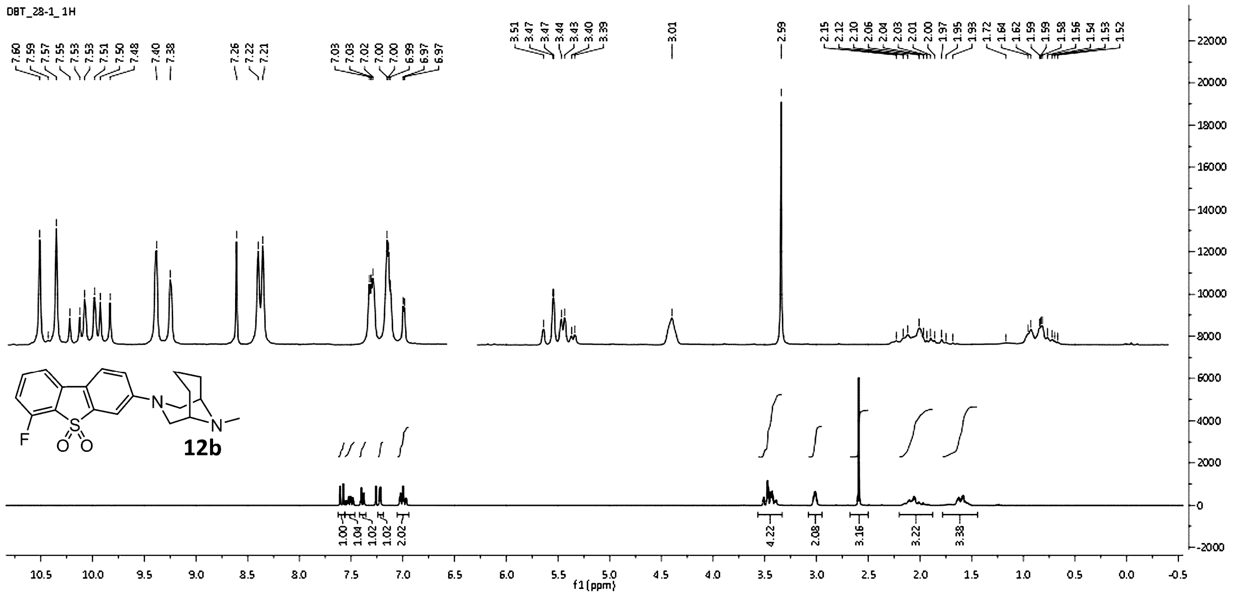

Figure S15. ^{1}H-NMR spectrum of compound **12b** in $CDCl_3$.

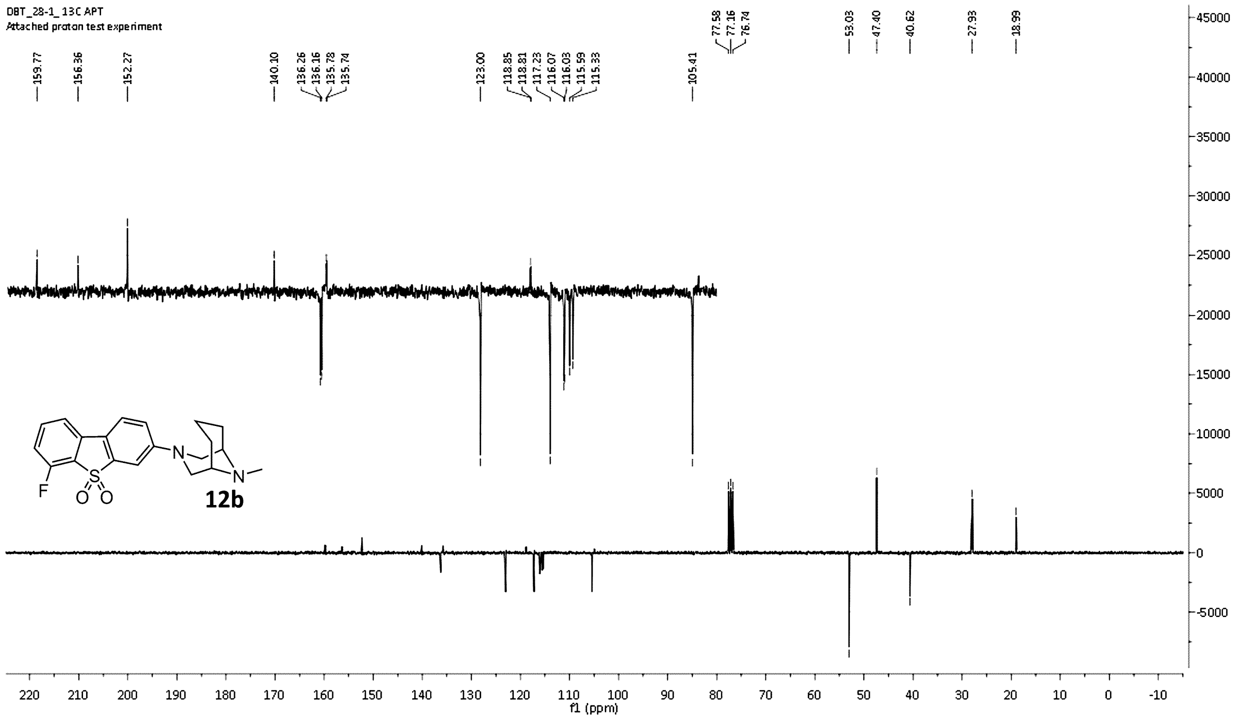

Figure S16. ^{13}C-APT NMR spectrum of compound **12b** in $CDCl_3$.

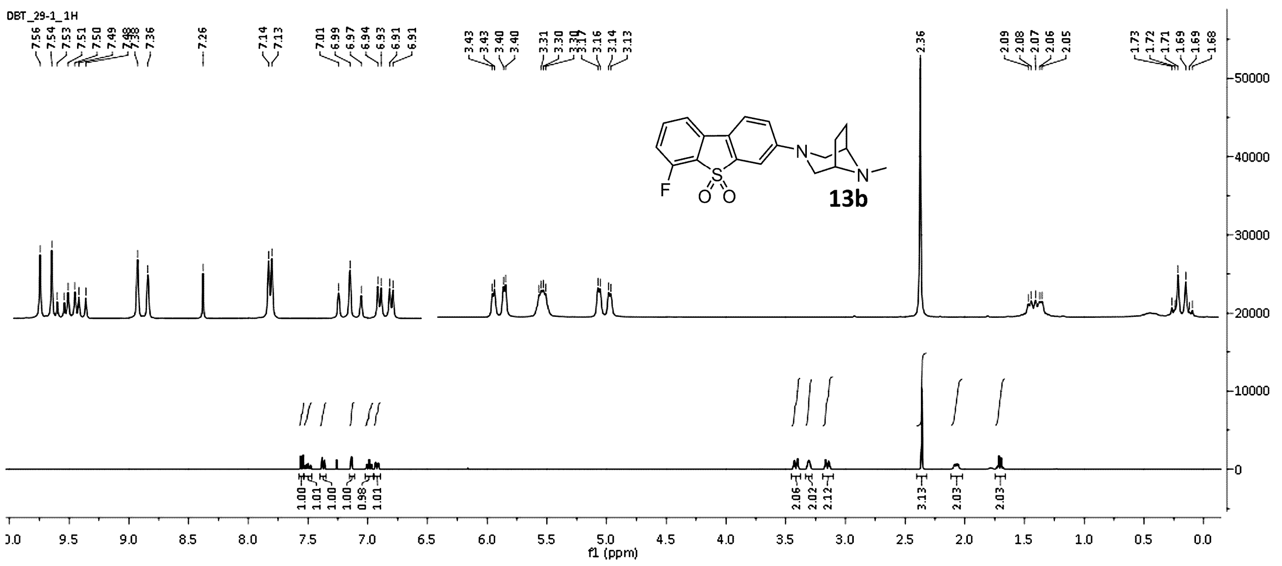

Figure S17. ^{1}H-NMR spectrum of compound **13b** in $CDCl_3$.

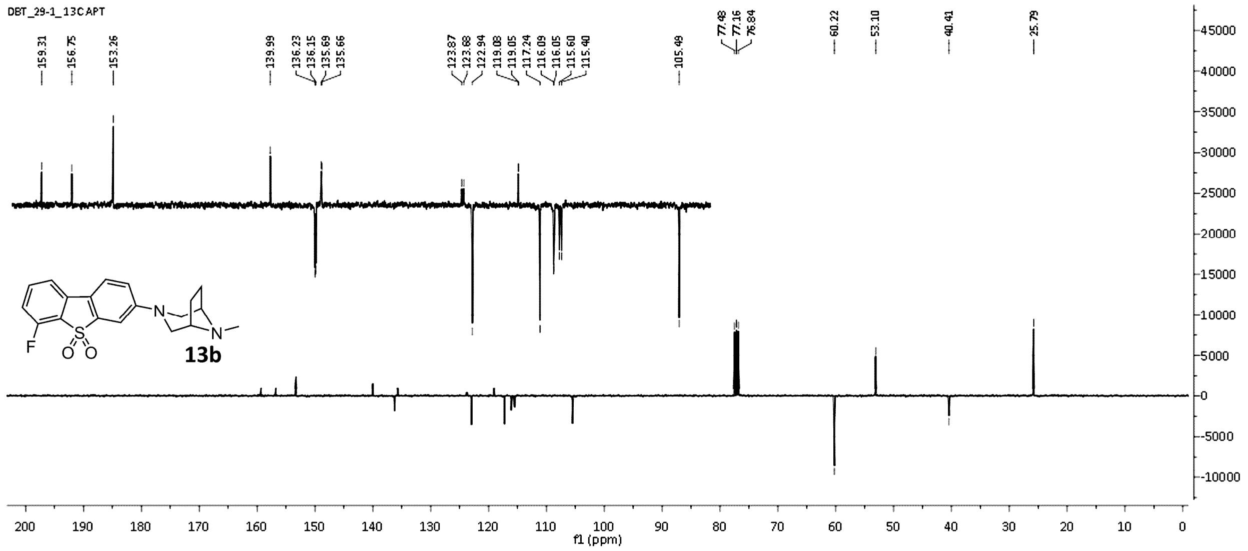

Figure S18. ^{13}C-APT NMR spectrum of compound **13b** in $CDCl_3$.

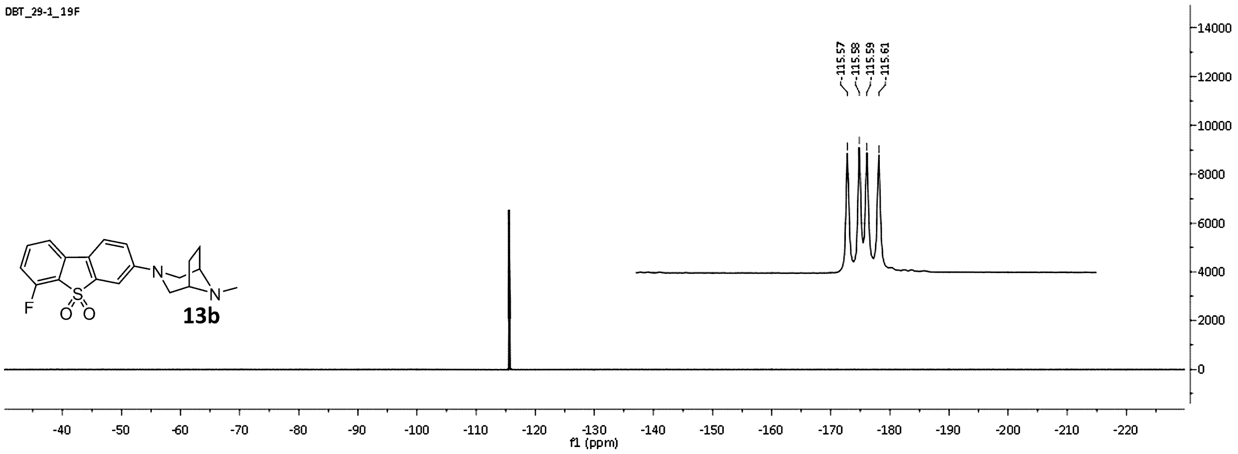

Figure S19. ^{19}F-NMR spectrum of compound **13b** in $CDCl_3$.

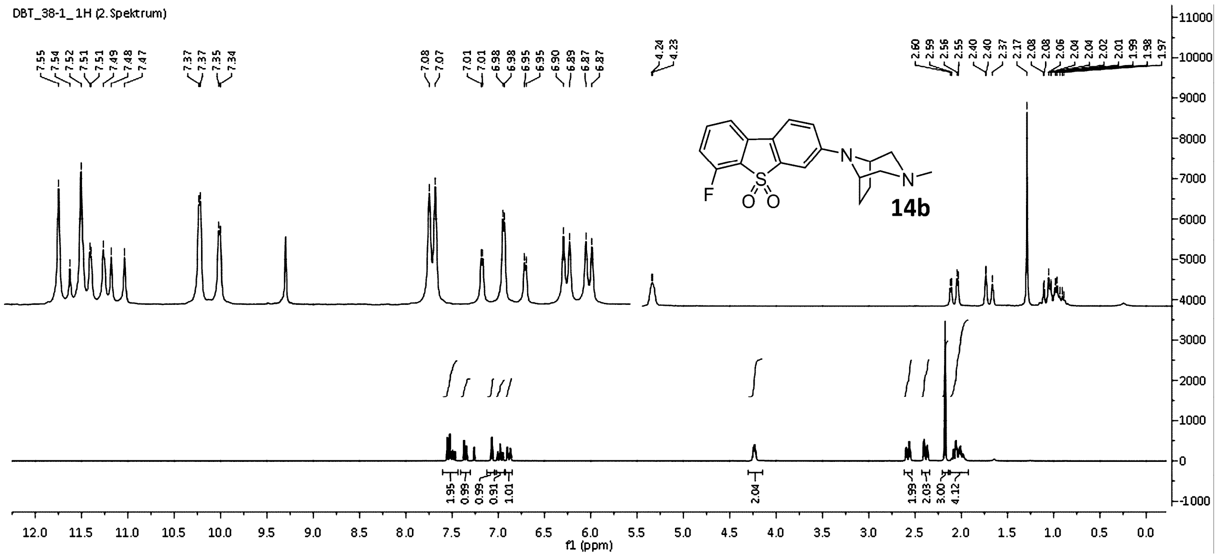

Figure S20. ^{1}H-NMR spectrum of compound **14b** in $CDCl_3$.

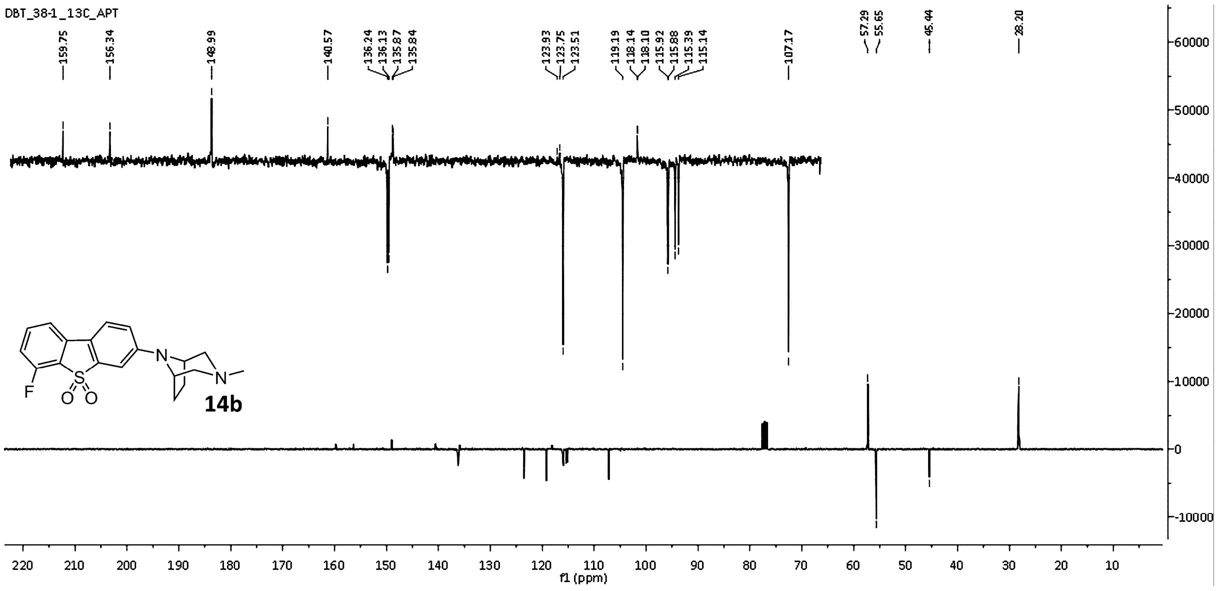

Figure S21. ^{13}C-APT NMR spectrum of compound **14b** in $CDCl_3$.

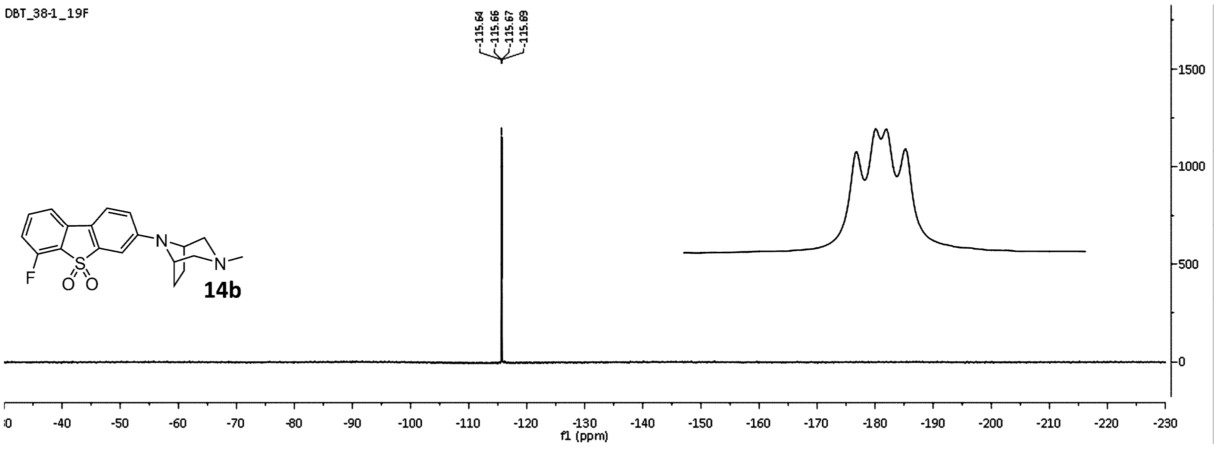

Figure S22. ^{19}F-NMR spectrum of compound **14b** in $CDCl_3$.

References

1. Pelletier, S.W.; Chokshi, H.P.; Desai, H.K. Separation of diterpenoid alkaloid mixtures using vacuum liquid chromatography. *J. Nat. Prod*. **1986**, *49*, 892–900.
2. Coll, J.C.; Bowden, B.F. The application of vacuum liquid chromatography to the separation of terpene mixtures. *J. Nat. Prod*. **1986**, *49*, 934–936.
3. Harwood, L.M. "Dry-column" flash chromatography. *Aldrichimica Acta* **1985**, *18*, 25.
4. Pedersen, D.S.; Rosenbohm, C. Dry column vacuum chromatography. *Synthesis* **2001**, *16*, 2431–2434.
5. Gilman, H.; Esmay, D.L. The oxidation of dibenzothiophene and phenoxathiin with hydrogen peroxide. *J. Am. Chem. Soc*. **1952**, *74*, 2021–2024.
6. Butts, C.P.; Eberson, L.; Hartshorn, M.P.; Radner, F.; Robinson, W.T.; Wood, B.R. Regiochemistry of the reaction between dibenzothiophene radical cation and nucleophiles or nitrogen dioxide. *Acta Chem. Scand*. **1997**, *51*, 839–848.
7. Gilman, H.; Nobis, J.F. Some aminodibenzothiophenes. *J. Am. Chem. Soc.* **1949**, *71*, 274–276.
8. Cullinane, N.M.; Davies, C.G.; Davies, G.I. Substitution derivatives of diphenylene sulphide and diphenylenesulphone. *J. Chem. Soc*. **1936**, 1435–1437.
9. Brown, R.K.; Nelson, N.A.; Wood, J.C. Nitro and amino derivatives of dibenzothiophene. *J. Am. Chem. Soc*. **1952**, *74*, 1165–1167.
10. Gilman, H.; Avakian, S. Some dialkylaminoalkylamino derivatives of dibenzothiophene. *J. Am. Chem. Soc*. **1946**, *68*, 1514–1515.
11. Gilman, H.; Jacoby, A.L.; Pacevitz, H.A. Relative reactivities of organometallic compounds. XVIII. Selective metalations of dibenzothiophene. *J. Org. Chem*. **1938**, *3*, 120–124.
12. Gao, Y.; Kellar, K.J.; Yasuda, R.P.; Tran, T.; Xiao, Y.; Dannals, R.F.; Horti, A.G. Derivatives of dibenzothiophene for positron emission tomography imaging of α_7-nicotinic acetylcholine receptors. *J. Med. Chem*. **2013**, *56*, 7574–7589.
13. Schrimpf, M.R.; Sippy, K.B.; Ji, J.; Li, T.; Frost, J.M.; Briggs, C.A.; Bunnelle, W.H. Amino-Substituted Tricyclic Derivatives and Methods of Use. U.S. Patent 2005234031 A1, 20 October 2005.
14. Perepichka, I.I.; Perepichka, I.F.; Bryce, M.R.; Palsson, L.-O. Dibenzothiophene-*S*,*S*-dioxide-fluorene co-oligomers. Stable, highly-efficient blue emitters with improved electron affinity. *Chem. Commun*. **2005**, 3397 – 3399, doi:10.1039/B417717G.
15. Cavalcanti, L.N.; Molander, G.A. Nitrosation of aryl and heteroaryltrifluoroborates with nitrosonium tetrafluoroborate. *J. Org. Chem*. **2012**, *77*, 4402–4413.
